# Ppm level palladium catalyzed regioselective remote arylation of alkenyl alcohols†

**DOI:** 10.1039/d5sc02745d

**Published:** 2025-06-03

**Authors:** Chong Liu, Ling Wang, Haibo Ge

**Affiliations:** a Department of Chemistry and Biochemistry, Texas Tech University Lubbock Texas 79409 USA Haibo.Ge@ttu.edu; b Residual Department, Merieux Testing Technology (Qingdao) Co., Ltd Qingdao 266000 China

## Abstract

Recent studies highlight the importance and application of parts per million (ppm) palladium concentration in catalytic reactions. Lowering catalyst loading minimizes costs, simplifies purification, and reduces metal contamination, making it highly attractive for pharmaceutical and fine chemical manufacturing. Here, we report a ppm level Pd-catalyzed remote arylation reaction of alkenols, achieving high efficiency, regioselectivity, and flexible carbonyl scaffold construction. Notably, this strategy exhibits excellent compatibility with styrene-derived substrates and has been successfully achieved on a gram scale, providing a solid foundation for potential large-scale applications.

## Abstract 2

A ppm-level Pd-catalyzed remote arylation reaction of alkenols was reported with high catalytic efficiency, excellent regioselectivity, and broad substrate compatibility, thus enabling the efficient access to diverse carbonyl-containing frameworks.

## Graphical abstract

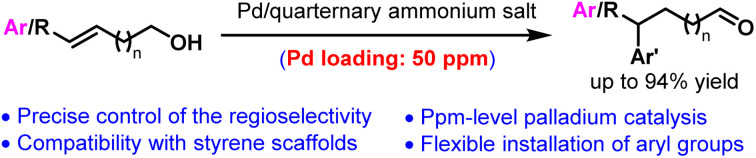

## Introduction

Palladium-catalyzed cross coupling reactions have emerged as indispensable tools in organic synthesis, enabling the construction of complex molecular architectures with exceptional efficiency and broad functional group tolerance.^1^ Despite extensive efforts in developing alternative catalytic systems based on earth-abundant metals^2^ such as nickel,^3^ iron,^4^ manganese,^5^ copper^6^ and cobalt,^7^ palladium remains the preferred catalyst due to its unparalleled reactivity, selectivity, and stability across diverse reaction conditions. However, the high cost, combined with stringent regulations on metal residues in active pharmaceutical ingredient (API) synthesis, presents significant challenges for high-loading Pd catalytic processes.^8^ These challenges not only increase production risks but also impose substantial economic burdens on downstream purification. As a result, the development of highly efficient catalytic methodologies that minimize catalyst loadings without compromising reaction performance has become imperative.

**Scheme 1:**
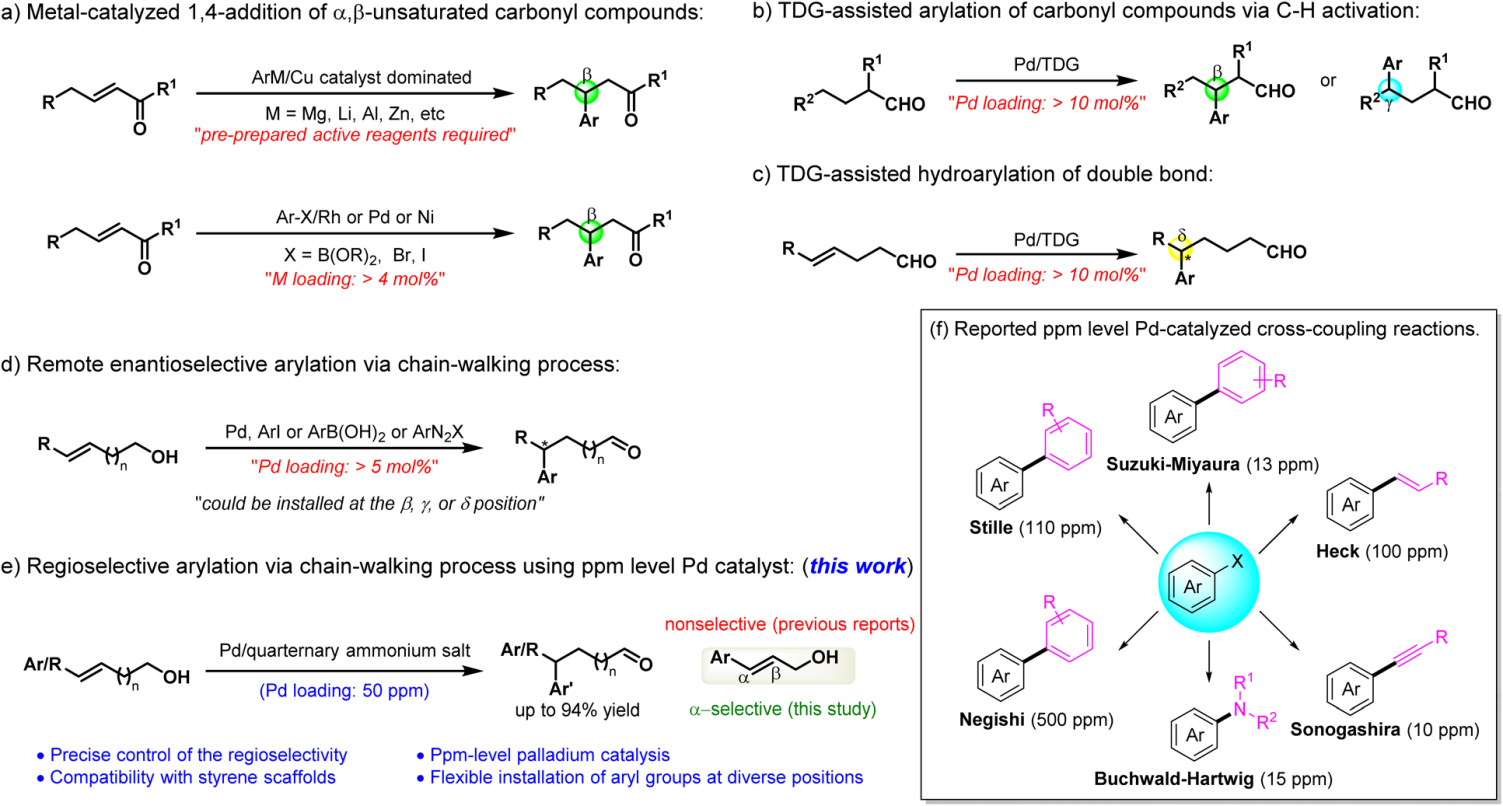
Approaches to the construction of aryl-substituted carbonyl compounds.

Recent advancements have demonstrated the feasibility of conducting cross-coupling and related transformations with palladium at ppm levels, offering a promising approach to significantly reducing catalyst consumption without sacrificing efficiency (Scheme 1f).^9^ Reducing Pd loading to such minimal levels not only mitigates cost-related concerns but also streamlines purification, minimizes residual metal contamination, and enhances the overall sustainability of catalytic processes. While earth-abundant metal catalysts have gained attention,^2^ their widespread adoption remains hindered by several intrinsic limitations, including the requirement for high catalyst loading, restricted functional group compatibility, and suboptimal reactivity under mild conditions. Therefore, achieving efficient catalysis with ppm level palladium represents a crucial step toward more cost-effective and environmentally sustainable industrial processes.

Distal aryl-substituted carbonyl compounds serve as valuable building blocks in pharmaceuticals, agrochemicals, and materials science. Moreover, the high derivatization potential of the carbonyl functionality renders these compounds crucial intermediates in organic synthesis, enabling the construction of structurally diverse and functionally complex molecules.^10^ Despite their importance, developing practical and efficient methods for the synthesis of distal aryl-substituted carbonyl compounds remains a challenge. Transition metal-catalyzed cross-coupling reactions have emerged as powerful tools for assembling arylated carbonyl structures. It has been well-established that metal-catalyzed 1,4-addition of α,β-unsaturated carbonyl compounds enables the efficient construction of β-arylated products (Scheme 1a).^11^ The Ge^12^ and Yu^13^ groups have successfully achieved β- or γ-arylation of carbonyl compounds *via* C–H functionalization using a transient directing group (TDG) strategy (Scheme 1b). Using a similar approach, Zhang and co-workers have employed alkenyl substrates to realize hydroarylation of double bonds, leading to δ-arylated products (Scheme 1c).^14^ However, these methodologies exhibit notable limitations: requiring high palladium loadings (>10 mol%) and being restricted to specific reaction sites, thereby limiting the structural diversity of the products.

The chain-walking strategy has emerged as a highly versatile and powerful approach for achieving site-diverse functionalization, attracting considerable attention in recent years.^15^ The Sigman group has demonstrated the feasibility of this concept by employing arylboronic acids or aryl diazonium salts as coupling partners to construct structurally diverse arylated carbonyl compounds (Scheme 1d).^16^ More recently, Ge and co-workers expanded this strategy using more cost-effective and safer aryl iodides, successfully achieving site-diverse arylation reactions (Scheme 1d).^10^ Nevertheless, the same major challenge remains—palladium loading exceeding 5 mol%. To address this issue, we turn our attention to the use of quaternary ammonium salts, which are well known to enhance the catalytic activity by effectively dispersing Pd catalysts.^17^ Leveraging this principle, various coupling reactions have been successfully achieved with ultra-low, even ppm-level Pd loadings.^18^ However, regioselective coupling reactions, particularly those relying on chain-walking strategies, remain unexplored under such minimal catalyst usage. Building on these findings, we sought to harness the dispersive effect of quaternary ammonium salts to develop a ppm-level Pd-catalyzed remote arylation of alkenyl alcohols with varying chain lengths (Scheme 1e).

## Results and discussion

The initial investigation was conducted with 1-(benzyloxy)-4-iodobenzene (1a), (*Z*)-pent-2-en-1-ol (2a), Pd(OAc)_2_ (50 ppm), and tetrabutylammonium tetrafluoroborate (TBA·BF_4_) in toluene at 125 °C for 24 h. A survey of bases revealed that sodium formate (HCOONa) was optimal, delivering the desired product 3a in 41% yield, with an excellent regioselectivity of 15 : 1. Subsequent investigations on quaternary ammonium salts indicated that tetrabutylammonium bromide (TBAB) worked best, improving the yield to 95% (for details on conditions screening, see Table S1 in the ESI†). Control experiments revealed that no desired product was detected in the absence of either Pd or the quaternary ammonium salt, effectively ruling out the contribution of trace Pd impurities in the reagents to the observed reactivity (Table 1, entries 8 and 9). Given the crucial role of these ammonium salts, we speculate that palladium nanoparticles (Pd NPs) are likely formed and serve as the active catalytic species in this case. This hypothesis is supported by extensive reports demonstrating that quaternary ammonium salts can effectively stabilize Pd NPs, preventing their aggregation and subsequent deactivation, thereby enhancing catalytic performance.^17^ Additionally, we evaluated varying Pd catalyst loadings and found that at 50 ppm, the starting material (1a) was nearly completely consumed. Further increasing the Pd loading resulted in no significant improvement in yield.

**Table 1:** Optimization of reaction conditions^a^

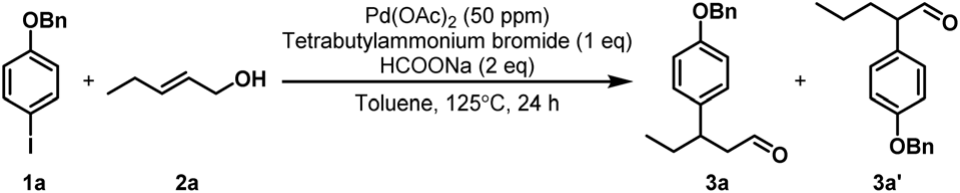			
Entry	Changes from standard conditions^a^	Ratio (3a/3a′)	Yield of 3a^b^ (%)
1	None	>20 : 1	95 (92)
2	HCOOK instead of HCOONa	17 : 1	79
3	HCOOLi instead of HCOONa	14 : 1	91
4	Tetrahexylammonium tetrafluoroborate instead of TBAB	>20 : 1	21
5	Tetrapropylammonium tetrafluoroborate instead of TBAB	—	Trace
6	TBAC instead of TBAB	>20 : 1	46
7	TBAI instead of TBAB	—	Trace
8	Without TBAB	—	N.D.
9	Without Pd(OAc)_2_	—	N.D.
10	Pd(OAc)_2_ (25 ppm)	>20 : 1	87
11	Pd(OAc)_2_ (75 ppm)	>20 : 1	96
12	Pd(OAc)_2_ (100 ppm)	18 : 1	96

a Conditions: 1a (0.1 mmol), 2a (0.2 mmol), Pd(OAc)_2_ (50 ppm), quaternary ammonium salt (0.1 mmol), base (0.2 mmol), toluene (1 mL), 125 °C, 24 h, unless otherwise noted.

b Determined by QNMR with 1,3,5-trimethoxybenzene as an internal standard, the number in parentheses is the isolated yield. TBA·BF_4_ = tetrabutylammonium tetrafluoroborate. TBAC = tetrabutylammonium chloride. TBAB = tetrabutylammonium bromide. TBAI = tetrabutylammonium iodide.

**Scheme 2:**
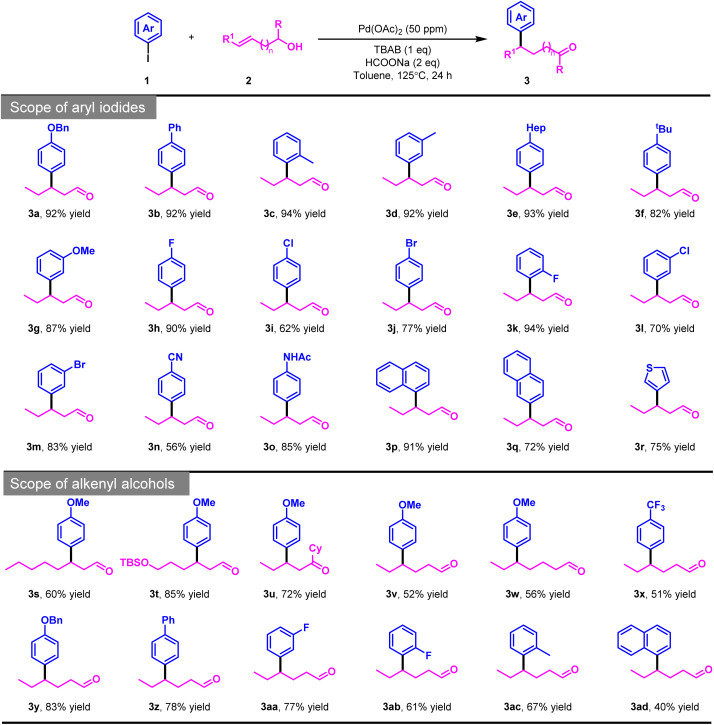
Substrate scope. Reaction conditions: 1a (0.2 mmol), 2a (0.4 mmol), Pd(OAc)_2_ (50 ppm), TBAB (0.2 mmol), HCOONa (0.4 mmol), toluene (2 mL), 125 °C, 24 h, unless otherwise noted, isolated yields. Hep = heptyl group.

With an optimized protocol obtained, a wide range of aryl iodides were then evaluated (Scheme 2). In most cases, the corresponding products can be obtained with highly satisfactory yields, regardless of the electron properties and positions of the substituents. This catalytic system also exhibits good tolerance toward thiophene substrates (3r), while aryl iodides containing a pyridine scaffold fail to produce the desired products, likely due to the strong coordination of the pyridine nitrogen to the palladium center, resulting in deactivation of the trace amounts of the Pd catalyst. Next, we surveyed the scope with respect to the alkenol coupling partners. Notably, alkenyl alcohols with varying chain lengths can afford aldehyde products in satisfactory yields (3v, 3w). When alkenyl alcohols with two carbons between the double bond and the hydroxyl group are used, various types of aryl iodides efficiently participate in remote arylation reactions (3x–3ad).

**Scheme 3:**
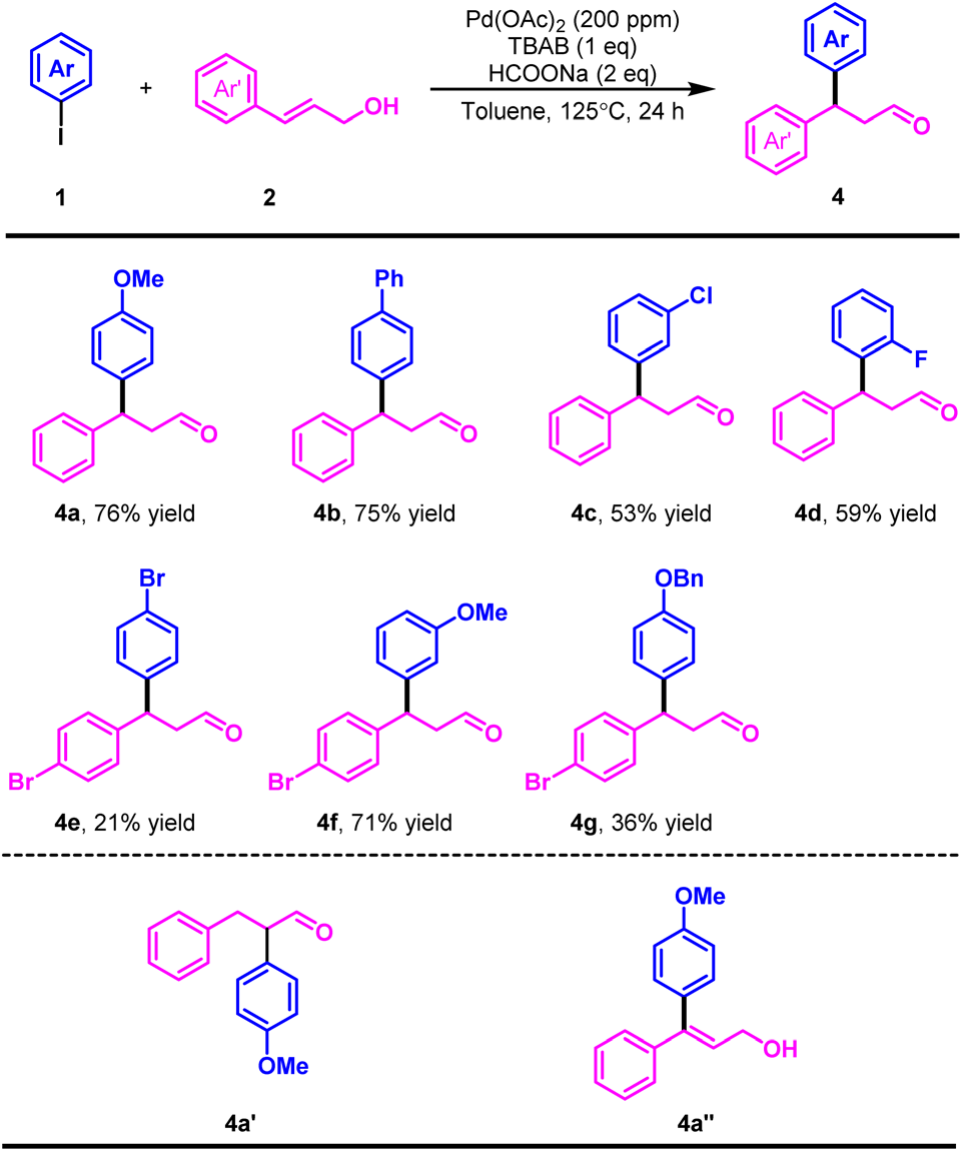
Substrate scope of alkenols bearing a styrene moiety. Reaction conditions: 1a (0.2 mmol), 2a (0.6 mmol), Pd(OAc)_2_ (200 ppm), TBAB (0.2 mmol), HCOONa (0.4 mmol), toluene (2 mL), 125 °C, 24 h, unless otherwise noted, isolated yields.

Alkenols bearing a styrene moiety present a significant challenge in chain-walking Heck-type processes due to inherent site-selectivity issues arising from competing insertion and β-H elimination pathways. To the best of our knowledge, the construction of carbonyl compounds *via* a chain-walking strategy has not been explored for this class of substrates.^10,16^ Motivated by this gap, we investigated their compatibility with our catalytic system. Encouragingly, under the optimal conditions, (*E*)-3-phenylprop-2-en-1-ol (2b) underwent efficient transformation to afford the desired product (4a) in 10% yield with a regioselectivity (4a/4a′) of 10 : 1, and the formation of the side product 4a′′ was nearly undetectable. Increasing the catalyst loading to 200 ppm successfully improved the yield to 76%. Encouraged by these results, we next explored the substrate scope by evaluating a range of alkenols and coupling partners (Scheme 3). Different types of aryl iodides and 4-bromo-substituted cinnamyl alcohol are well compatible with this catalytic system, affording the corresponding diaryl-substituted aldehyde compounds with satisfactory yields.

**Scheme 4:**
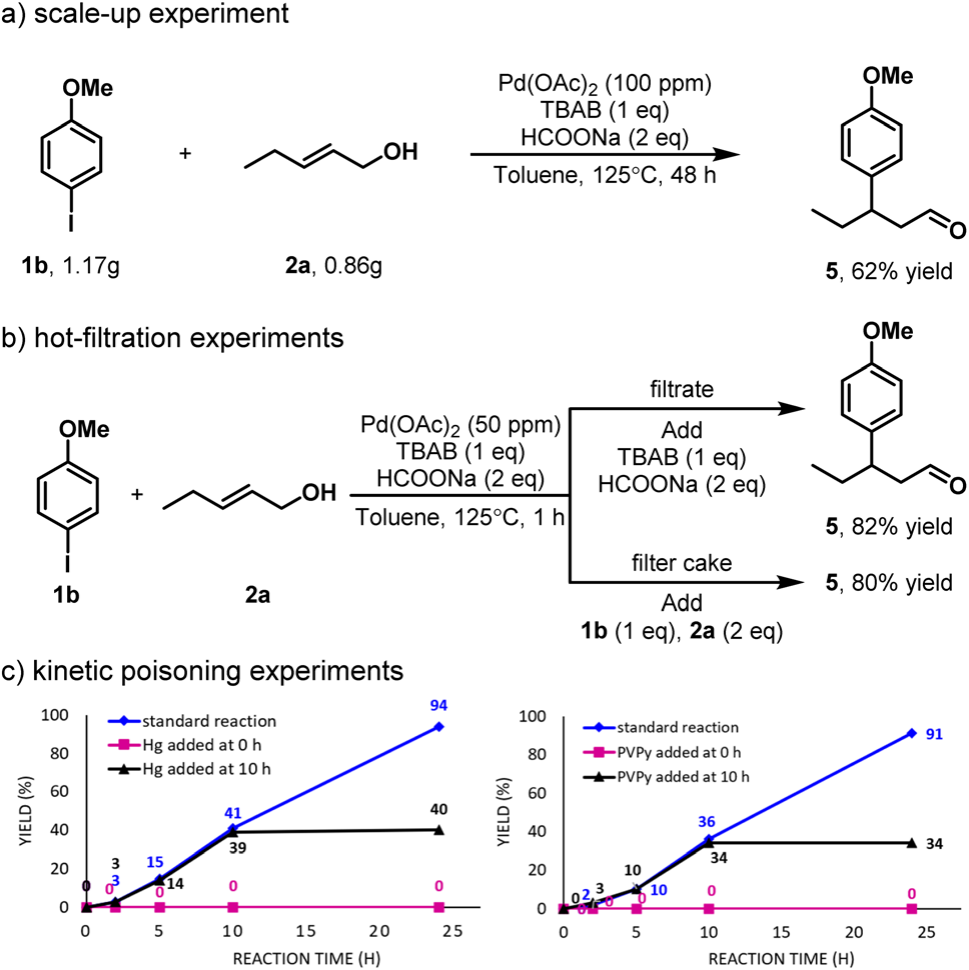
Gram-scale experiment and mechanism studies.

To further demonstrate the synthetic utility of this transformation, we conducted a gram-scale experiment. Under modified conditions, the desired product was obtained in an impressive 62% yield, highlighting the scalability of this methodology (Scheme 4a). To gain some insight into the nature of the catalytic system, we conducted a hot filtration experiment. The results suggested that the reaction may proceed through a dual homogeneous and heterogeneous catalytic pathway (Scheme 4b).^19^ In addition, kinetic poisoning experiments were performed to clarify the nature of the catalyst species (Scheme 4c).^20^ The addition of mercury (20 equivalents) at the beginning of the reaction or after 10 hours led to an alteration in the catalytic activity, indicating the possible involvement of nanoparticle-based catalysis.^21^ Furthermore, poly(4-vinylpyridine) (PVPy), which was reported to selectively deactivate homogeneous palladium catalysts without affecting heterogeneous ones,^22^ caused an immediate loss of reactivity when introduced at either 0 or 10 hours (20 equivalents). These findings support the operation of a homogeneous catalytic mechanism.

Furthermore, extensive efforts have also been made to visualize Pd nanoparticles by TEM and DLS analysis under standard conditions, but no well-defined particles were observed. This is not entirely unexpected, as quaternary ammonium salts are known to be able to stabilize Pd species in a highly dispersed, dynamic state.^17^ However, the suppression of catalytic activity upon Hg addition suggested that at least part of the catalysis involves Pd(0) species with accessible metal surfaces, potentially in the form of soluble clusters or dynamic nanostructures, despite their invisibility under TEM or DLS. This behaviour aligned with reports of “cocktail”-type systems^23^ or “homeopathic catalysis”,^24^ where the ligandless species existed in a dynamic equilibrium with soluble Pd clusters and nanoparticles. This phenomenon is particularly common in Pd-catalyzed processes involving oxidative addition of aryl halides, where soluble Pd clusters or nanoparticles act as dynamic reservoirs of catalytically active species, where the arylating agent interacts with surface-exposed Pd atoms, particularly those located at the periphery of the clusters or nanoparticles.^25^ Taken together, these results suggested that catalysis in our system likely arises from highly dispersed or soluble Pd(0) species, possibly as soluble Pd clusters, rather than from bulk or insoluble nanoparticles.

## Conclusions

In conclusion, we have, for the first time, developed the ppm-level Pd-catalyzed remote arylation of alkenols, achieving exceptional catalytic efficiency and high regioselectivity. This strategy enables the flexible construction of diverse carbonyl-containing scaffolds through an efficient chain-walking process. Notably, alkenols bearing a styrene moiety, which have previously posed challenges in such transformations, exhibit excellent compatibility with this catalytic system. Furthermore, success on the gram scale underscores its potential for practical and large-scale applications.

## Author contributions

H. G. conceived and supervised the project. C. L. performed the experimental studies. L. W. carried out the TEM and DLS tests. C. L. wrote the original draft of the manuscript which was revised by all authors.

## Conflicts of interest

There are no conflicts to declare.

## Supplementary Material

SC-016-D5SC02745D-s001

## Data Availability

All experimental and characterization data, as well as NMR spectra, are available in the ESI.†
