# Supplementary material for: Ppm level palladium catalyzed regioselective remote arylation of alkenyl alcohols
Source: Chem Sci. 2025 Jun 3;16(27):12449–54. doi: 10.1039/d5sc02745d (PMC12147037; doi:10.1039/d5sc02745d)

Supplementary Information (SI) for Chemical Science.
This journal is © The Royal Society of Chemistry 2025

Ppm Level Palladium Catalyzed Regioselective Remote Arylation of Alkenyl Alcohols

Table of Contents

1. General Details..S2
2. Condition Optimization S3
3. Substrate scope of the remote arylation reaction S4
4. Substrate scope on styrene-derived alkenols...S11
5. Mechanism studies...S13
6. NMR Spectra S14

1. General Details

All reactions were performed in dry glassware or in a glove box under an atmosphere of nitrogen. The workup was carried out in air, unless otherwise noted. Column chromatography was performed using silica gel. Solvents were dried and distilled before use by standard procedures. Commercially available reagents were used without further purification.

^{1}H NMR (400 MHz), ^{13}C NMR (101 MHz) and ^{19}F NMR (376 MHz) spectra were recorded on a JEOL ECS 400 MHz NMR Spectrometer. MS was performed on Agilent 7890 GC Mass Spectrometry.

2. Condition Optimization

Table S1. Conditions optimization on remote arylation.

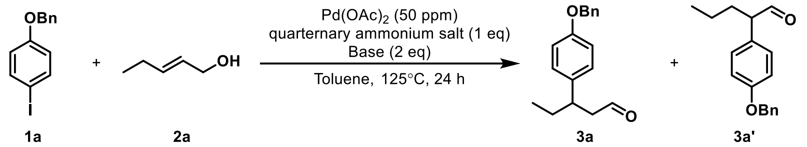

entry[a]	Ammonium salt	Base	Ratio of 3a/3a'	Yield (%)[b]
1	TBA·BF_4	KOAc	> 20:1	21
2	TBA·BF_4	NaOAc	-	trace
3	TBA·BF_4	Na_2CO_3	> 20:1	30
4	TBA·BF_4	K_2CO_3	-	trace
5	TBA·BF_4	Na_2HPO_4	-	N.D.
6	TBA·BF_4	K_2HPO_4	-	trace
7	TBA·BF_4	HCOOK	12:1	24
8	TBA·BF_4	HCOOLi	10:1	37
9	TBA·BF_4	HCOONa	15:1	41
10	TBA·PF_6	HCOONa	-	trace
11	TBA·OMs	HCOONa	13:1	56
12	TBA·NO_3	HCOONa	10:1	82
13	TBA·OBz	HCOONa	> 20:1	20
14	TBA·ClO_4	HCOONa	-	trace
15	TBA·OTs	HCOONa	9:1	73
16	TBA·HSO_4	HCOONa	15:1	36
17	TBA·H_2PO_4	HCOONa	> 20:1	18
18	benzyl triethyl ammonium chloride	HCOONa	-	trace

[a]Conditions: **1a** (0.1 mmol), **2a** (0.2 mmol), $Pd(OAc)_2$ (50 ppm), quarternary ammonium salt (1 eq), base (2 eq), Toluene (1 mL), 125 ˚C, 24 h. [b]Determined by QNMR with 1,3,5-trimethoxybenzene as internal standard.

3. Substrate scope of the remote arylation reaction

General Procedure: 1 (0.2 mmol, 1 eq), $Pd(OAc)_2$ (50 ppm), tetrabutylammonium bromide (0.2 mmol, 1 eq) and sodium formate (0.4 mmol, 2 eq) was added to a Schlenk tube equipped with a magnetic stirrer. The tube was degassed and refilled with nitrogen for three times. A solution of **2** (0.4 mmol, 2 eq) in toluene (2 mL) was introduced and the tube was sealed. The solution was allowed to stir for 30 min at rt and then heated to 125 °C and stirred for another 24 h. After reaction was completed, cooled to rt and filtered. The resulting solution was concentrated and purified through column chromatography with hexane/EtOAc as eluent, to give the target products.

3-(4-(benzyloxy)phenyl)pentanal (3a)

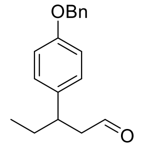

White solid. 49.4 mg, 92% yield. **^{1}H NMR** (400 MHz, $CDCl_3$): δ 9.67 (t, J=2.0 Hz, 1H), 7.46-7.38 (m, 4H), 7.36-7.32 (m, 1H), 7.12 (dt, J_1=8.4 Hz, J_2=2.4 Hz, 2H), 6.94 (dt, J_1=8.8 Hz, J_2=2.4 Hz, 2H), 5.05 (s, 2H), 3.09-3.01 (m, 1H), 2.69 (dd, J_1=7.6 Hz, J_2=2.0 Hz, 2H), 1.76-1.55 (m, 2H), 0.82 (t, J=7.2 Hz, 3H). **^{13}C NMR** (101 MHz, $CDCl_3$): δ 202.39, 157.59, 137.20, 136.05, 128.69, 128.57, 128.06, 127.62, 114.99, 70.13, 50.47, 41.11, 29.74, 11.99. **MS** (ESI): m/z for $C_{18}H_{20}O_2$ $[M]^+$ calcd 268.15, found 268.17.

3-([1,1'-biphenyl]-4-yl)pentanal (3b)

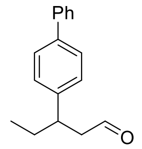

White solid. 43.9 mg, 92% yield. **^{1}H NMR** (400 MHz, $CDCl_3$): δ 9.71 (t, J=2.0 Hz, 1H), 7.60-7.54 (m, 4H), 9.44 (t, J=8.0 Hz, 2H), 7.36-7.32 (m, 1H), 7.27-7.25 (m, 2H), 3.18-3.11 (m, 1H), 2.76 (dd, J_1=7.6 Hz, J_2=1.6 Hz, 2H), 1.81-1.62 (m, 2H), 0.86 (t, J=7.2 Hz, 3H). **^{13}C NMR** (101 MHz, $CDCl_3$): δ 202.16, 142.90, 140.98, 139.62, 128.88, 128.09, 127.44, 127.29, 127.13, 50.33, 41.54, 29.64, 12.07. **MS** (ESI): m/z for $C_{17}H_{18}O$ $[M]^+$ calcd 238.14, found 238.17.

3-(*o*-tolyl)pentanal (3c)

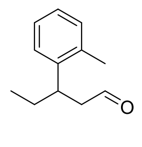

Colorless oil. 33.1 mg, 94% yield. **^{1}H NMR** (400 MHz, $CDCl_3$): δ 9.66 (t, J=2.0 Hz, 1H), 7.20-7.08 (m, 4H), 3.45-3.37 (m, 1H), 2.72 (dd, J_1=7.6 Hz, J_2=2.0 Hz, 2H), 2.37 (s, 3H), 1.74-1.60 (m, 2H), 0.82 (t, J=7.2 Hz, 3H). **^{13}C NMR** (101 MHz, $CDCl_3$): δ 202.26, 142.14, 136.13, 130.61, 126.52, 126.26, 125.86, 50.25, 36.20, 29.49, 20.02, 11.85. **MS** (ESI): m/z for $C_{12}H_{16}O$ $[M]^+$ calcd 176.12, found 176.14.

3-(*m*-tolyl)pentanal (3d)

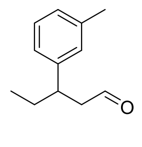

Colorless oil. 32.4 mg, 92% yield. **^{1}H NMR** (400 MHz, $CDCl_3$): δ 9.66 (t, J=2.0 Hz, 1H), 7.19 (t, J=8.0 Hz, 1H), 7.03-6.97 (m, 3H), 3.07-3.00 (m, 1H), 2.71 (dd, J_1=7.6 Hz, J_2=2.0

Hz, 2H), 2.33 (s, 3H), 1.73-1.58 (m, 2H), 0.81 (t, *J*=7.2 Hz, 3H). **^{13}C NMR** (101 MHz, $CDCl_3$): δ 202.47, 143.75, 138.30, 128.61, 128.45, 127.48, 124.67, 50.36, 41.90, 29.64, 21.63, 12.07. **MS** (ESI): m/z for $C_{12}H_{16}O$ $[M]^+$ calcd 176.12, found 176.14.

3-(4-heptylphenyl)pentanal (3e)

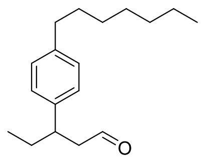

Colorless oil. 33.1 mg, 93% yield. **^{1}H NMR** (400 MHz, $CDCl_3$): δ 9.66 (t, *J*=2.0 Hz, 1H), 7.12-7.07 (m, 4H), 3.09-3.01 (m, 1H), 2.70 (dd, J_1=7.2 Hz, J_2=2.0 Hz, 2H), 2.56 (t, *J*=7.6 Hz, 2H), 1.73-1.56 (m, 4H), 1.32-1.27 (m, 8H), 0.88 (t, *J*=6.8 Hz, 3H), 0.81 (t, *J*=7.2 Hz, 3H). **^{13}C NMR** (101 MHz, $CDCl_3$): δ 202.55, 141.33, 140.88, 128.71, 127.48, 50.37, 41.57, 35.70, 31.96, 31.60, 29.70, 29.49, 29.33, 22.81, 14.24, 12.05. **MS** (ESI): m/z for $C_{18}H_{28}O$ $[M]^+$ calcd 260.21, found 260.20.

3-(4-(*tert*-butyl)phenyl)pentanal (3f)

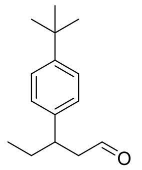

Colorless oil. 35.8 mg, 82% yield. **^{1}H NMR** (400 MHz, $CDCl_3$): δ 9.67 (t, *J*=2.0 Hz, 1H), 7.31 (dt, J_1=8.8 Hz, J_2=2.4 Hz, 2H), 7.11 (dt, J_1=8.4 Hz, J_2=2.4 Hz, 2H), 3.10-3.02 (m, 1H), 2.70 (dd, J_1=7.2 Hz, J_2=2.0 Hz, 2H), 1.75-1.60 (m, 2H), 1.30 (s, 9H), 0.82 (t, *J*=7.2 Hz, 3H). **^{13}C NMR** (101 MHz, $CDCl_3$): δ 202.63, 149.44, 140.64, 127.25, 125.57, 50.28, 41.38, 34.52, 31.51, 29.66, 12.09. **MS** (ESI): m/z for $C_{15}H_{22}O$ $[M]^+$ calcd 218.17, found 218.19.

3-(3-methoxyphenyl)pentanal (3g)

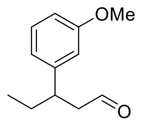

Colorless oil. 33.4 mg, 87% yield. **^{1}H NMR** (400 MHz, $CDCl_3$): δ 9.67 (t, *J*=2.0 Hz, 1H), 7.22 (t, *J*=8.0 Hz, 2H), 6.79-6.72 (m, 3H), 3.80 (s, 3H), 3.09-3.02 (m, 1H), 2.70 (dd, J_1=7.2 Hz, J_2=2.0 Hz, 2H), 1.73-1.59 (m, 2H), 0.81 (t, *J*=7.2 Hz, 3H). **^{13}C NMR** (101 MHz, $CDCl_3$): δ 202.24, 159.88, 145.49, 129.71, 120.03, 113.73, 111.58, 55.28, 50.28, 41.94, 29.55, 12.03. **MS** (ESI): m/z for $C_{12}H_{16}O_2$ $[M]^+$ calcd 192.12, found 192.14.

3-(4-fluorophenyl)pentanal (3h)

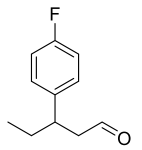

Colorless oil. 32.4 mg, 90% yield. **^{1}H NMR** (400 MHz, $CDCl_3$): δ 9.66 (t, *J*=2.0 Hz, 1H), 7.16-7.12 (m, 2H), 6.99 (t, *J*=8.4 Hz, 2H), 3.12-3.04 (m, 1H), 2.71-2.69 (m, 2H), 1.75-1.55 (m, 2H), 0.80 (t, *J*=7.2 Hz, 3H). **^{13}C NMR** (101 MHz, $CDCl_3$): δ 201.85, 161.66 (d, *J*=243 Hz), 139.45 (d, *J*=3 Hz), 129.04 (d, *J*=8 Hz), 115.54 (d, *J*=21 Hz), 50.50, 41.11, 29.73, 11.96. **MS** (ESI): m/z for $C_{11}H_{13}FO$ $[M]^+$ calcd 180.10, found 180.13.

3-(4-chlorophenyl)pentanal (3i)

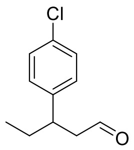

Colorless oil. 24.3 mg, 62% yield. **^{1}H NMR** (400 MHz, $CDCl_3$): δ 9.66 (t, J=2.0 Hz, 1H), 7.28 (dt, J_1=8.8 Hz, J_2=2.4 Hz, 2H), 7.11 (dt, J_1=8.4 Hz, J_2=2.0 Hz, 2H), 3.11-3.04 (m, 1H), 2.71 (dt, J_1=7.2 Hz, J_2=2.0 Hz, 2H), 1.75-1.65 (m, 1H), 1.62-1.55 (m, 1H), 0.80 (t, J=7.2 Hz, 3H). **^{13}C NMR** (101 MHz, $CDCl_3$): δ 201.62, 142.32, 132.37, 129.03, 128.88, 50.32, 41.20, 29.55, 11.95. **MS** (ESI): m/z for $C_{11}H_{13}ClO$ $[M]^+$ calcd 196.07 (198.06), found 196.10 (198.10).

3-(4-bromophenyl)pentanal (3j)

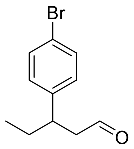

Colorless oil. 37.1 mg, 77% yield. **^{1}H NMR** (400 MHz, $CDCl_3$): δ 9.66 (t, J=2.0 Hz, 1H), 7.42 (dt, J_1=8.4 Hz, J_2=2.0 Hz, 2H), 7.05 (dt, J_1=8.4 Hz, J_2=2.4 Hz, 2H), 3.10-3.02 (m, 1H), 2.71 (dt, J_1=7.6 Hz, J_2=2.0 Hz, 2H), 1.75-1.65 (m, 1H), 1.62-1.55 (m, 1H), 0.79 (t, J=7.2 Hz, 3H). **^{13}C NMR** (101 MHz, $CDCl_3$): δ 201.56, 142.86, 131.83, 129.43, 120.41, 50.26, 41.25, 29.49, 11.95. **MS** (ESI): m/z for $C_{11}H_{13}BrO$ $[M]^+$ calcd 240.01 (242.01), found 240.04 (242.04).

3-(2-fluorophenyl)pentanal (3k)

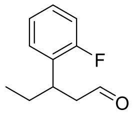

Colorless oil. 33.8 mg, 94% yield. **^{1}H NMR** (400 MHz, $CDCl_3$): δ 9.69 (t, J=2.0 Hz, 1H), 7.22-7.16 (m, 2H), 7.11-7.07 (m, 1H), 7.04-7.00 (m, 1H), 3.46-3.39 (m, 1H), 2.76 (dd, J_1=7.2 Hz, J_2=2.0 Hz, 2H), 1.79-1.64 (m, 2H), 0.83 (t, J=7.2 Hz, 3H). **^{13}C NMR** (101 MHz, $CDCl_3$): δ 201.83, 161.11 (d, J=244 Hz), 130.34 (d, J=14 Hz), 129.02 (d, J=5 Hz), 128.18 (d, J=8 Hz), 124.40 (d, J=4 Hz), 115.78 (d, J=23 Hz), 49.14, 35.27, 28.30, 11.99. **MS** (ESI): m/z for $C_{11}H_{13}FO$ $[M]^+$ calcd 180.10, found 180.11.

3-(3-chlorophenyl)pentanal (3l)

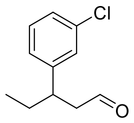

Colorless oil. 27.4 mg, 70% yield. **^{1}H NMR** (400 MHz, $CDCl_3$): δ 9.68 (t, J=2.0 Hz, 1H), 7.24-7.17 (m, 3H), 7.07 (t, J=7.2 Hz, 1H), 3.11-3.04 (m, 1H), 2.72 (dd, J_1=7.2 Hz, J_2=1.6 Hz, 2H), 1.76-1.57 (m, 2H), 0.81 (t, J=7.2 Hz, 3H). **^{13}C NMR** (101 MHz, $CDCl_3$): δ 201.46, 146.03, 134.57, 130.01, 127.76, 126.96, 126.02, 50.22, 41.52, 29.48, 11.99. **MS** (ESI): m/z for $C_{11}H_{13}ClO$ $[M]^+$ calcd 196.07 (198.06), found 196.09 (198.09).

3-(3-bromophenyl)pentanal (3m)

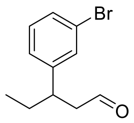

Colorless oil. 39.8 mg, 83% yield. **^{1}H NMR** (400 MHz, $CDCl_3$): δ 9.68 (t, J=2.0 Hz, 1H), 7.34 (d, J=7.6 Hz, 2H), 7.18 (t, J=7.6 Hz, 1H), 7.11 (d, J=7.6 Hz, 1H), 3.10-3.03 (m, 1H), 2.72 (dd, J_1=7.2 Hz, J_2=1.6 Hz, 2H), 1.76-1.58 (m, 2H), 0.81 (t, J=7.2 Hz, 3H). **^{13}C NMR** (101 MHz, $CDCl_3$): δ 201.43, 146.34, 130.67, 130.32, 129.88, 126.49, 122.86, 50.22, 41.49, 29.48, 11.99. **MS** (ESI): m/z for $C_{11}H_{13}ClO$ $[M]^+$ calcd 240.01 (242.01), found 240.05 (242.05).

4-(1-oxopentan-3-yl)benzonitrile (3n)

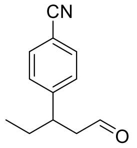

Colorless oil. 20.9 mg, 56% yield. ^{1}**H NMR** (400 MHz, $CDCl_3$): δ 9.68 (t, *J*=1.6 Hz, 1H), 7.60 (d, *J*=8.4 Hz, 2H), 7.30 (d, *J*=8.4 Hz, 2H), 3.21-3.14 (m, 1H), 2.83-2.70 (m, 2H), 1.79-1.56 (m, 2H), 0.80 (t, *J*=7.2 Hz, 3H). ^{13}**C NMR** (101 MHz, $CDCl_3$): δ 200.66, 149.66, 132.60, 128.57, 118.98, 110.68, 49.99, 41.65, 29.29, 11.93. **MS** (ESI): m/z for $C_{12}H_{13}NO$ $[M]^+$ calcd 187.10, found 187.11.

N-(4-(1-oxopentan-3-yl)phenyl)acetamide (3o)

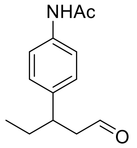

White solid. 37.2 mg, 85% yield. ^{1}**H NMR** (400 MHz, $CDCl_3$): δ 9.65 (t, *J*=1.6 Hz, 1H), 7.42 (d, *J*=8.4 Hz, 2H), 7.32 (s, 1H), 7.13 (d, *J*=8.4 Hz, 2H), 3.08-3.01 (m, 1H), 2.69 (dd, J_1=7.2 Hz, J_2=2.0 Hz, 2H), 2.15 (s, 3H), 1.74-1.55 (m, 2H), 0.79 (t, *J*=7.2 Hz, 3H). ^{13}**C NMR** (101 MHz, $CDCl_3$): δ 202.24, 168.44, 139.75, 136.47, 128.17, 120.30, 50.36, 41.35, 29.62, 24.67, 11.97. **MS** (ESI): m/z for $C_{13}H_{17}NO_2$ $[M]^+$ calcd 219.13, found 219.12.

3-(naphthalen-1-yl)pentanal (3p)

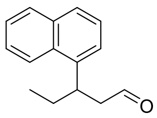

Colorless oil. 38.6 mg, 91% yield. ^{1}**H NMR** (400 MHz, $CDCl_3$): δ 9.72 (t, *J*=2.0 Hz, 1H), 8.17 (d, *J*=8.4 Hz, 1H), 7.88 (d, *J*=7.6 Hz, 1H), 7.75 (d, *J*=8.0 Hz, 1H), 7.57-7.44 (m, 3H), 7.36 (d, *J*=7.2 Hz, 1H), 4.12-4.05 (m, 1H), 2.88 (dd, J_1=7.2 Hz, J_2=2.0 Hz, 2H), 1.91-1.84 (m, 2H), 0.87 (t, *J*=7.6 Hz, 3H). ^{13}**C NMR** (101 MHz, $CDCl_3$): δ 202.20, 139.87, 134.18, 131.94, 129.22, 127.18, 126.23, 125.69, 125.62, 123.61, 123.06, 49.87, 29.15, 11.94. **MS** (ESI): m/z for $C_{15}H_{16}O$ $[M]^+$ calcd 212.12, found 212.16.

3-(naphthalen-2-yl)pentanal (3q)

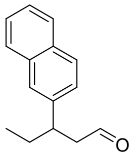

White solid. 30.5 mg, 72% yield. ^{1}**H NMR** (400 MHz, $CDCl_3$): δ 9.70 (t, *J*=2.0 Hz, 1H), 7.82-7.78 (m, 3H), 7.63 (s, 1H), 7.49-7.42 (m, 2H), 7.34 (dd, J_1=8.4 Hz, J_2=1.6 Hz, 1H), 3.30-3.23 (m, 1H), 2.81 (dd, J_1=7.6 Hz, J_2=2.0 Hz, 2H), 1.84-1.70 (m, 2H), 0.84 (t, *J*=7.6 Hz, 3H). ^{13}**C NMR** (101 MHz, $CDCl_3$): δ 202.15, 141.19, 133.65, 132.57, 128.56, 127.76, 126.41, 126.25, 125.77, 125.67, 50.35, 42.06, 29.55, 12.08. **MS** (ESI): m/z for $C_{15}H_{16}O$ $[M]^+$ calcd 212.12, found 212.17.

3-(thiophen-3-yl)pentanal (3r)

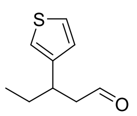

Colorless oil. 25.2 mg, 75% yield. ^{1}**H NMR** (400 MHz, $CDCl_3$): δ 9.68 (t, *J*=2.0 Hz, 1H), 7.28 (dd, J_1=4.8 Hz, J_2=3.2 Hz, 1H), 6.97 (d, *J*=3.2 Hz, 1H), 6.95 (d, *J*=4.8 Hz, 1H), 3.29-

3.22 (m, 1H), 2.68 (dd, J_1=7.2 Hz, J_2=2.0 Hz, 2H), 1.73-1.60 (m, 2H), 0.84 (t, J=7.6 Hz, 3H). **^{13}C NMR** (101 MHz, $CDCl_3$): δ 202.26, 144.62, 126.66, 126.07, 120.65, 49.89, 37.06, 29.38, 11.88. **MS** (ESI): m/z for $C_9H_{12}OS$ $[M]^+$ calcd 168.06, found 168.08.

3-(4-methoxyphenyl)octanal (3s)

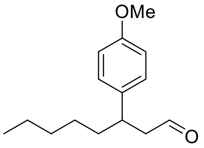

Colorless oil. 28.1 mg, 60% yield. **^{1}H NMR** (400 MHz, $CDCl_3$): δ 9.65 (t, J=2.0 Hz, 1H), 7.10 (dt, J_1=8.8 Hz, J_2=2.4 Hz, 2H), 6.84 (dt, J_1=8.4 Hz, J_2=2.4 Hz, 2H), 3.79 (s, 3H), 3.15-3.07 (m, 1H), 2.67 (dd, J_1=7.2 Hz, J_2=2.0 Hz, 2H), 1.65-1.54 (m, 2H), 1.25-1.13 (m, 6H), 0.83 (t, J=7.6 Hz, 3H). **^{13}C NMR** (101 MHz, $CDCl_3$): δ 202.50, 158.31, 136.10, 128.49, 114.12, 55.37, 50.95, 39.49, 36.89, 31.83, 27.09, 22.64, 14.16. **MS** (ESI): m/z for $C_{15}H_{22}O_2$ $[M]^+$ calcd 234.16, found 234.20.

6-((*tert*-butyldimethylsilyl)oxy)-3-(4-methoxyphenyl)hexanal (3t)

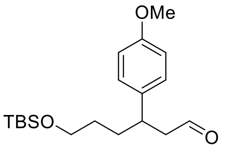

Colorless oil. 57.2 mg, 85% yield. **^{1}H NMR** (400 MHz, $CDCl_3$): δ 9.65 (t, J=2.0 Hz, 1H), 7.10 (dt, J_1=8.8 Hz, J_2=1.6 Hz, 2H), 6.84 (dt, J_1=8.4 Hz, J_2=2.0 Hz, 2H), 3.78 (s, 3H), 3.54 (t, J=6.4 Hz, 2H), 3.17-3.09 (m, 1H), 2.68 (dd, J_1=7.2 Hz, J_2=2.4 Hz, 2H), 1.76-1.67 (m, 1H), 1.65-1.59 (m, 1H), 1.45-1.31 (m, 6H), 0.87 (s, 9H), 0.01 (s, 3H), 0.00 (s, 3H),. **^{13}C NMR** (101 MHz, $CDCl_3$): δ 202.32, 158.38, 135.78, 128.54, 114.18, 63.00, 55.39, 51.01, 39.28, 33.08, 30.63, 26.09, 18.47, -5.17. **MS** (ESI): m/z for $C_{19}H_{32}O_3Si$ $[M\text{-}^tBu]^+$ calcd 279.14, found 279.20.

1-cyclohexyl-3-(4-methoxyphenyl)pentan-1-one (3u)

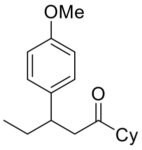

Colorless oil. 39.5 mg, 72% yield. **^{1}H NMR** (400 MHz, $CDCl_3$): δ 7.08 (dt, J_1=8.4 Hz, J_2=2.4 Hz, 2H), 6.81 (dt, J_1=8.8 Hz, J_2=2.4 Hz, 2H), 3.78 (s, 3H), 3.04-2.97 (m, 1H), 2.68 (d, J=7.2 Hz, 2H), 2.21-2.15 (m, 1H), 1.74-1.59 (m, 6H), 1.54-1.46 (m, 1H), 1.26-1.15 (m, 5H), 0.75 (t, J=7.6 Hz, 3H). **^{13}C NMR** (101 MHz, $CDCl_3$): δ 213.37, 158.05, 136.92, 128.60, 113.84, 55.35, 51.43, 48.12, 41.92, 29.45, 28.37, 28.21, 25.98, 25.79, 25.74, 12.19. **MS** (ESI): m/z for $C_{18}H_{26}O_2$ $[M]^+$ calcd 274.19, found 274.23.

4-(4-methoxyphenyl)hexanal (3v)

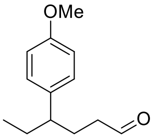

Colorless oil. 21.4 mg, 52% yield. **^{1}H NMR** (400 MHz, $CDCl_3$): δ 9.65 (t, J=1.6 Hz, 1H), 7.02 (dt, J_1=8.8 Hz, J_2=2.4 Hz, 2H), 6.84 (dt, J_1=8.8 Hz, J_2=2.4 Hz, 2H), 3.79 (s, 3H), 2.41-2.33 (m, 1H), 2.29-2.24 (m, 2H), 2.05-1.96 (m, 1H), 1.82-1.62 (m, 2H), 1.57-1.50

(m, 1H), 0.78 (t, *J*=7.6 Hz, 3H). **^{13}C NMR** (101 MHz, $CDCl_3$): *δ* 202.79, 158.21, 136.38, 128.71, 113.99, 55.36, 46.49, 42.39, 29.98, 28.90, 12.27. **MS** (ESI): m/z for $C_{13}H_{18}O_2$ $[M]^+$ calcd 206.13, found 206.16.

5-(4-methoxyphenyl)heptanal (3w)

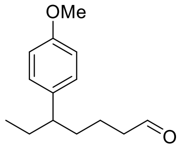

Colorless oil. 24.6 mg, 56% yield. **^{1}H NMR** (400 MHz, $CDCl_3$): *δ* 9.68 (t, *J*=1.6 Hz, 1H), 7.04 (dt, J_1=8.8 Hz, J_2=2.4 Hz, 2H), 6.83 (dt, J_1=8.8 Hz, J_2=2.4 Hz, 2H), 3.79 (s, 3H), 2.39-2.32 (m, 3H), 1.70-1.61 (m, 2H), 1.54-1.42 (m, 4H), 0.75 (t, *J*=7.6 Hz, 3H). **^{13}C NMR** (101 MHz, $CDCl_3$): *δ* 202.99, 157.98, 137.26, 128.63, 113.83, 55.35, 47.00, 44.06, 36.19, 29.97, 20.40, 12.27. **MS** (ESI): m/z for $C_{14}H_{20}O_2$ $[M]^+$ calcd 220.15, found 220.19.

4-(4-(trifluoromethyl)phenyl)hexanal (3x)

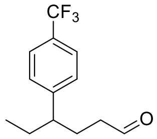

Colorless oil. 24.9 mg, 51% yield. **^{1}H NMR** (400 MHz, $CDCl_3$): *δ* 9.66 (s, 1H), 7.56 (d, *J*=8.0 Hz, 2H), 7.24 (d, *J*=8.0 Hz, 2H), 2.55-2.48 (m, 1H), 2.33-2.22 (m, 2H), 2.11-2.03 (m, 1H), 1.88-1.68 (m, 2H), 1.64-1.57 (m, 1H), 0.78 (t, *J*=7.6 Hz, 3H). **^{13}C NMR** (101 MHz, $CDCl_3$): *δ* 201.18, 148.07, 129.06 (q, *J*=33 Hz), 128.06, 125.70 (q, *J*=3 Hz), 124.33 (q, *J*=271 Hz), 50.16, 41.52, 29.85, 29.43, 11.94. **MS** (ESI): m/z for $C_{13}H_{15}F_3O$ $[M]^+$ calcd 244.11, found 244.12.

4-(4-(benzyloxy)phenyl)hexanal (3y)

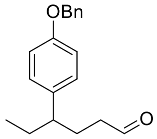

Colorless oil. 46.8 mg, 83% yield. **^{1}H NMR** (400 MHz, $CDCl_3$): *δ* 9.66 (t, *J*=1.2 Hz, 1H), 7.45-7.37 (m, 4H), 7.35-7.31 (m, 1H), 7.03 (dt, J_1=8.8 Hz, J_2=2.4 Hz, 2H), 6.92 (dt, J_1=8.8 Hz, J_2=2.4 Hz, 2H), 5.04 (s, 2H), 2.41-2.34 (m, 1H), 2.30-2.25 (m, 2H), 2.05-1.97 (m, 1H), 1.82-1.62 (m, 2H), 1.60-1.52 (m, 1H), 0.78 (t, *J*=7.6 Hz, 3H). **^{13}C NMR** (101 MHz, $CDCl_3$): *δ* 202.78, 157.50, 137.29, 136.70, 128.73, 128.72, 128.09, 127.69, 114.91, 70.18, 46.50, 42.38, 29.96, 28.88, 12.28. **MS** (ESI): m/z for $C_{19}H_{22}O_2$ $[M]^+$ calcd 282.16, found 282.14.

4-([1,1'-biphenyl]-4-yl)hexanal (3z)

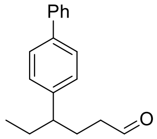

Colorless oil. 39.3 mg, 78% yield. **^{1}H NMR** (400 MHz, $CDCl_3$): *δ* 9.69 (t, *J*=1.2 Hz, 1H), 7.59 (d, *J*=7.2 Hz, 2H), 7.54 (d, *J*=8.0 Hz, 2H), 7.43 (t, *J*=7.6 Hz, 2H), 7.33 (t, *J*=7.2 Hz, 1H), 7.19 (d, *J*=8.4 Hz, 2H), 2.52-2.44 (m, 1H), 2.37-2.30 (m, 2H), 2.11-2.03 (m, 1H), 1.90-1.81 (m, 1H), 1.77-1.60 (m, 2H), 0.83 (t, *J*=7.6 Hz, 3H). **^{13}C NMR** (101 MHz, $CDCl_3$): *δ* 202.68, 143.57, 141.07, 139.36, 128.88, 128.27, 127.32, 127.23, 127.11, 46.95, 42.37, 29.85, 28.66, 12.32. **MS** (ESI): m/z for $C_{18}H_{20}O$ $[M]^+$ calcd 252.15, found 252.15.

4-(3-fluorophenyl)hexanal (3aa)

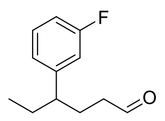

Colorless oil. 29.9 mg, 77% yield. **^{1}H NMR** (400 MHz, $CDCl_3$): δ 9.68 (t, *J*=1.2 Hz, 1H), 7.28-7.23 (m, 1H), 6.92-6.87 (m, 2H), 6.85-6.81 (m, 1H), 2.48-2.40 (m, 1H), 2.33-2.22 (m, 2H), 2.08-1.99 (m, 1H), 1.84-1.56 (m, 3H), 0.79 (t, *J*=7.6 Hz, 3H). **^{13}C NMR** (101 MHz, $CDCl_3$): δ 202.33, 163.22 (d, *J*=244 Hz), 147.26 (d, *J*=7 Hz), 130.04 (d, *J*=8 Hz), 123.65 (d, *J*=2 Hz), 114.44 (d, *J*=20 Hz), 113.38 (d, *J*=21 Hz), 47.10, 42.19, 29.74, 28.57, 12.18. **MS** (ESI): m/z for $C_{12}H_{15}FO$ $[M]^+$ calcd 194.11, found 194.10.

4-(2-fluorophenyl)hexanal (3ab)

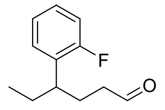

Colorless oil. 23.7 mg, 61% yield. **^{1}H NMR** (400 MHz, $CDCl_3$): δ 9.68 (t, *J*=1.2 Hz, 1H), 7.20-7.08 (m, 3H), 7.03-6.99 (m, 1H), 2.90-2.82 (m, 1H), 2.39-2.23 (m, 2H), 2.10-2.01 (m, 1H), 1.91-1.81 (m, 1H), 1.76-1.61 (m, 2H), 0.81 (t, *J*=7.6 Hz, 3H). **^{13}C NMR** (101 MHz, $CDCl_3$): δ 202.42, 161.50 (d, *J*=242 Hz), 130.95 (d, *J*=15 Hz), 128.63 (d, *J*=5 Hz), 127.81 (d, *J*=9 Hz), 124.41 (d, *J*=3 Hz), 115.55 (d, *J*=23 Hz), 42.25, 39.75, 28.60, 27.65, 12.17. **MS** (ESI): m/z for $C_{12}H_{15}FO$ $[M]^+$ calcd 194.11, found 194.12.

4-(*o*-tolyl)hexanal (3ac)

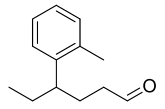

Colorless oil. 25.5 mg, 67% yield. **^{1}H NMR** (400 MHz, $CDCl_3$): δ 9.66 (t, *J*=1.6 Hz, 1H), 7.20-7.07 (m, 4H), 2.87-2.79 (m, 1H), 2.29-2.26 (m, 5H), 2.08-1.99 (m, 1H), 1.90-1.80 (m, 1H), 1.73-1.56 (m, 2H), 0.80 (t, *J*=7.6 Hz, 3H). **^{13}C NMR** (101 MHz, $CDCl_3$): δ 202.70, 142.75, 136.60, 130.40, 126.49, 125.93, 125.68, 42.14, 41.05, 29.82, 28.40, 20.12, 12.06. **MS** (ESI): m/z for $C_{13}H_{18}O$ $[M]^+$ calcd 190.14, found 190.15.

4-(naphthalen-1-yl)hexanal (3ad)

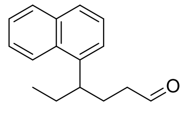

Colorless oil. 18.1 mg, 40% yield. **^{1}H NMR** (400 MHz, $CDCl_3$): δ 9.64 (s, 1H), 8.10 (d, *J*=8.4 Hz, 1H), 7.87 (d, *J*=7.2 Hz, 1H), 7.73 (d, *J*=8.0 Hz, 1H), 7.53-7.45 (m, 3H), 7.36 (d, *J*=7.2 Hz, 1H), 3.49 (s, 1H), 2.34-2.27 (m, 2H), 2.24-2.16 (m, 1H), 2.09-1.99 (m, 1H), 1.90-1.77 (m, 2H), 0.84 (t, *J*=7.6 Hz, 3H). **^{13}C NMR** (101 MHz, $CDCl_3$): δ 202.58, 140.76, 134.10, 132.79, 129.17, 126.80, 125.99, 125.71, 125.51, 123.16, 42.12, 39.52, 29.61, 28.17, 12.17. **MS** (ESI): m/z for $C_{16}H_{18}O$ $[M]^+$ calcd 226.14, found 226.18.

4. Substrate scope on styrene-derived alkenols

General Procedure: **1** (0.2 mmol, 1 eq), **2** (0.4 mmol, 2 eq), $Pd(OAc)_2$ (200 ppm), tetrabutylammonium bromide (0.2 mmol, 1 eq) and sodium formate (0.4 mmol, 2 eq) was added to a Schlenk tube equipped with a magnetic stirrer. The tube was degassed and refilled with nitrogen for three times. Toluene (2 mL) was introduced and the tube was sealed. The solution was allowed to stir for 30 min at rt and then heated to 125 °C and stirred for another 24 h. After reaction was completed, cooled to rt and filtered. The resulting solution was concentrated and purified through column chromatography with hexane/EtOAc as eluent, to give the target products.

3-(4-methoxyphenyl)-3-phenylpropanal (4a)

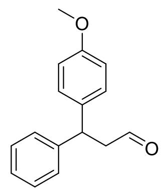

Colorless oil. 36.5 mg, 76% yield. **^{1}H NMR** (400 MHz, $CDCl_3$): δ 9.73 (t, *J*=2.0 Hz, 1H), 7.34-7.16 (m, 7H), 6.88-6.84 (m, 2H), 4.63-4.57 (m, 1H), 3.77 (s, 3H), 3.16-3.13 (m, 2H). **^{13}C NMR** (101 MHz, $CDCl_3$): δ 201.35, 158.33, 143.70, 135.38, 128.78, 127.69, 126.68, 114.15, 55.28, 49.61, 44.24. **MS** (ESI): m/z for $C_{16}H_{16}O_2$ $[M]^+$ calcd 240.12, found 240.15.

3-([1,1'-biphenyl]-4-yl)-3-phenylpropanal (4b)

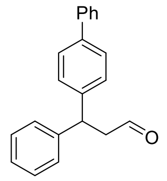

Colorless oil. 43 mg, 75% yield. **^{1}H NMR** (400 MHz, $CDCl_3$): δ 9.78 (t, *J*=2.0 Hz, 1H), 7.58-7.53 (m, 4H), 7.46-7.42 (m, 2H), 7.37-7.22 (m, 8H), 4.69 (t, *J*=8.0 Hz, 1H), 3.22 (dd, J_1=8.0 Hz, J_2=2.0 Hz, 2H). **^{13}C NMR** (101 MHz, $CDCl_3$): δ 201.13, 143.26, 142.40, 140.77, 139.72, 128.92, 128.88, 128.24, 127.86, 127.57, 127.37, 127.13, 126.90, 49.50, 44.72. **MS** (ESI): m/z for $C_{21}H_{18}O$ $[M]^+$ calcd 286.14, found 286.15.

3-(3-chlorophenyl)-3-phenylpropanal (4c)

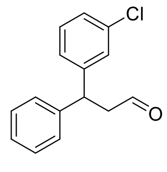

Colorless oil. 26 mg, 53% yield. **^{1}H NMR** (400 MHz, $CDCl_3$): δ 9.74 (t, *J*=2.0 Hz, 1H), 7.33-7.29 (m, 2H), 7.25-7.17 (m, 6H), 7.12 (dt, J_1=7.2 Hz, J_2=1.6 Hz, 1H), 4.60 (t, *J*=8.0 Hz, 1H), 3.17 (dd, J_1=7.6 Hz, J_2=1.6 Hz, 2H). **^{13}C NMR** (101 MHz, $CDCl_3$): δ 200.50, 145.47, 142.56, 134.66, 130.12, 129.02, 128.02, 127.81, 127.13, 127.07, 126.08, 49.33, 44.63. **MS** (ESI): m/z for $C_{15}H_{13}ClO$ $[M]^+$ calcd 244.07 (246.07), found 244.10 (246.10).

3-(2-fluorophenyl)-3-phenylpropanal (4d)

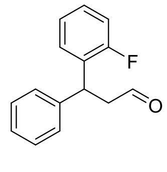

Colorless oil. 26.9 mg, 59% yield. **^{1}H NMR** (400 MHz, $CDCl_3$): δ 9.74 (t, *J*=2.0 Hz, 1H), 7.33-7.18 (m, 7H), 7.10-7.00 (m, 2H), 4.93 (t, *J*=8.0 Hz, 1H), 3.19 (d, *J*=7.6 Hz, 2H).

^{13}C NMR (101 MHz, $CDCl_3$): δ 200.79, 160.56 (d, J=245 Hz), 141.99, 130.31 (d, J=14 Hz), 129.27, 128.92, 128.88, 128.57 (d, J=9 Hz), 127.88, 127.02, 124.49 (d, J=4 Hz), 115.95 (d, J=22 Hz), 48.42, 38.35. **MS** (ESI): m/z for $C_{15}H_{13}FO$ $[M]^+$ calcd 228.10, found 228.14.

3,3-bis(4-bromophenyl)propanal (4e)

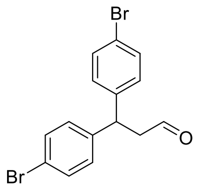

Colorless oil. 15.5 mg, 21% yield. **^{1}H NMR** (400 MHz, $CDCl_3$): δ 9.73 (q, J=1.2 Hz, 1H), 7.42 (d, J=8.4 Hz, 4H), 7.07 (d, J=8.0 Hz, 4H), 4.56 (t, J=7.6 Hz, 1H), 3.15 (dt, J_1=7.6 Hz, J_2=1.2 Hz, 2H). **^{13}C NMR** (101 MHz, $CDCl_3$): δ 199.98, 141.86, 132.07, 129.51, 120.96, 49.26, 43.71. **MS** (ESI): m/z for $C_{15}H_{12}Br_2O$ $[M]^+$ calcd 365.93 (367.92, 369.92), found 365.91 (365.90, 365.90).

3-(4-bromophenyl)-3-(3-methoxyphenyl)propanal (4f)

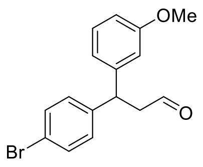

Colorless oil. 45.1 mg, 71% yield. **^{1}H NMR** (400 MHz, $CDCl_3$): δ 9.73 (q, J=1.6 Hz, 1H), 7.41 (d, J=7.6 Hz, 2H), 7.22 (t, J=8.0 Hz, 1H), 7.10 (d, J=8.0 Hz, 2H), 6.80-6.73 (m, 3H), 4.55 (t, J=7.6 Hz, 1H), 3.77 (s, 3H), 3.15-3.12 (m, 2H). **^{13}C NMR** (101 MHz, $CDCl_3$): δ 200.56, 160.00, 144.36, 142.30, 131.94, 130.01, 129.57, 120.74, 120.06, 114.11, 111.81, 55.33, 49.31, 44.38. **MS** (ESI): m/z for $C_{16}H_{15}BrO_2$ $[M]^+$ calcd 318.03 (320.02), found 318.06 (320.06).

3-(4-(benzyloxy)phenyl)-3-(4-bromophenyl)propanal (4g)

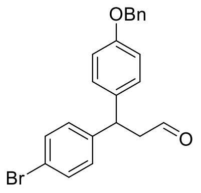

Colorless oil. 28.4 mg, 36% yield. **^{1}H NMR** (400 MHz, $CDCl_3$): δ 9.73 (q, J=2.0 Hz, 1H), 7.43-7.33 (m, 7H), 7.11 (d, J_1=8.4 Hz, J_2=1.6 Hz, 4H), 6.93-6.90 (m, 2H), 5.03 (s, 2H), 4.55 (t, J=8.0 Hz, 1H), 3.11 (dt, J_1=7.6 Hz, J_2=2.0 Hz, 2H). **^{13}C NMR** (101 MHz, $CDCl_3$): δ 200.76, 157.78, 142.79, 137.00, 135.09, 131.90, 129.51, 128.78, 128.74, 128.15, 127.61, 120.59, 115.21, 70.14, 49.53, 43.63. **MS** (ESI): m/z for $C_{22}H_{19}BrO_2$ $[M]^+$ calcd 394.06 (396.05), found 394.08 (396.08).

5. Mechanism studies

1. *Hot filtration test*:

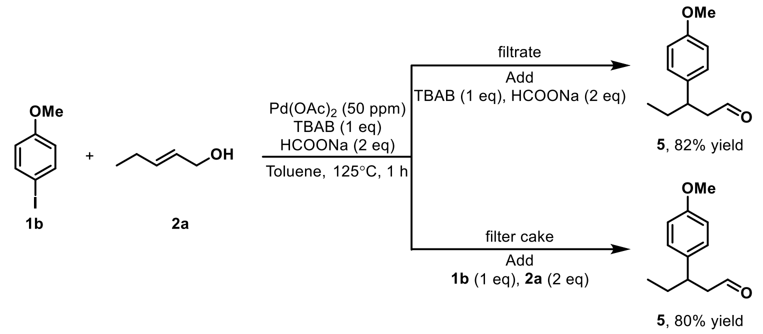

Hot filtration test has been conducted as described: After the standard reaction (0.2 mmol scale) proceeded for 1h, filtration was performed, and the resulting filter cake and filtrate were transferred into separate reaction flasks. Both the filtrate and the filter cake were concentrated to remove all volatiles. Following this, **1b** (1 eq) and **2a** (2 eq) were added to the filter cake. TBAB (1 eq) and HCOONa (2 eq) were added to the filtrate. These two reactions were then allowed to continue for an additional 24 hours at 125 ˚C. After reaction was completed, cooled to rt and filtered. The resulting solution was concentrated and purified through column chromatography with hexane/EtOAc as eluent, to give the target products.

3-(4-methoxyphenyl)pentanal (5)

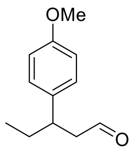

^{1}H NMR (400 MHz, $CDCl_3$): δ 9.66 (t, J=2.0 Hz, 1H), 7.10 (dt, J_1=8.8 Hz, J_2=2.4 Hz, 2H), 6.84 (dt, J_1=8.4 Hz, J_2=2.4 Hz, 2H), 3.79 (s, 3H), 3.07-2.99 (m, 1H), 2.68 (dd, J_1=7.2 Hz, J_2=2.0 Hz, 2H), 1.74-1.55 (m, 2H), 0.80 (t, J=7.6 Hz, 3H). **^{13}C NMR** (101 MHz, $CDCl_3$): δ 202.50, 158.33, 135.76, 128.56, 114.10, 55.37, 50.53, 41.15, 29.80, 12.01. **MS** (ESI): m/z for $C_{12}H_{16}O_2$ $[M]^+$ calcd 192.12, found 192.14.

2. *Kinetic poisoning experiments*:

Mercury or poly(4-vinylpyridine) (20 equiv) was introduced at 0 or 10 h, with two parallel standard reactions conducted for comparison. After 24 h, 1,3,5-trimethoxybenzene was added as the internal standard, and yields were determined by GC-MS.

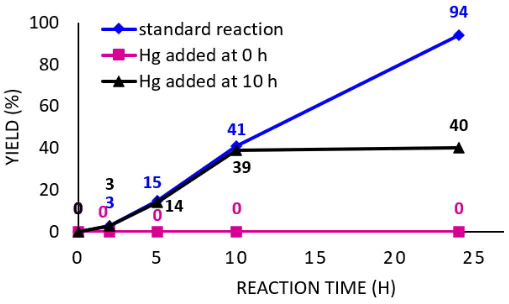

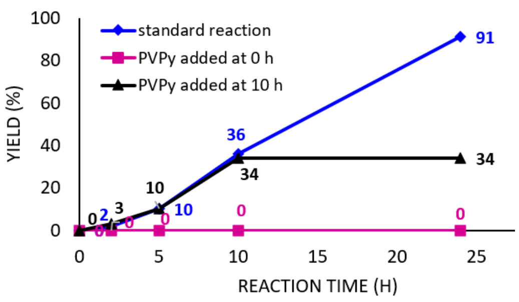

6. NMR Spectra

3-(4-(benzyloxy)phenyl)pentanal (3a)

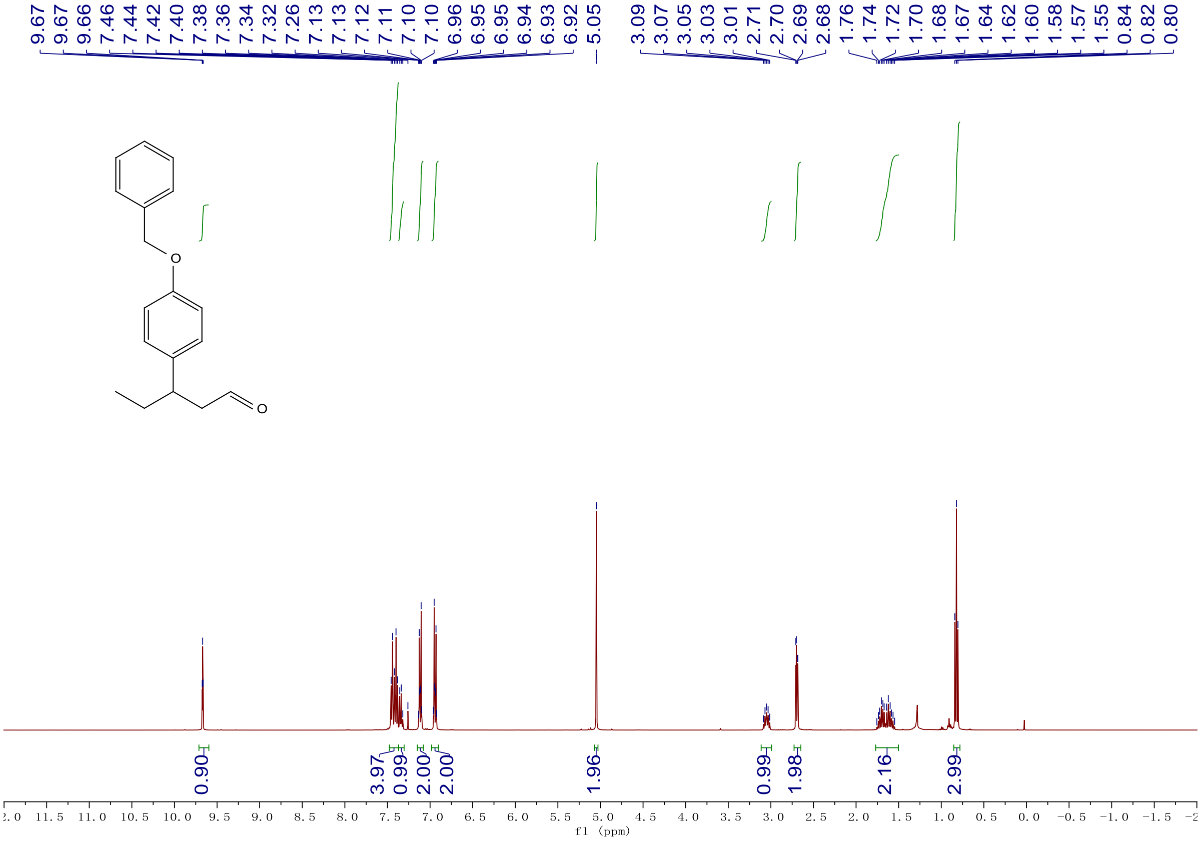

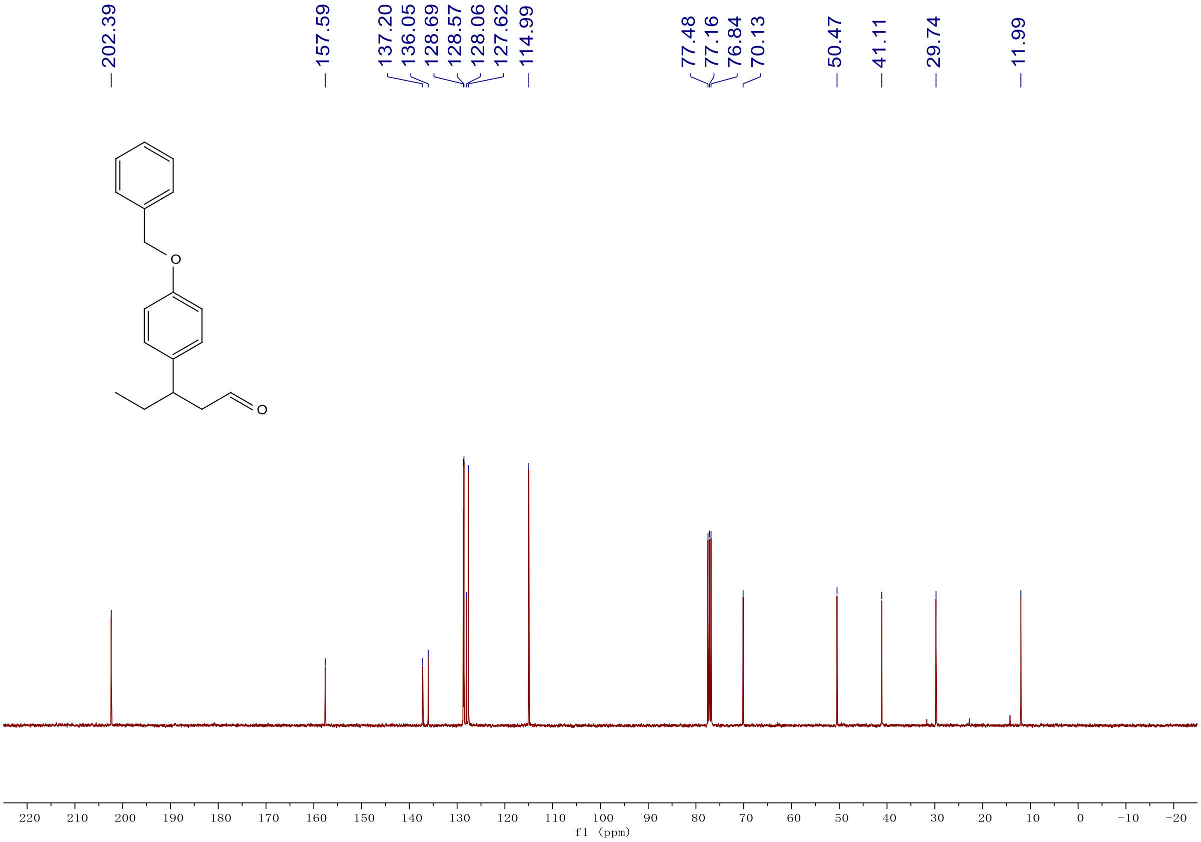

3-([1,1'-biphenyl]-4-yl)pentanal (3b)

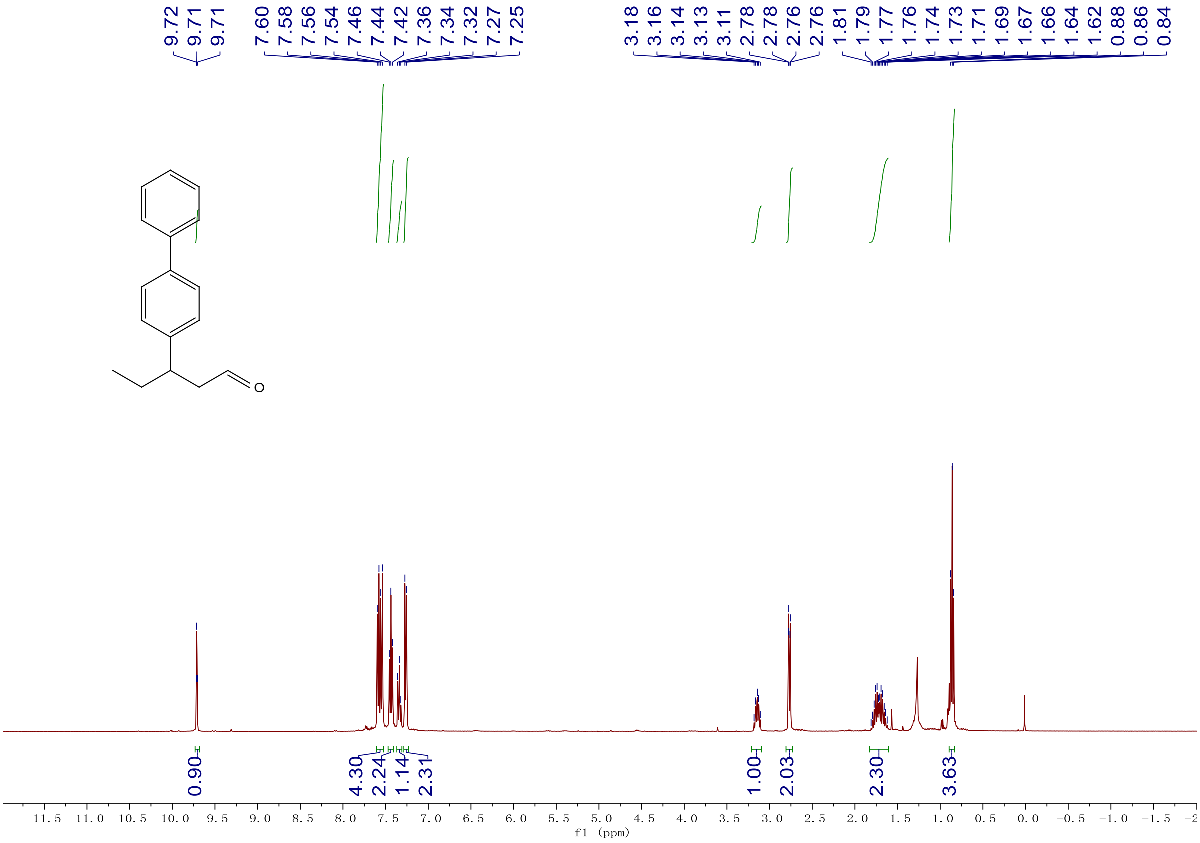

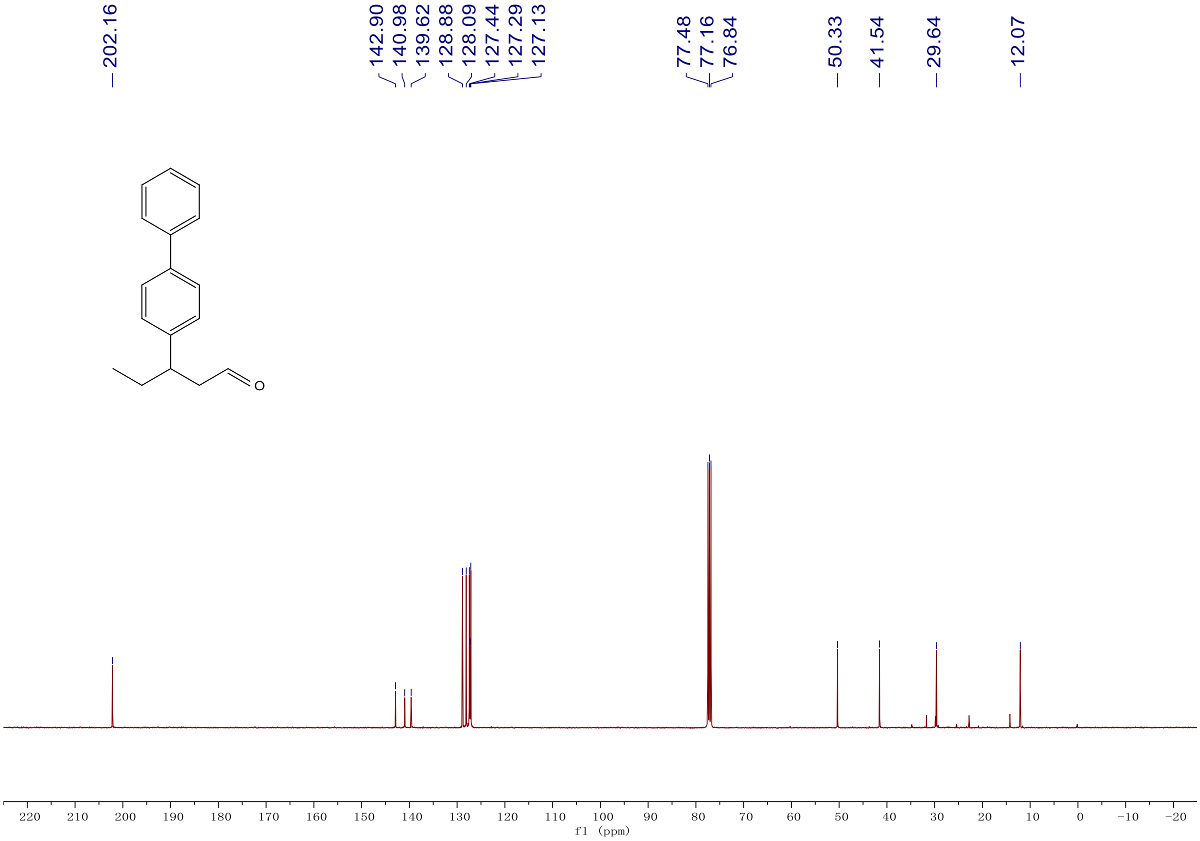

3-(o-tolyl)pentanal (3c)

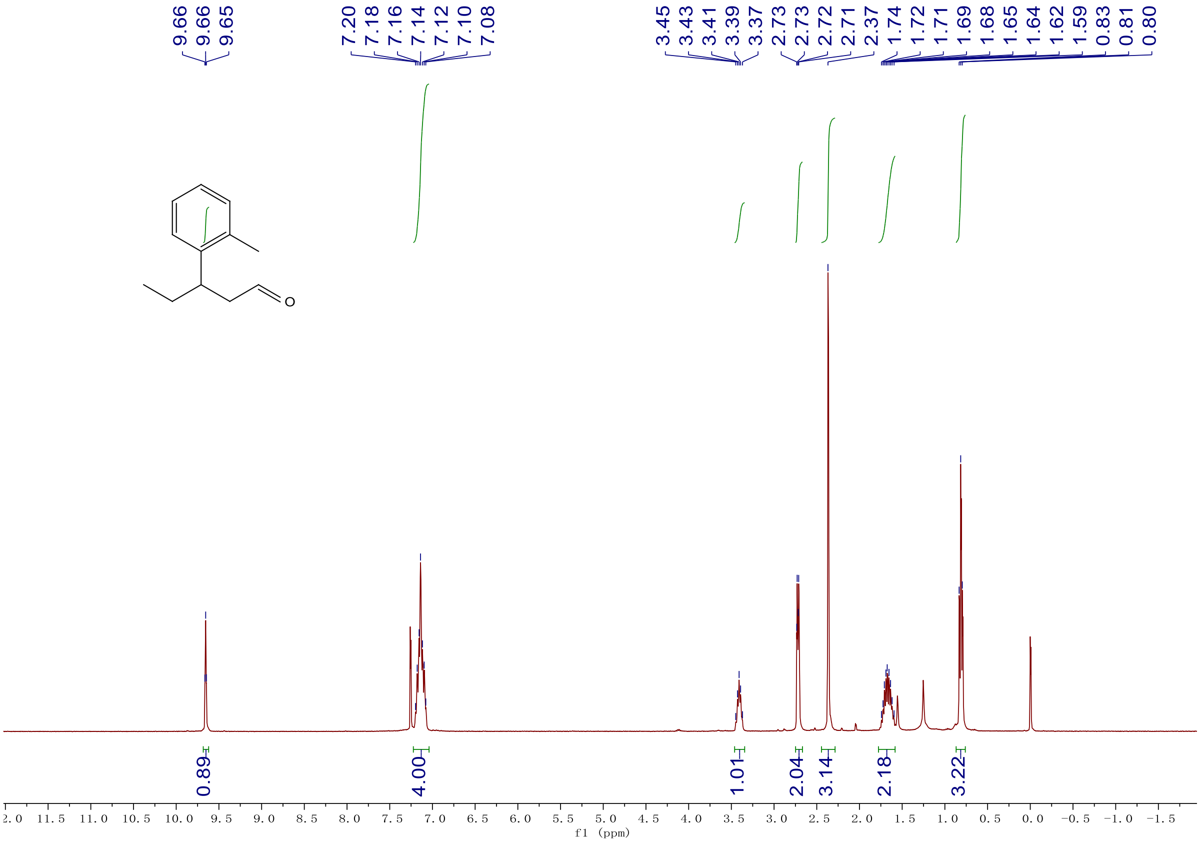

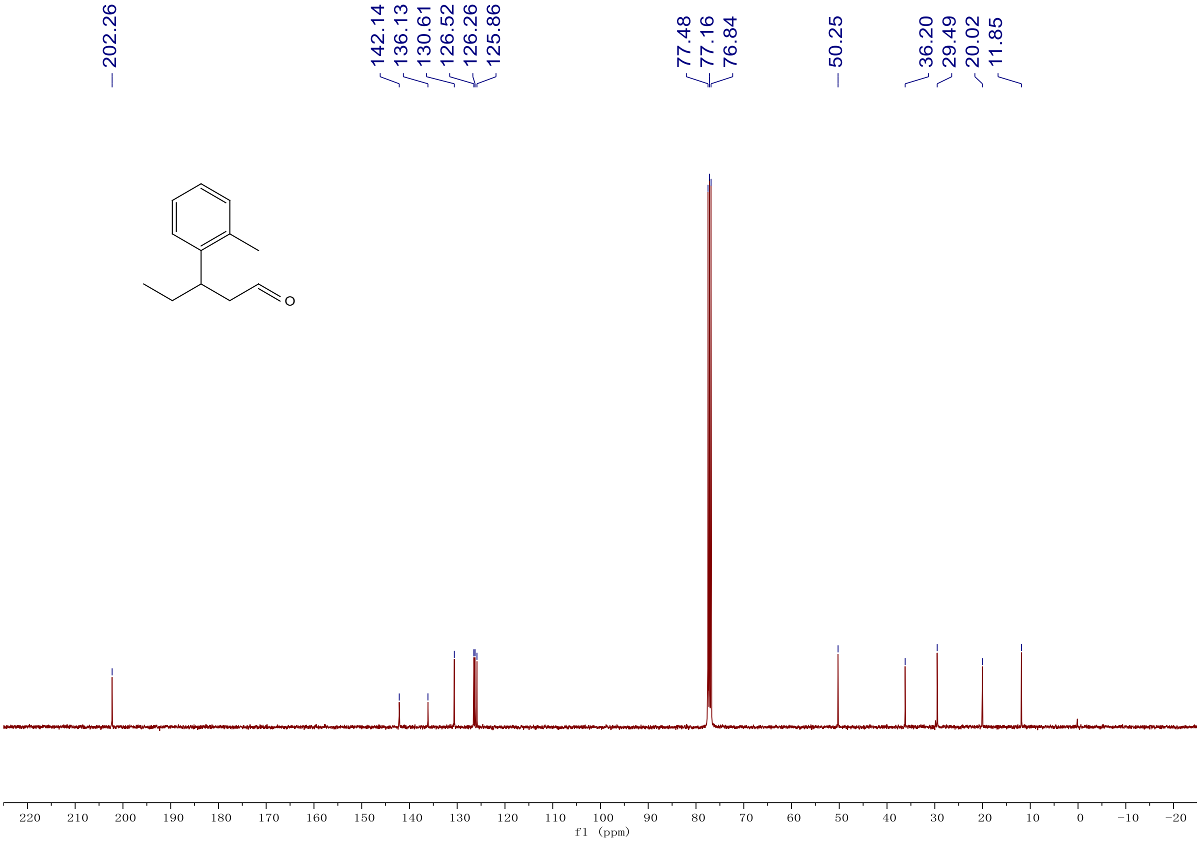

3-(*m*-tolyl)pentanal (3d)

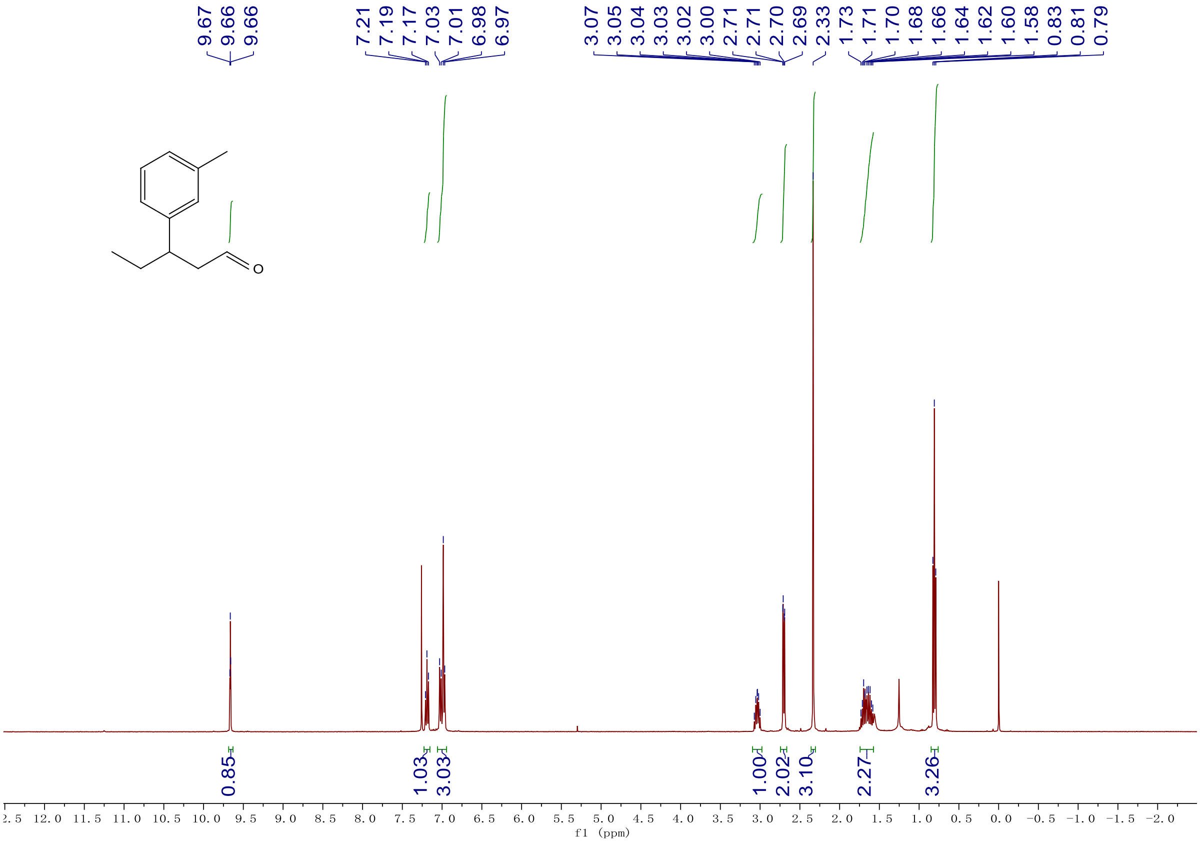

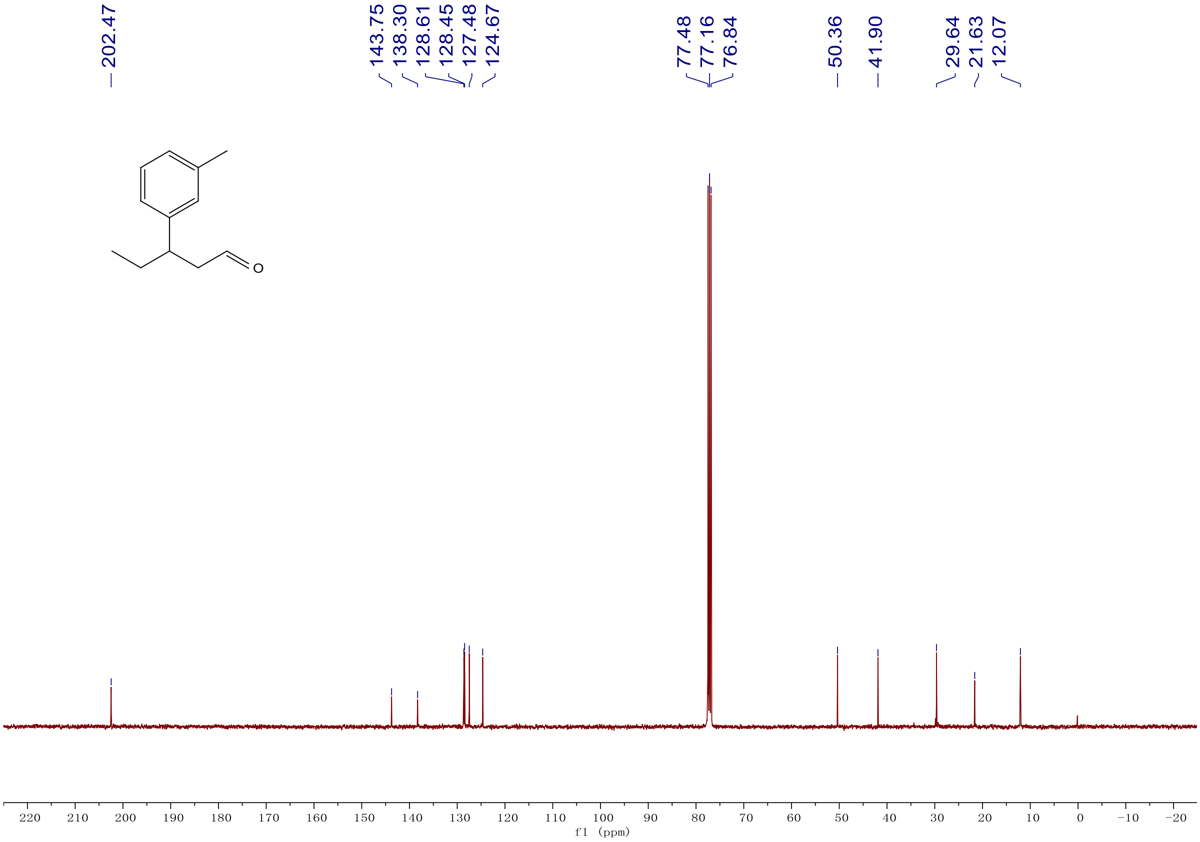

3-(4-heptylphenyl)pentanal (3e)

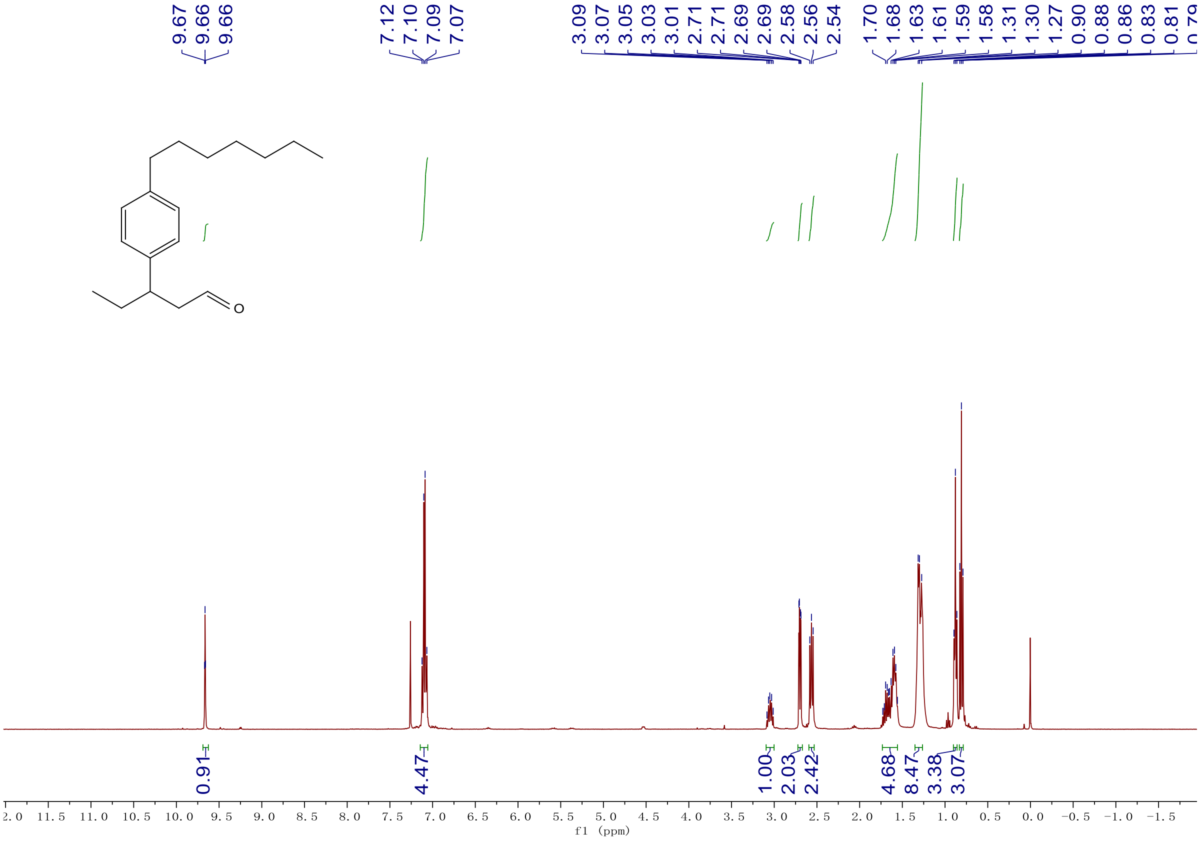

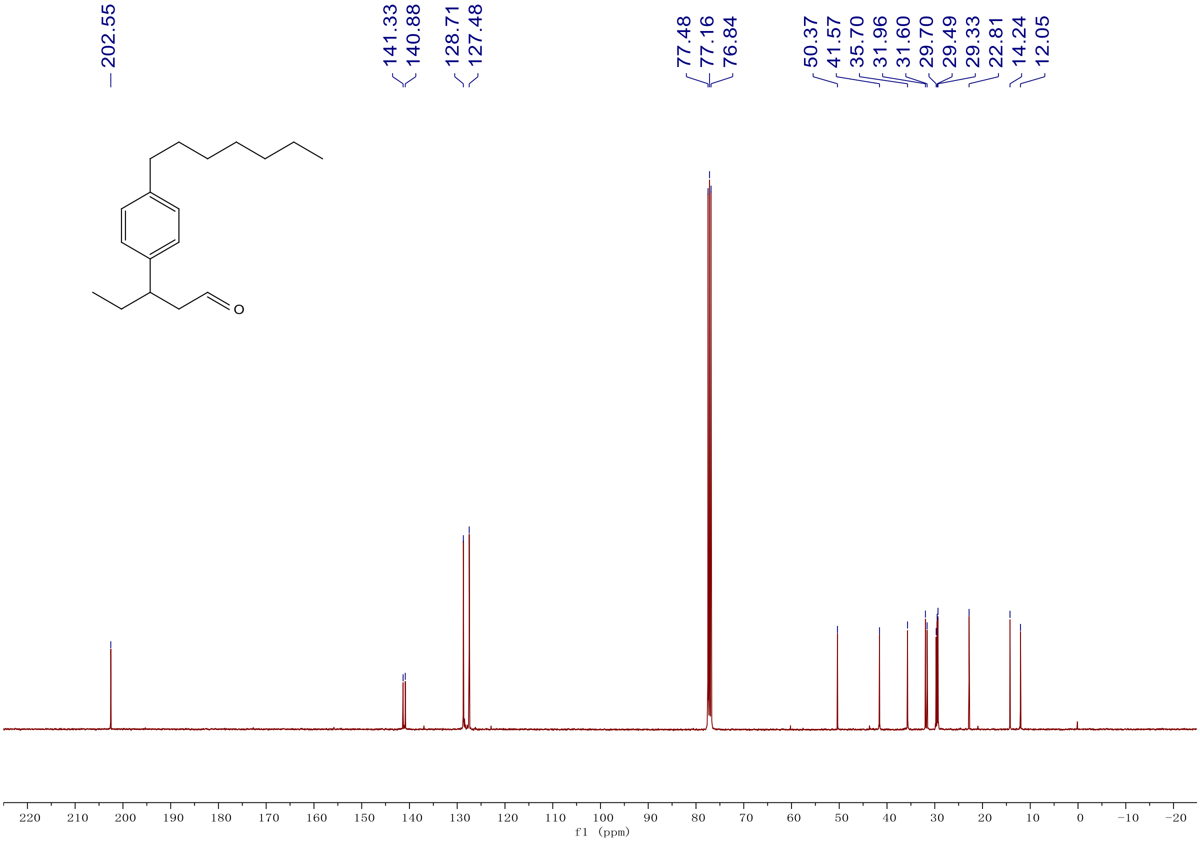

3-(4-(*tert*-butyl)phenyl)pentanal (3f)

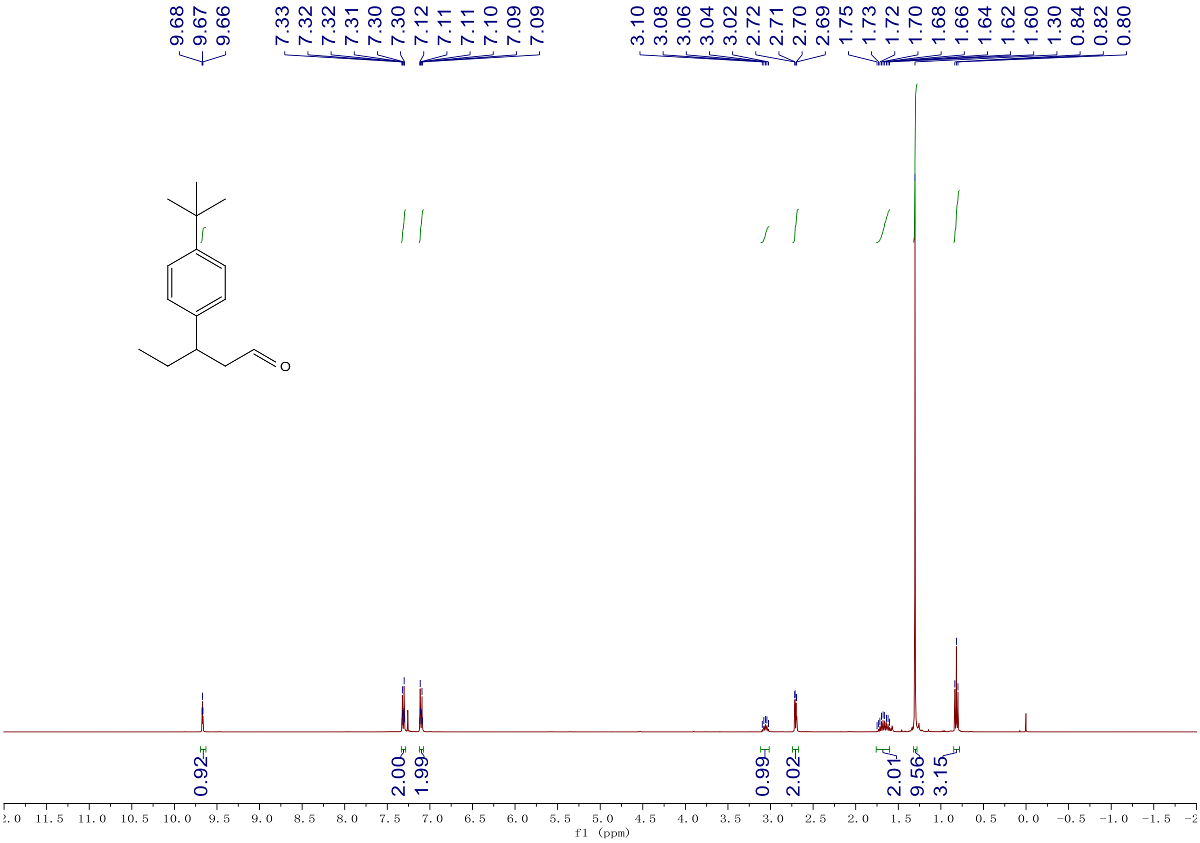

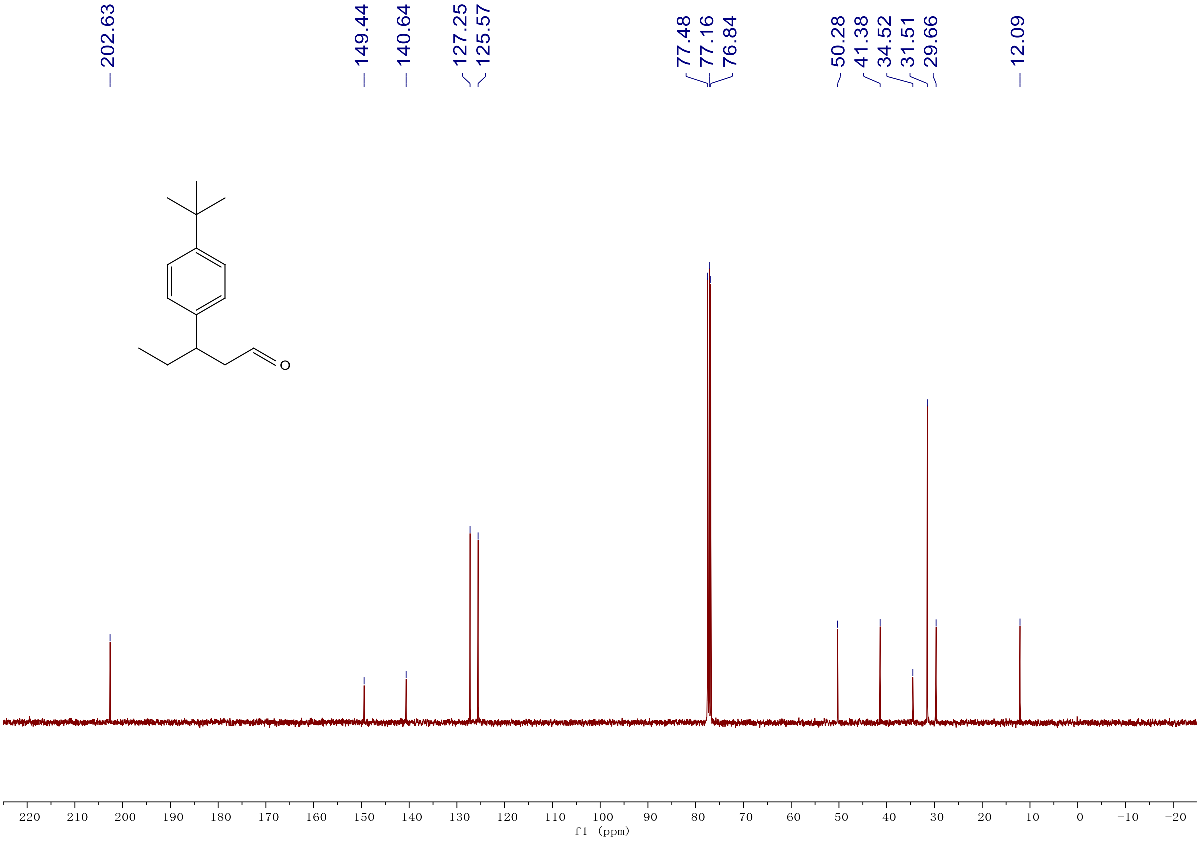

3-(3-methoxyphenyl)pentanal (3g)

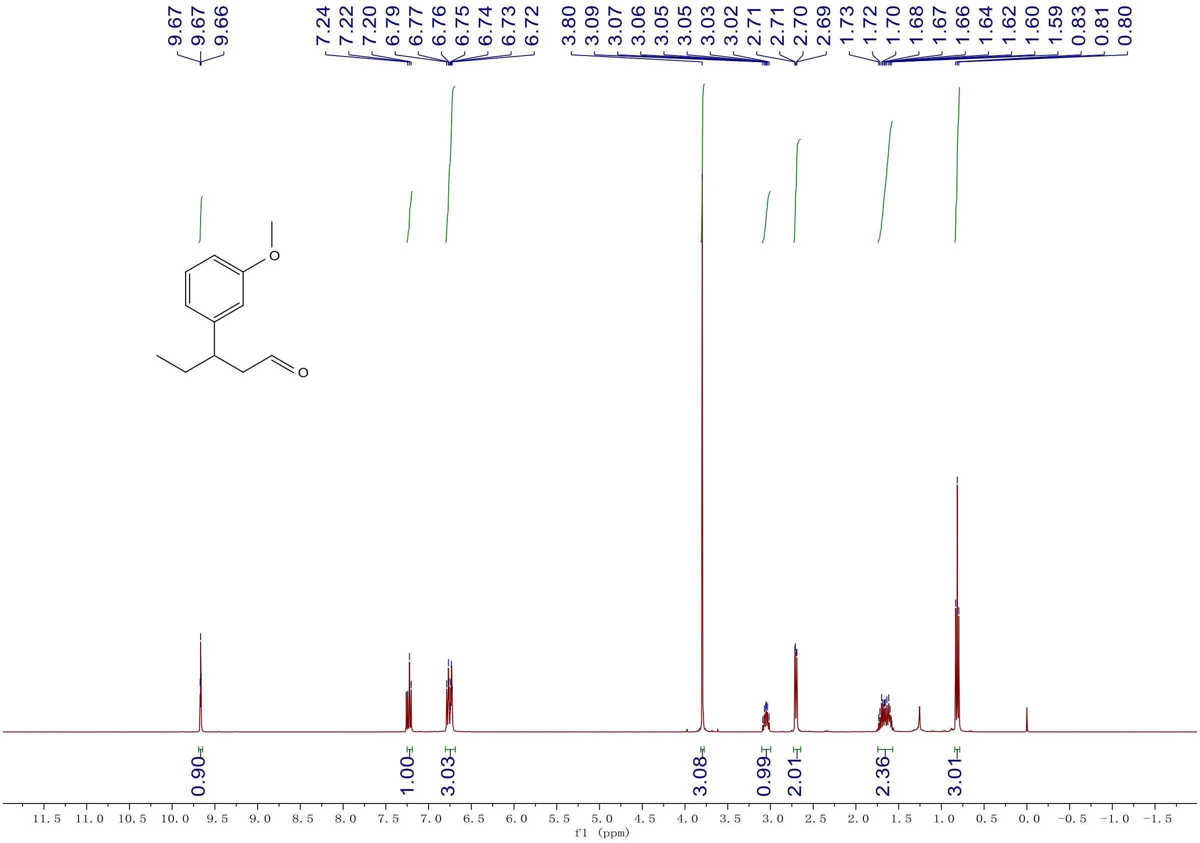

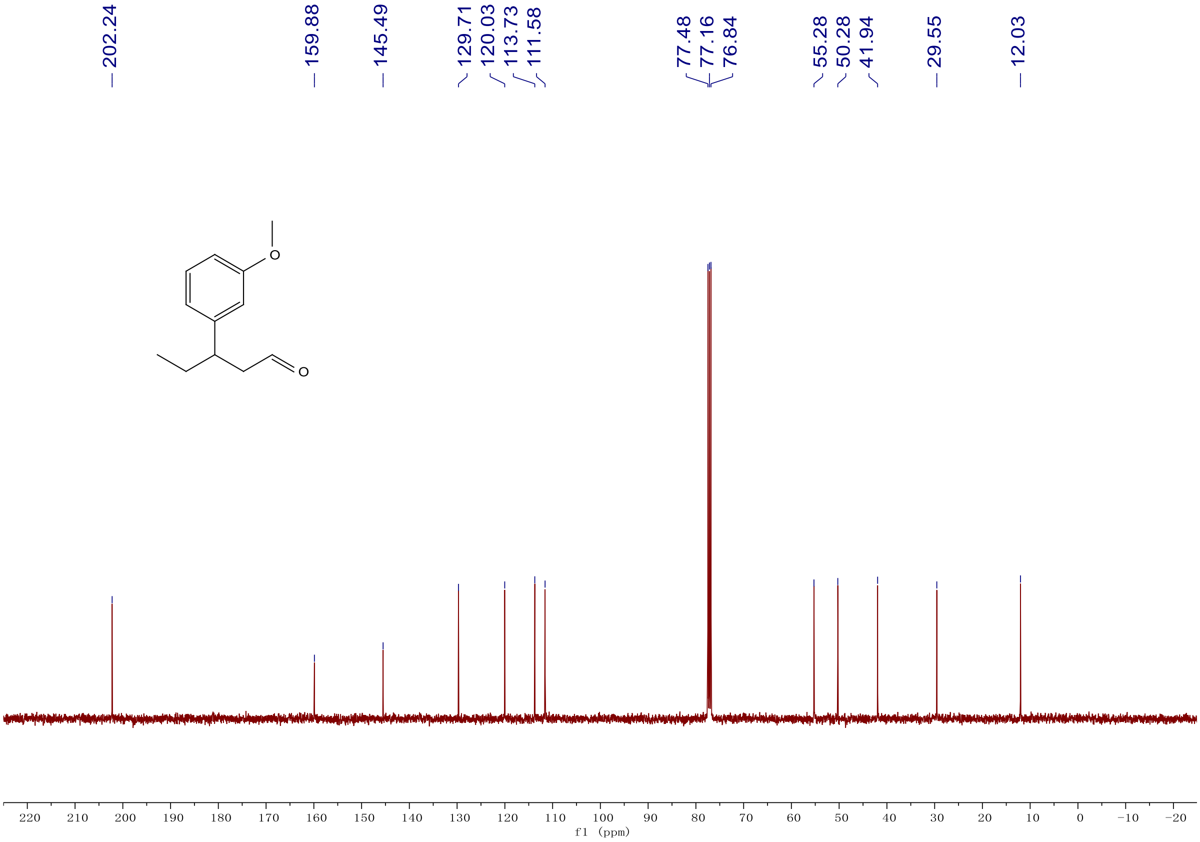

3-(4-fluorophenyl)pentanal (3h)

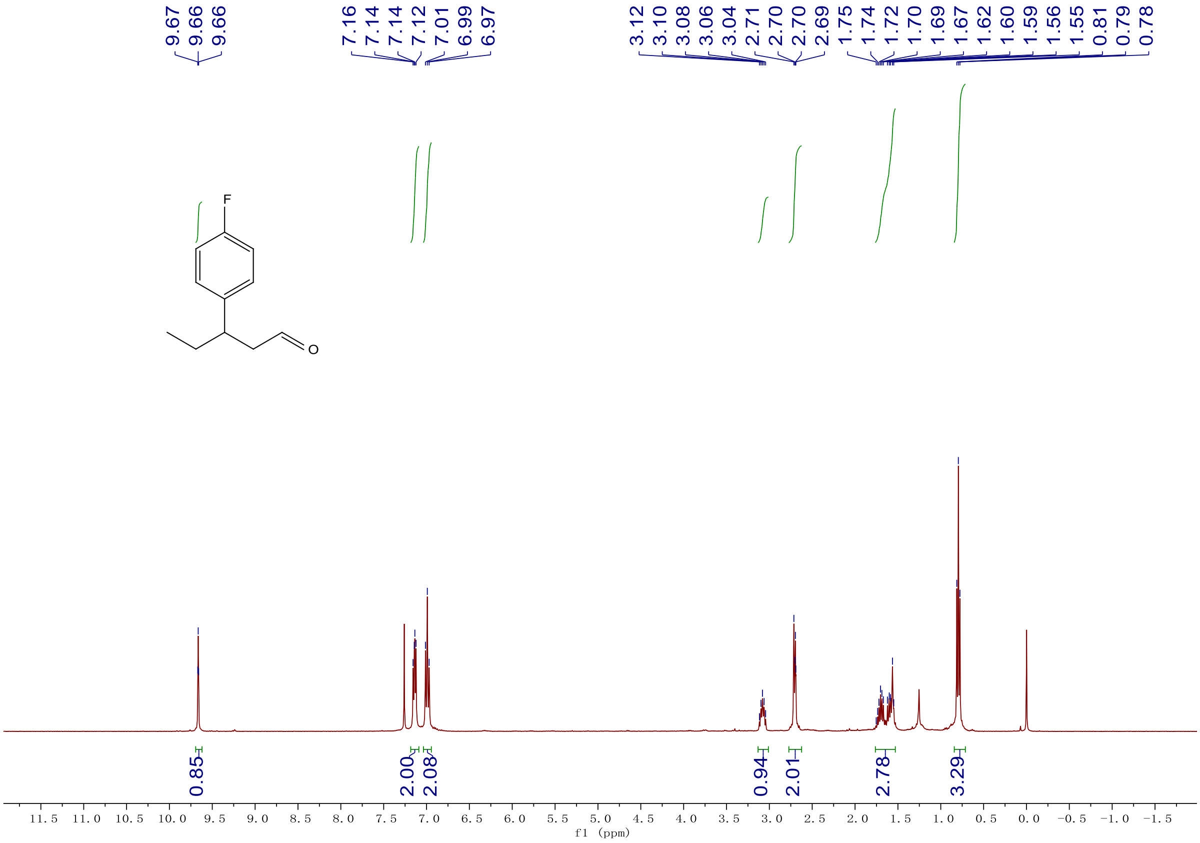

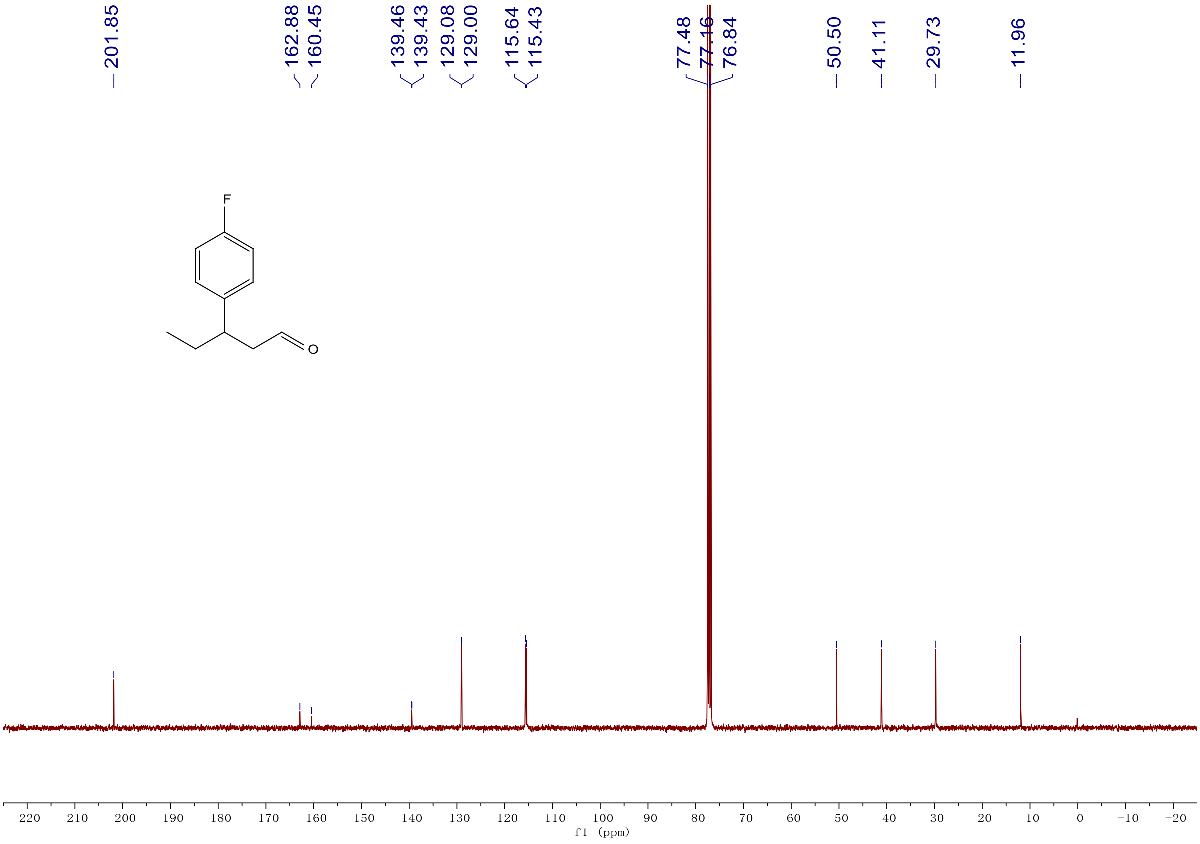

3-(4-chlorophenyl)pentanal (3i)

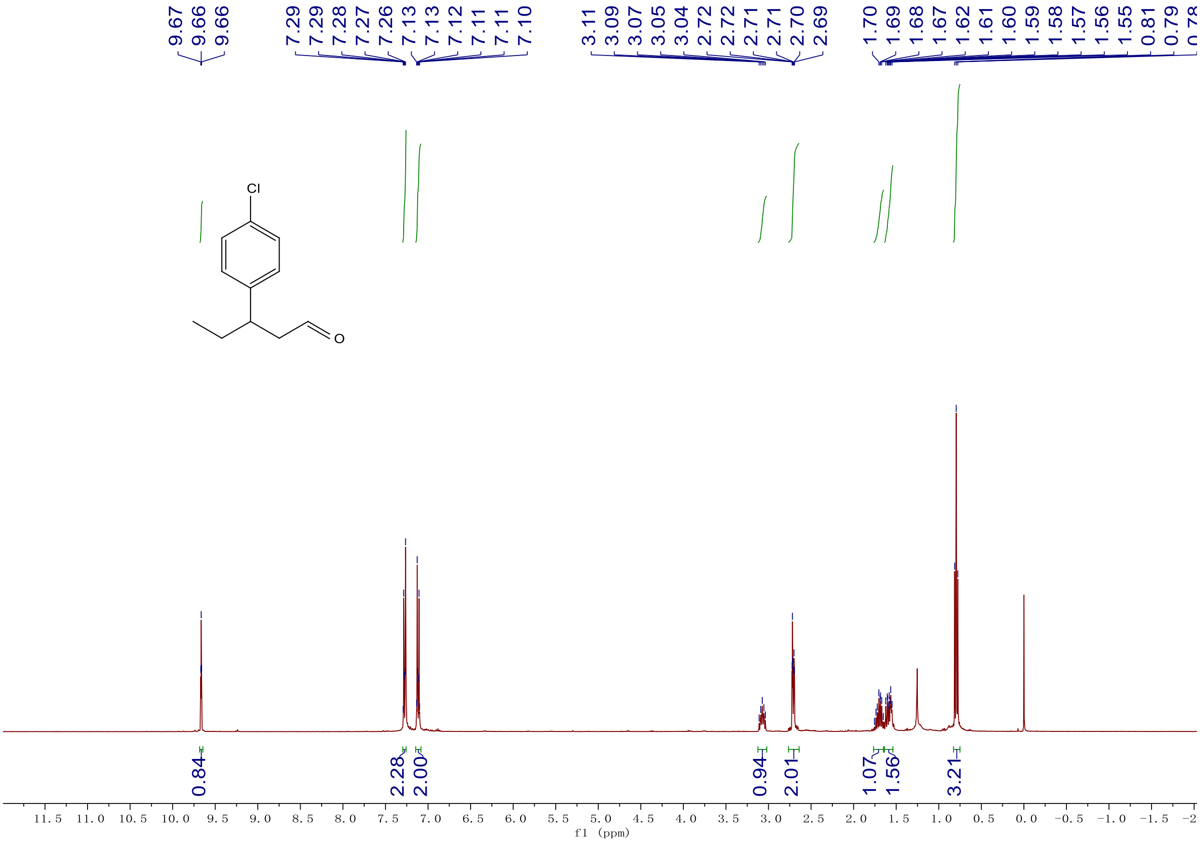

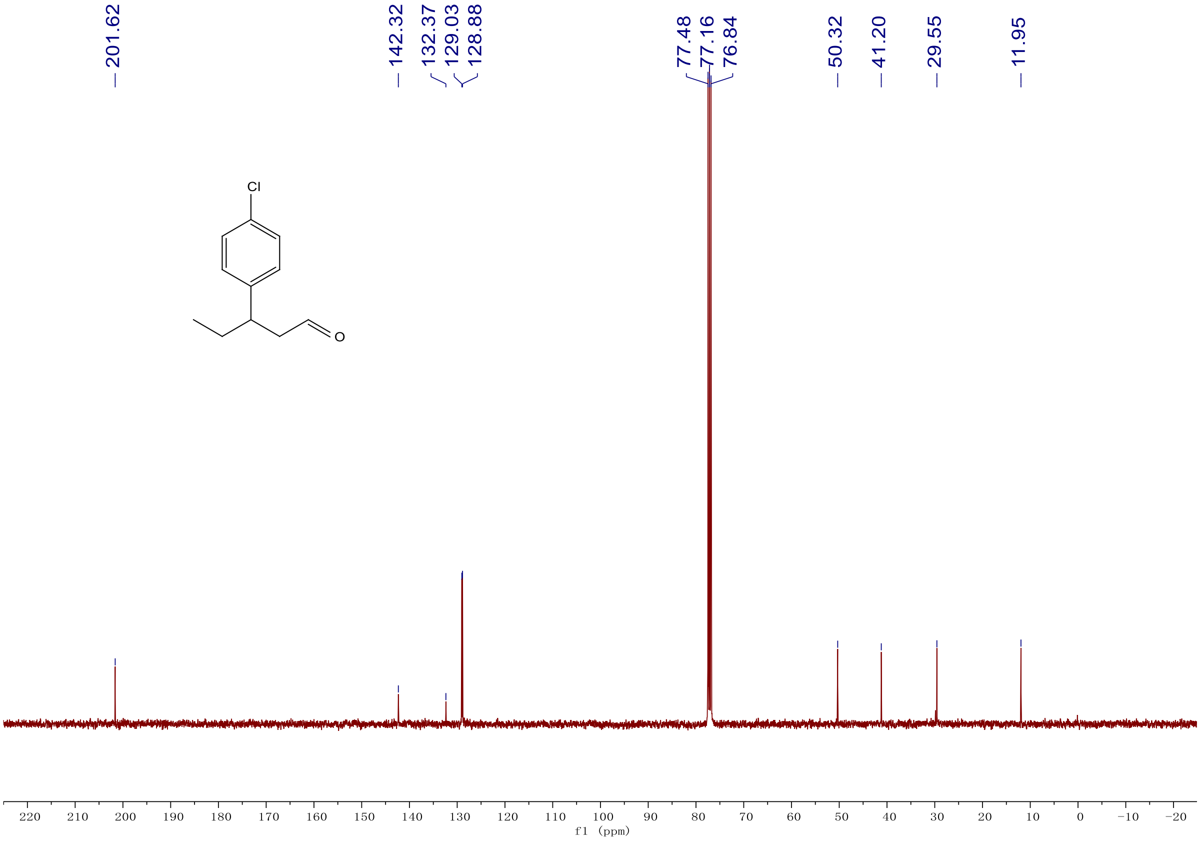

3-(4-bromophenyl)pentanal (3j)

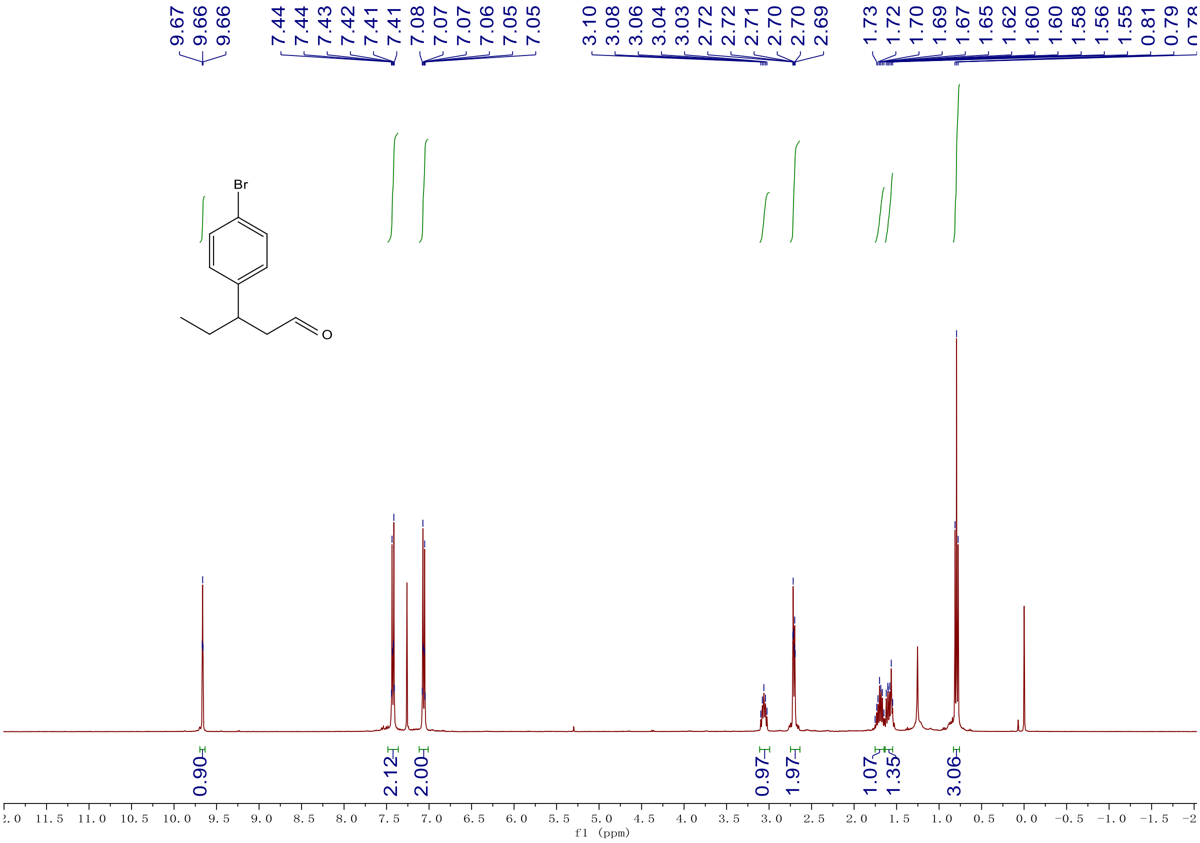

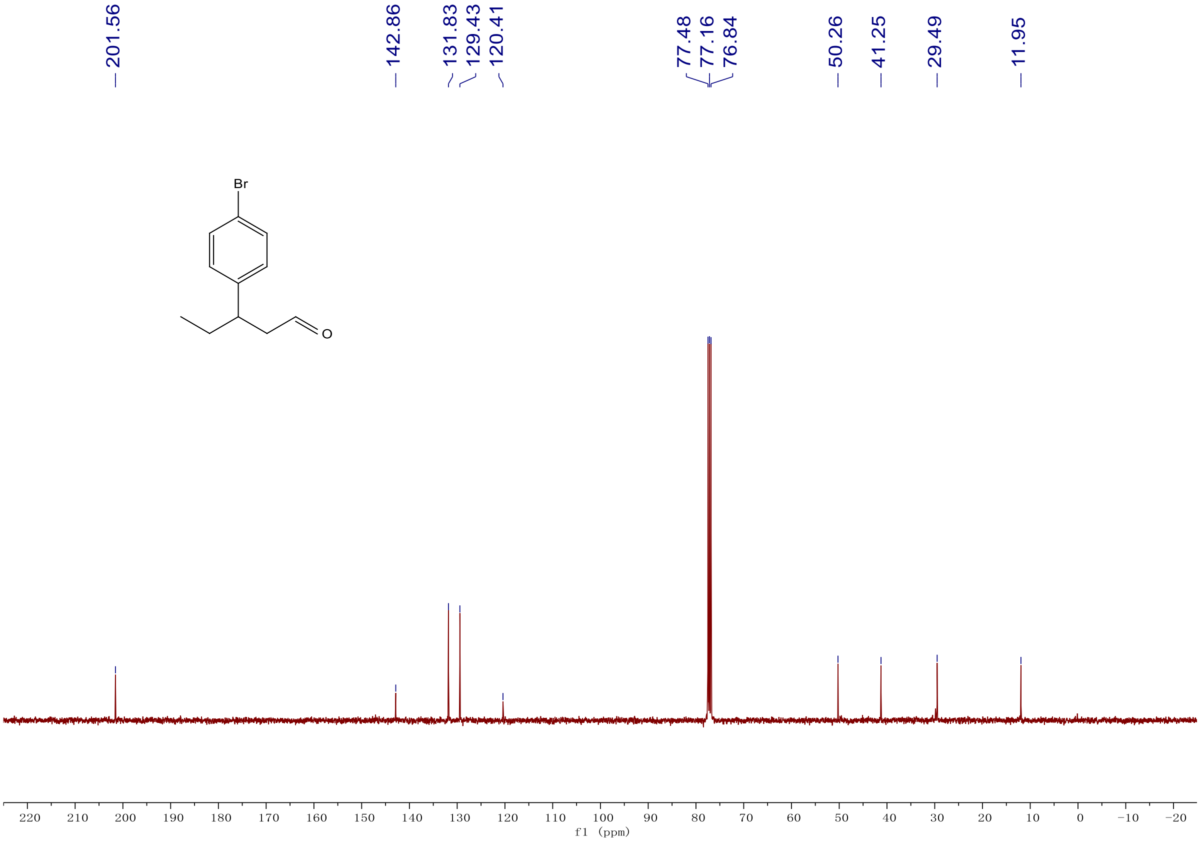

3-(2-fluorophenyl)pentanal (3k)

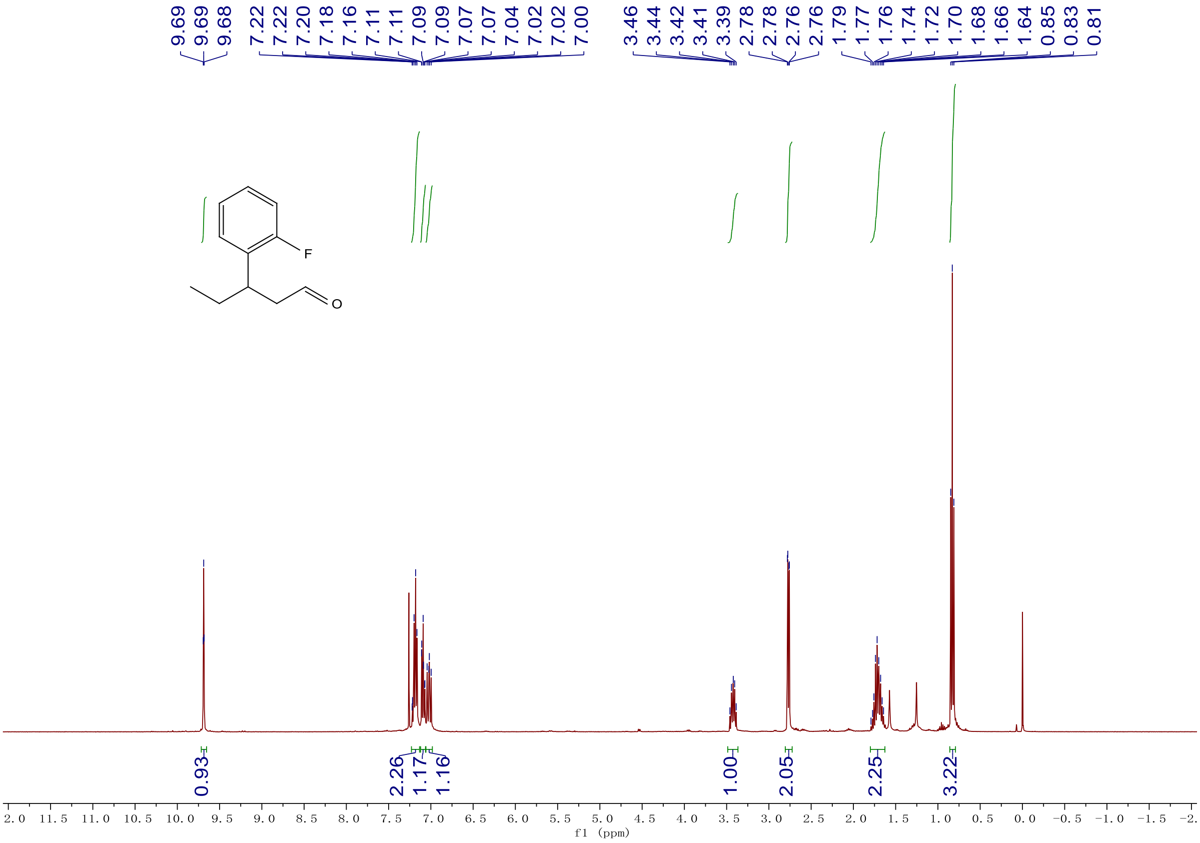

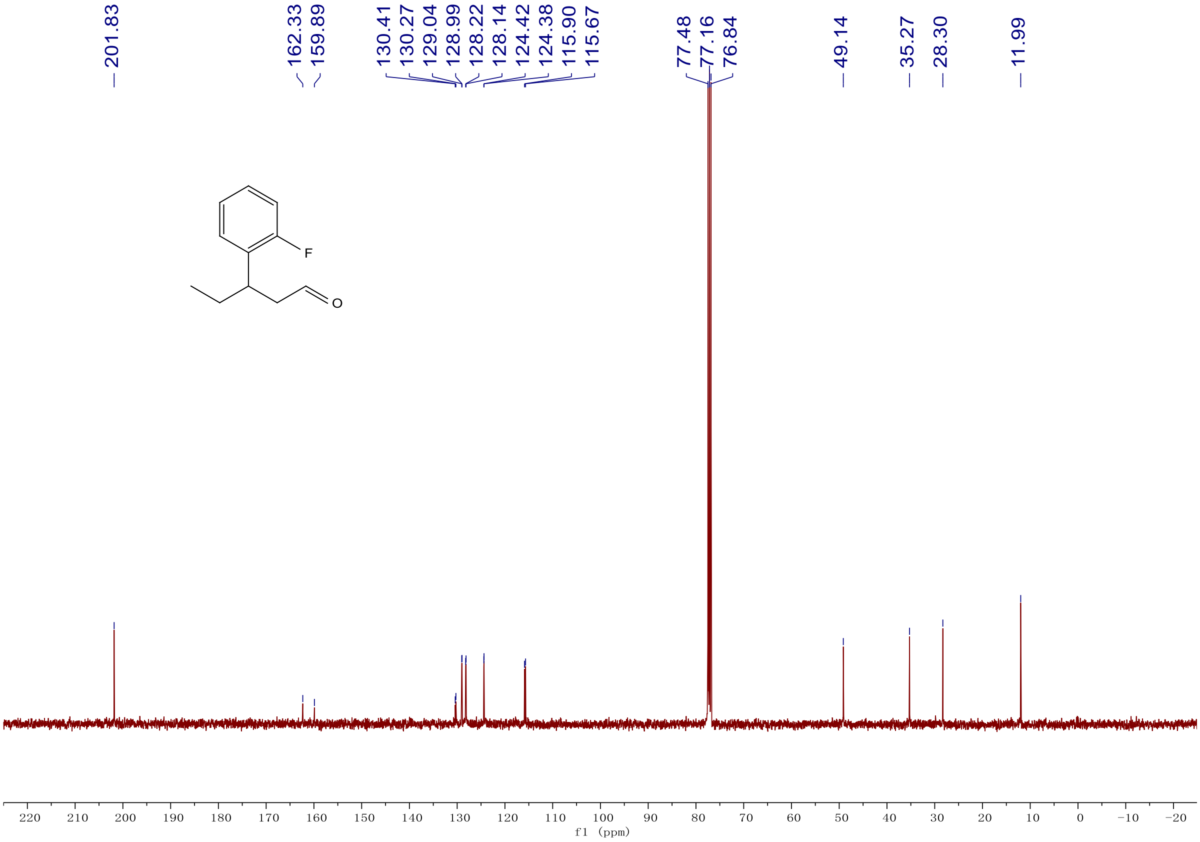

3-(3-chlorophenyl)pentanal (3l)

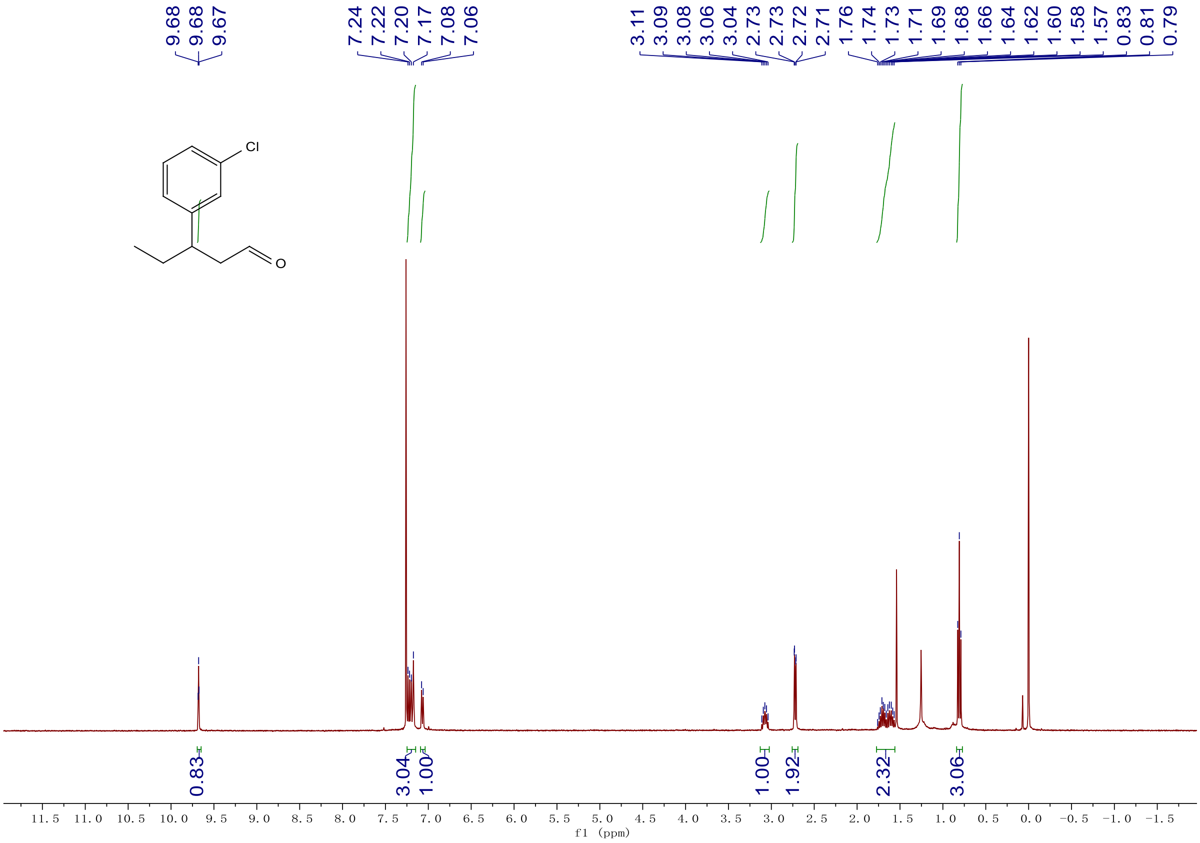

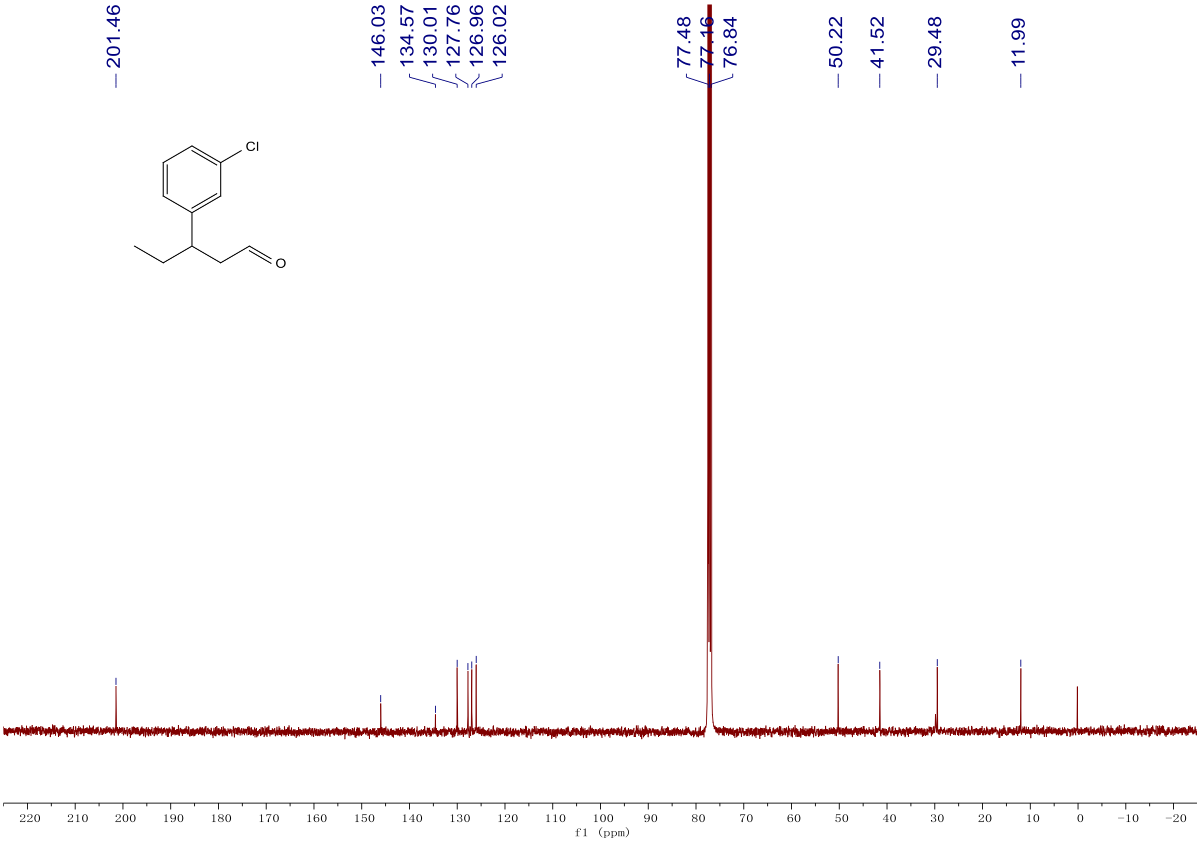

3-(3-bromophenyl)pentanal (3m)

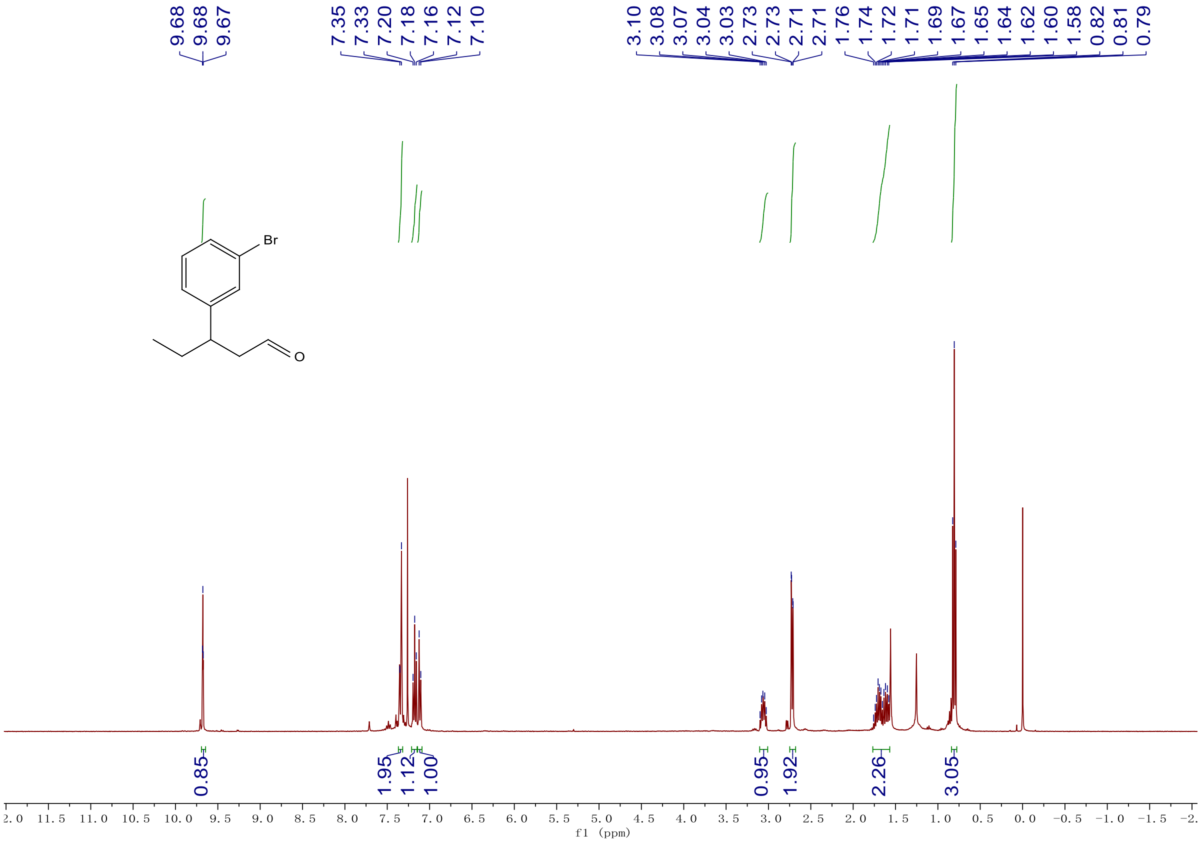

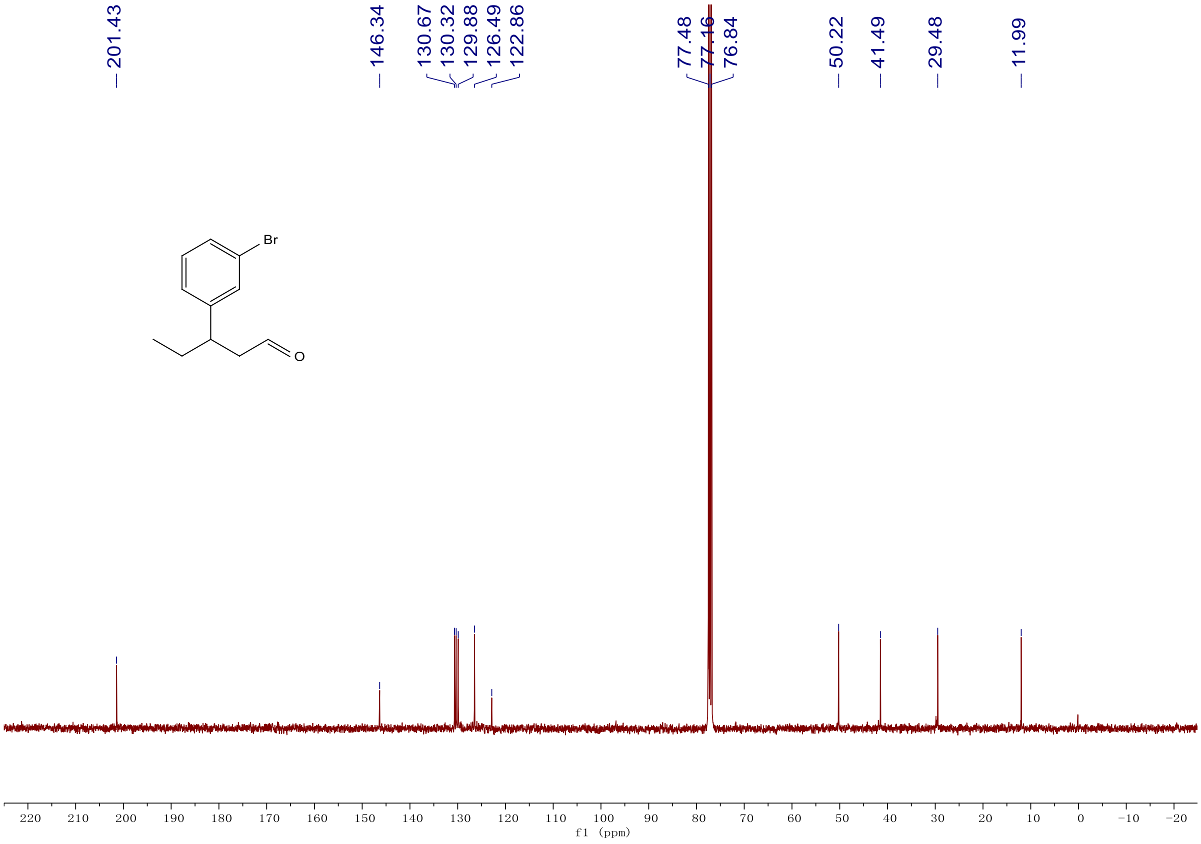

4-(1-oxopentan-3-yl)benzonitrile (3n)

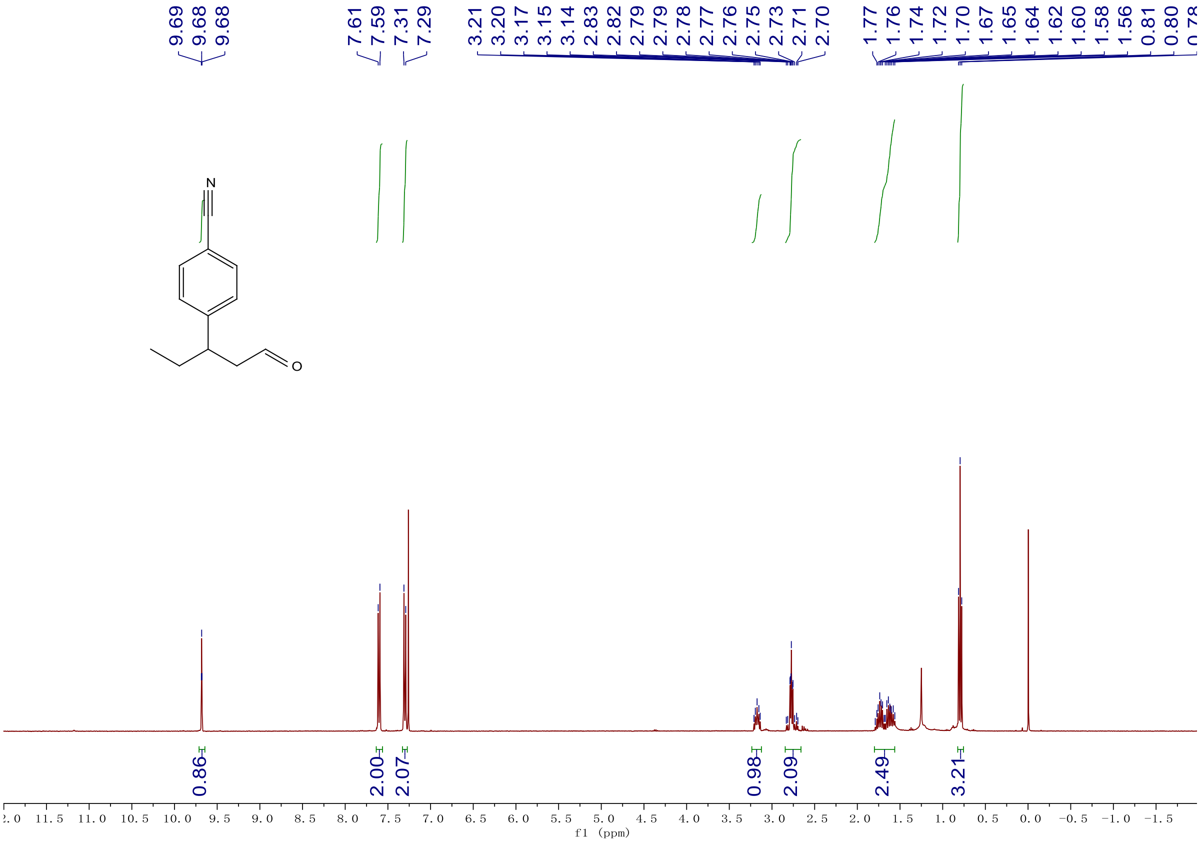

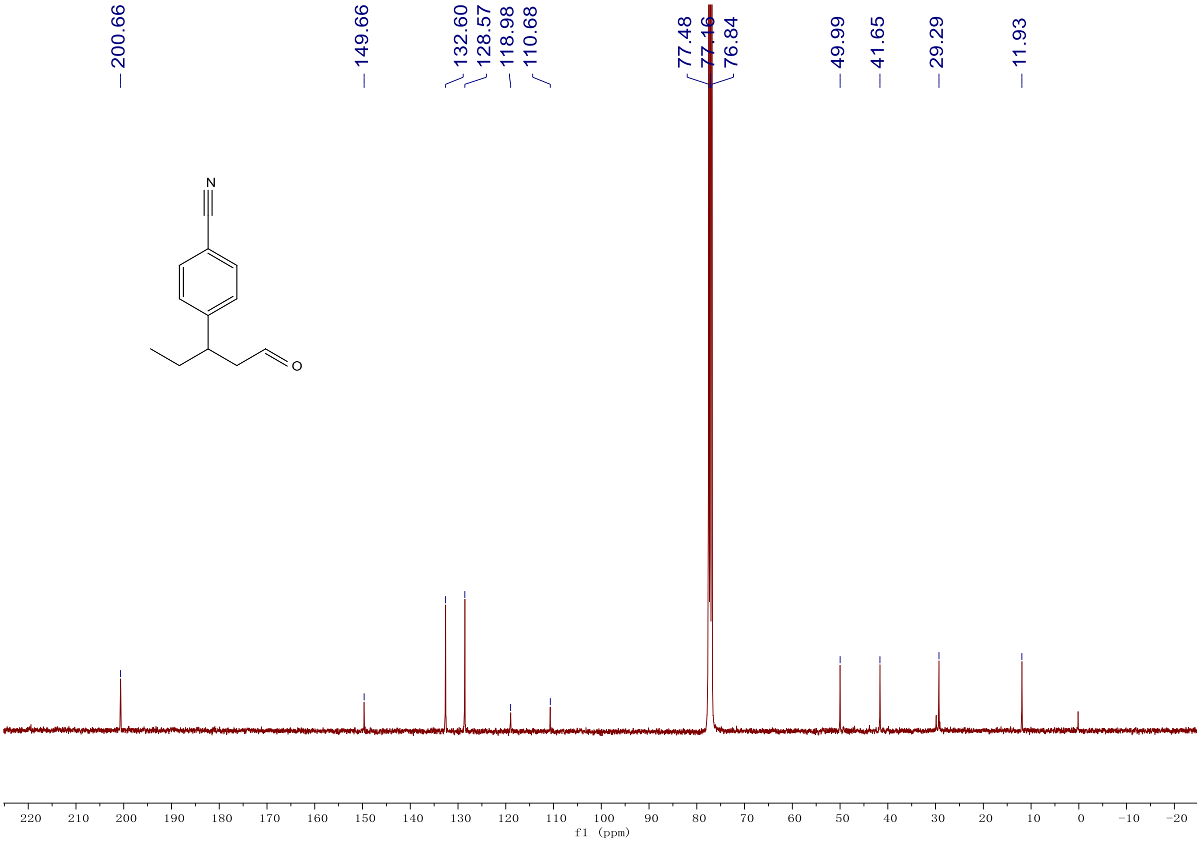

N-(4-(1-oxopentan-3-yl)phenyl)acetamide (3o)

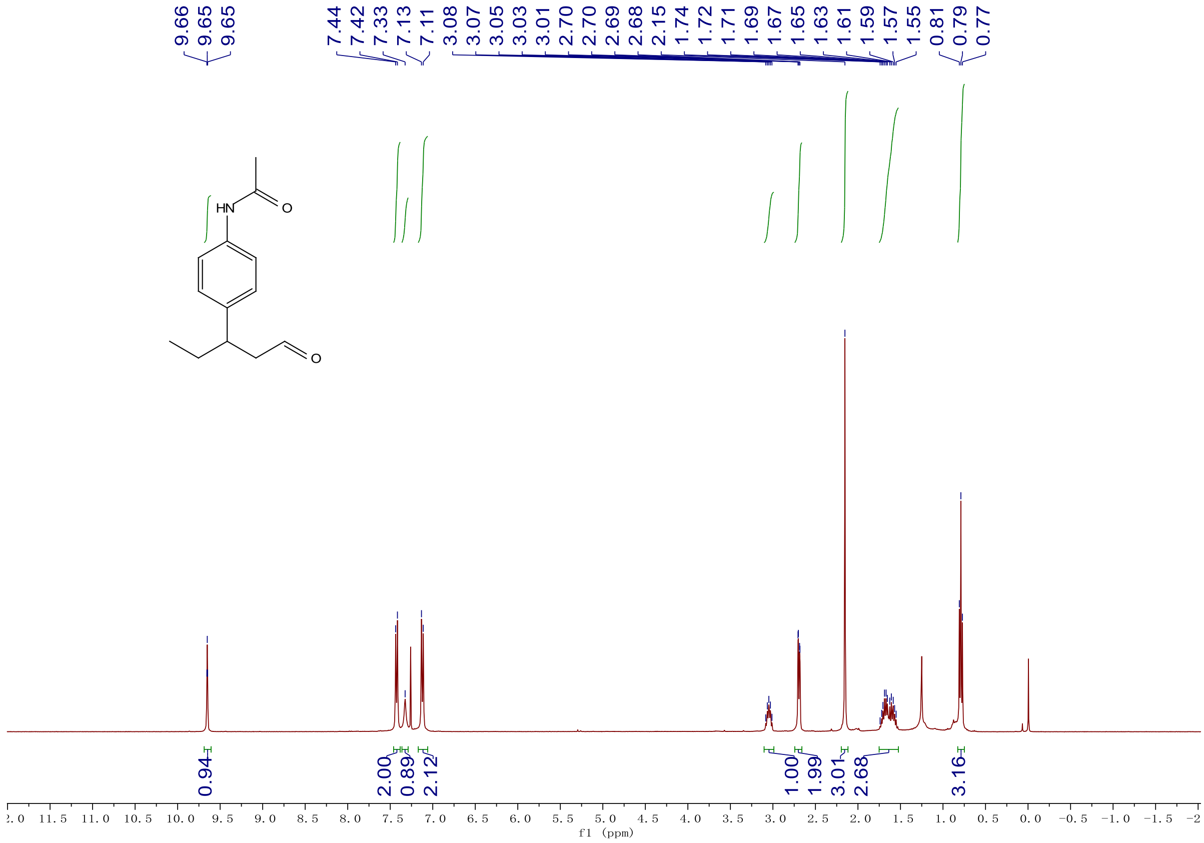

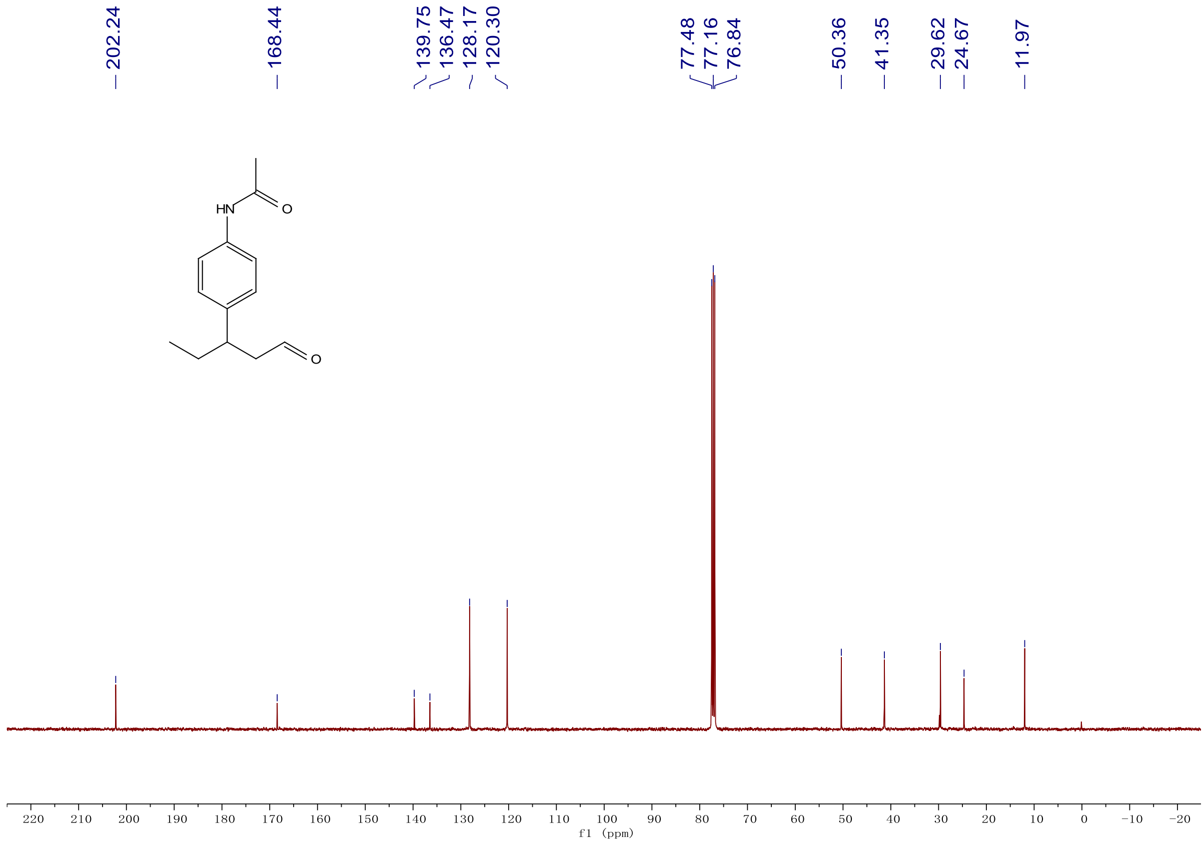

3-(naphthalen-1-yl)pentanal (3p)

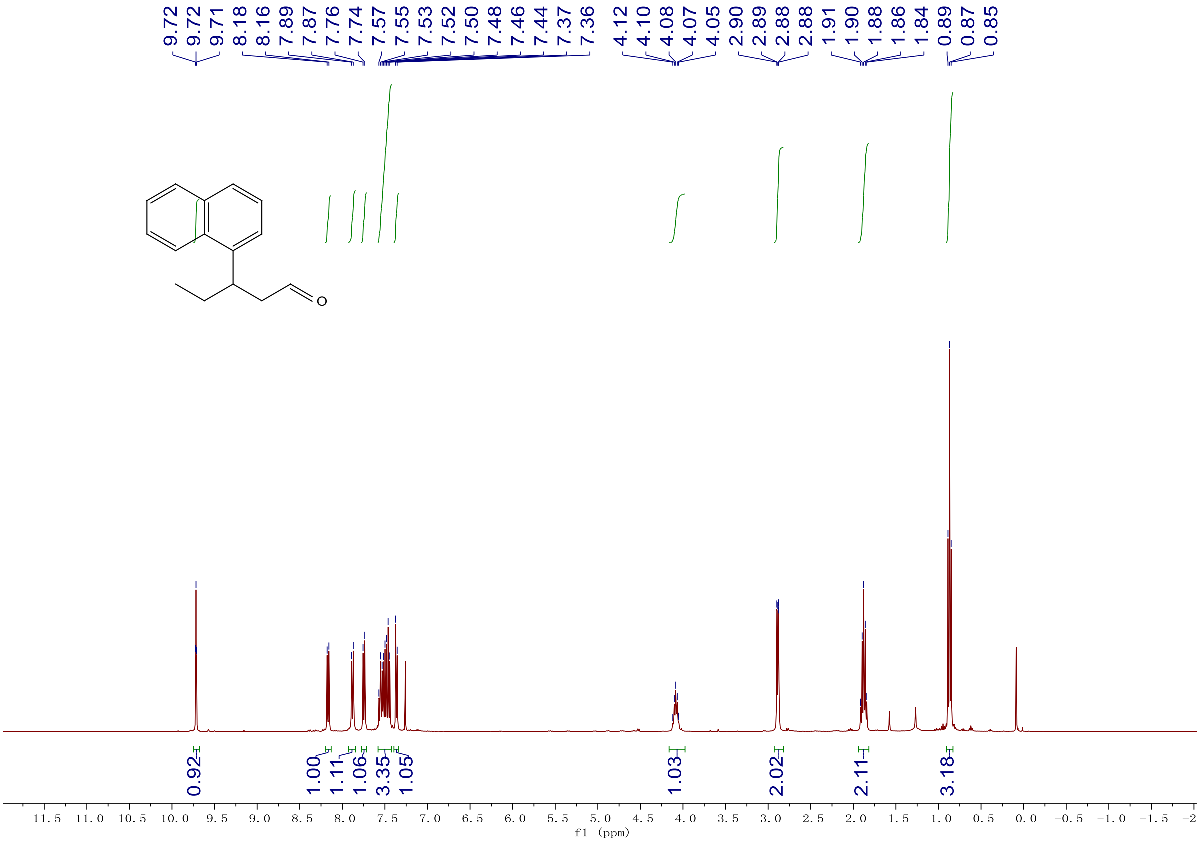

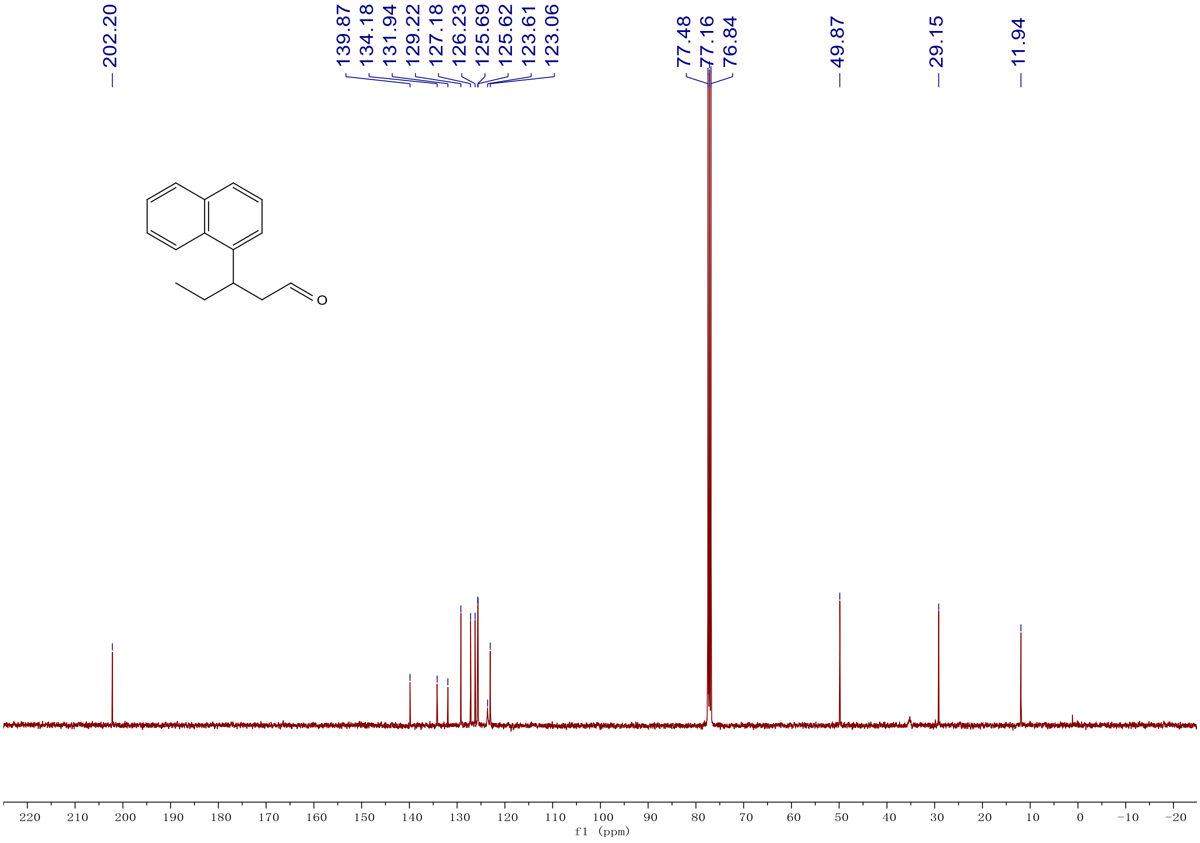

3-(naphthalen-2-yl)pentanal (3q)

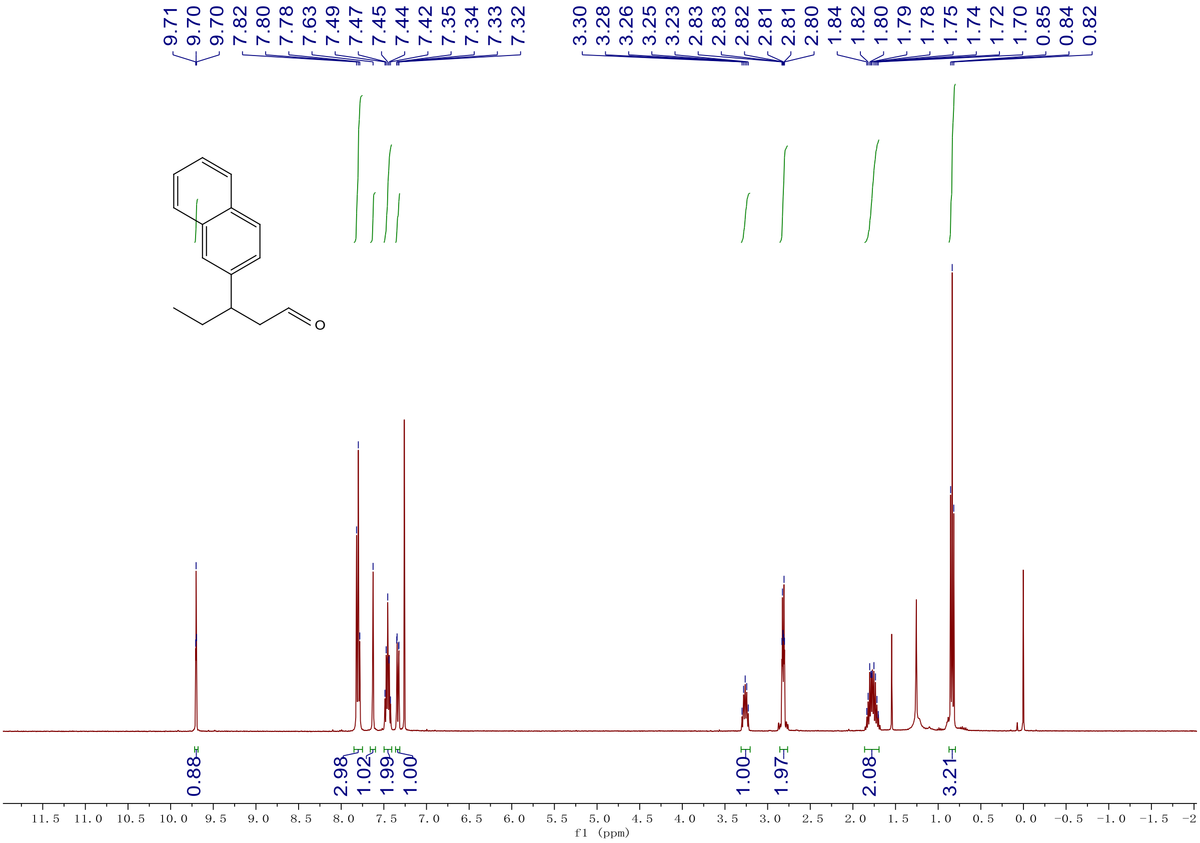

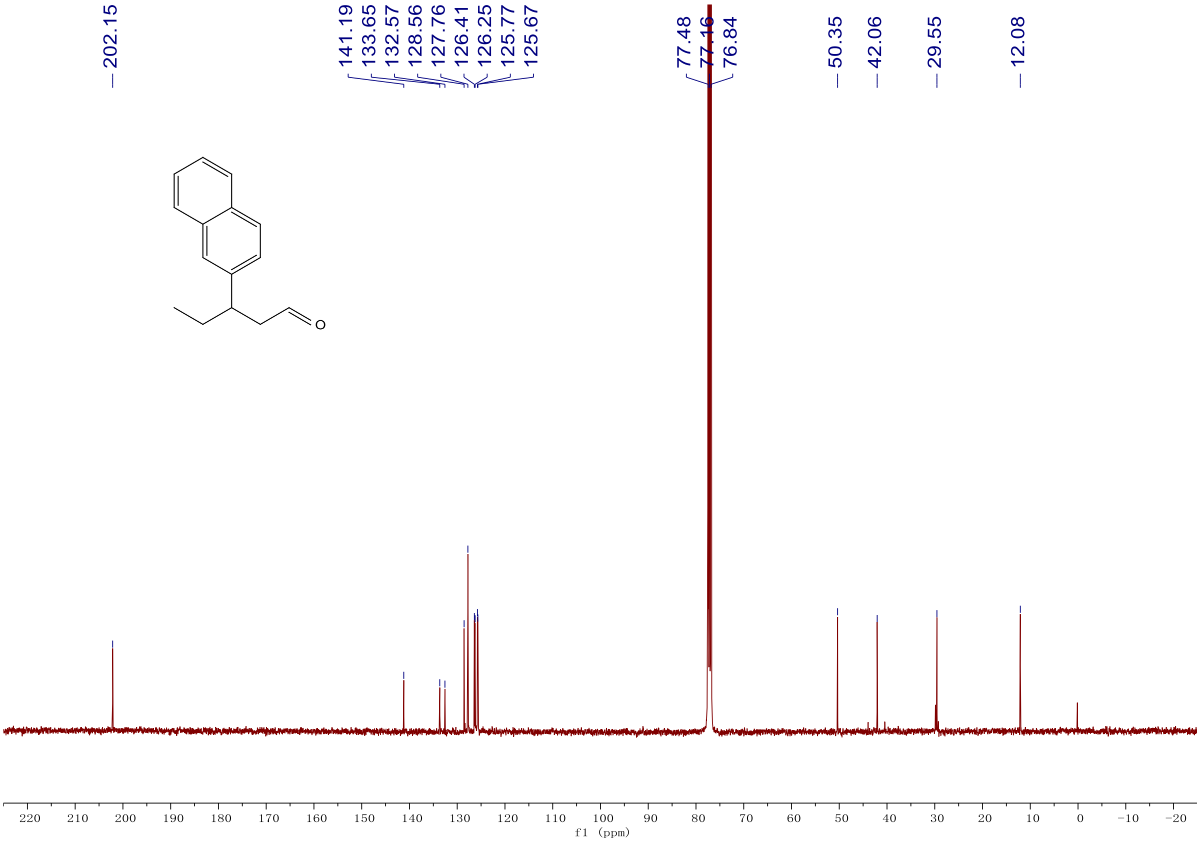

3-(thiophen-3-yl)pentanal (3r)

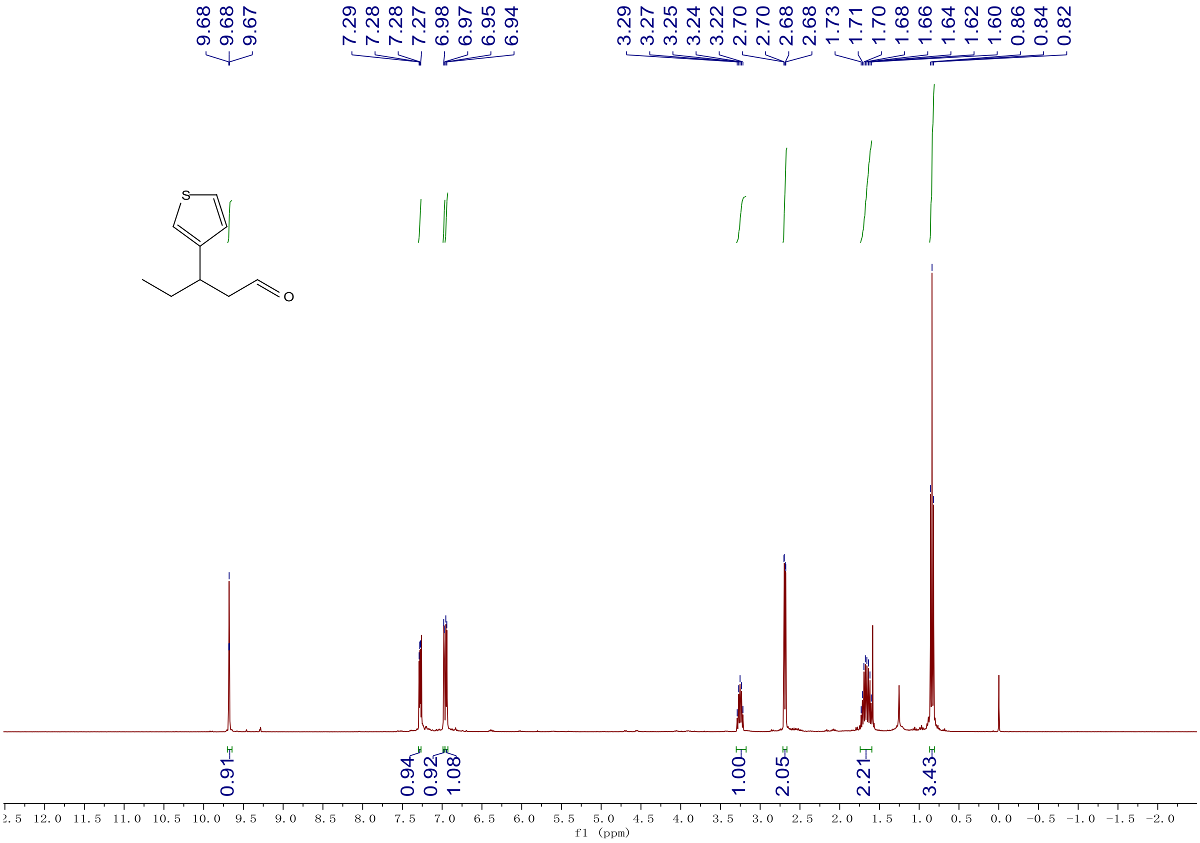

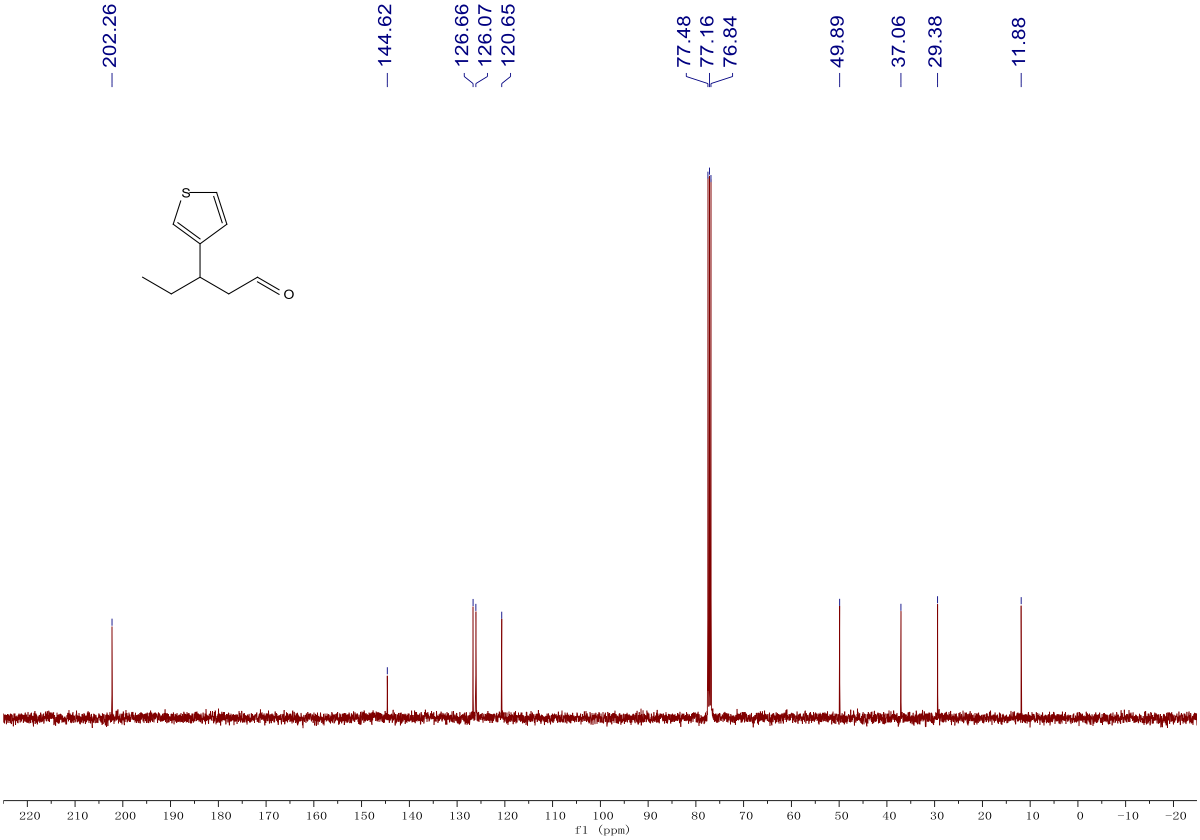

3-(4-methoxyphenyl)octanal (3s)

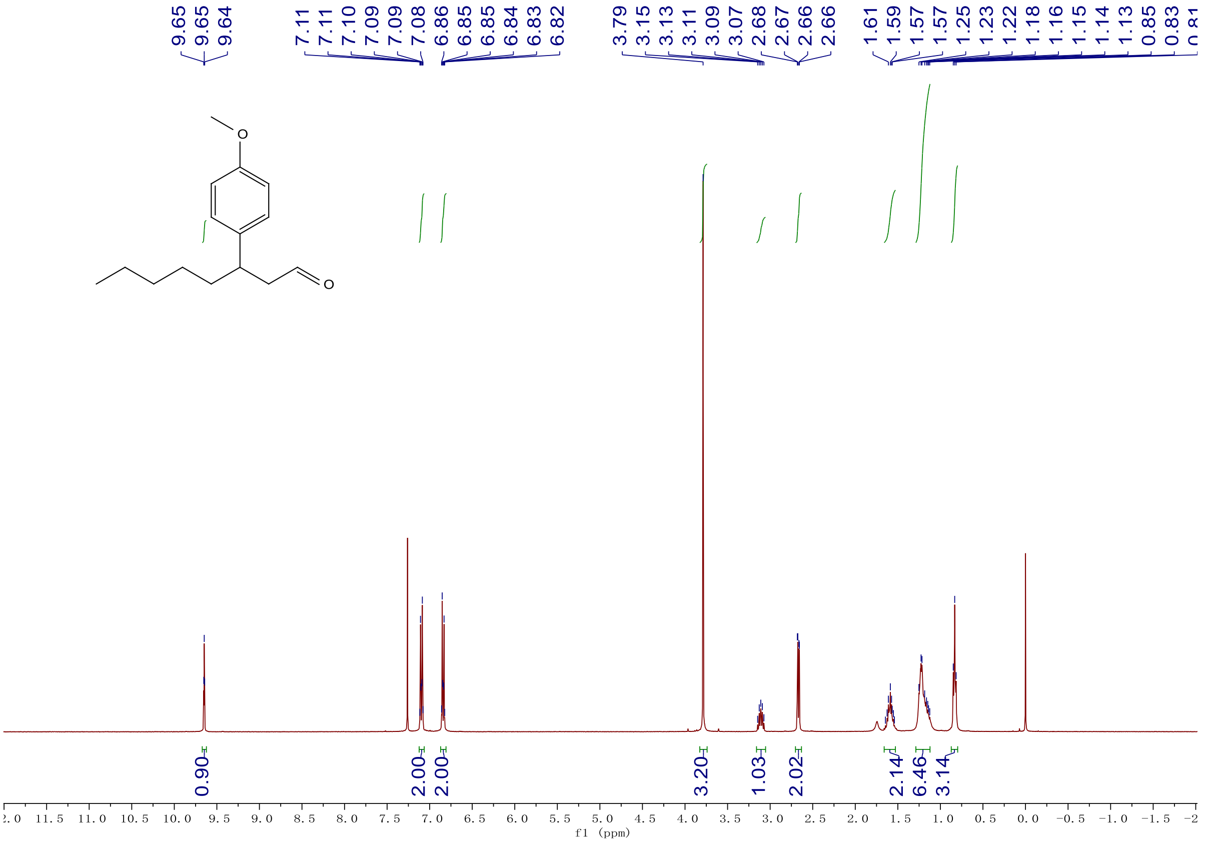

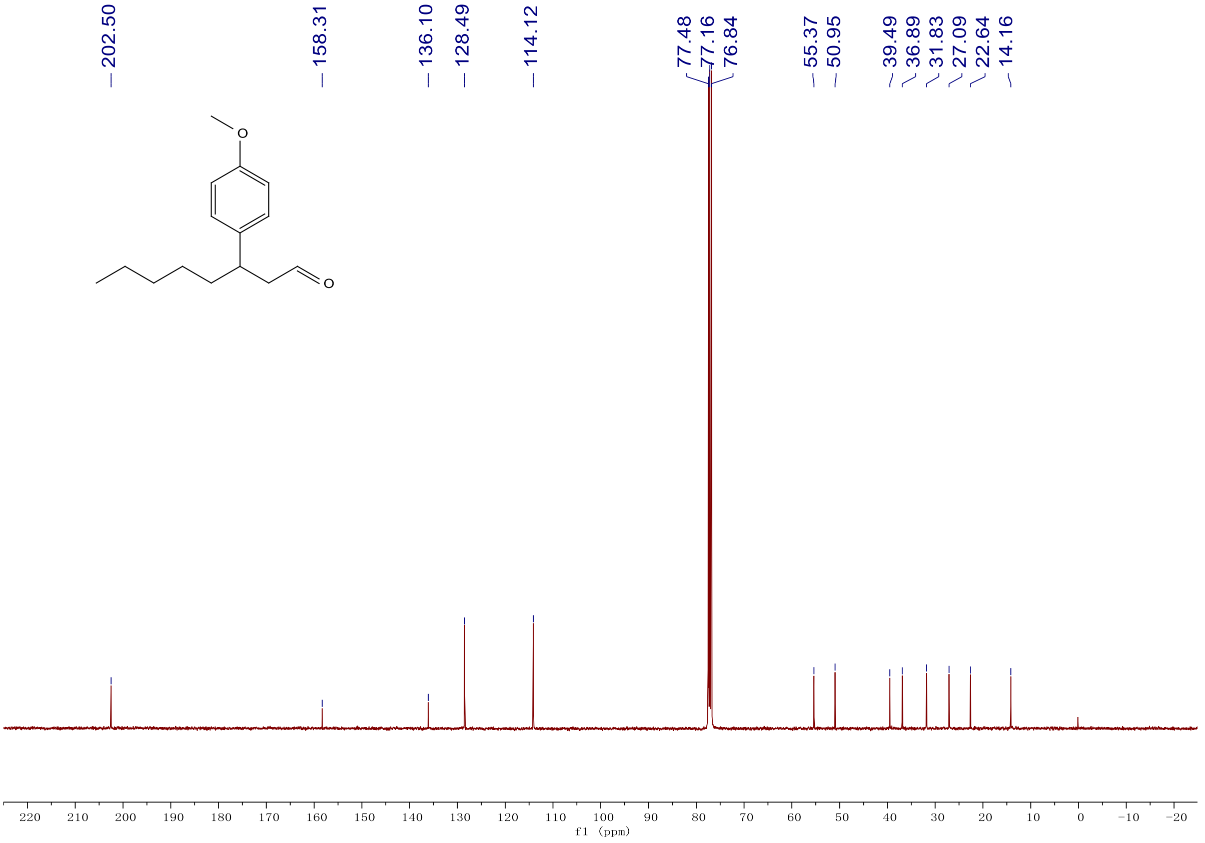

6-((*tert*-butyldimethylsilyl)oxy)-3-(4-methoxyphenyl)hexanal (3t)

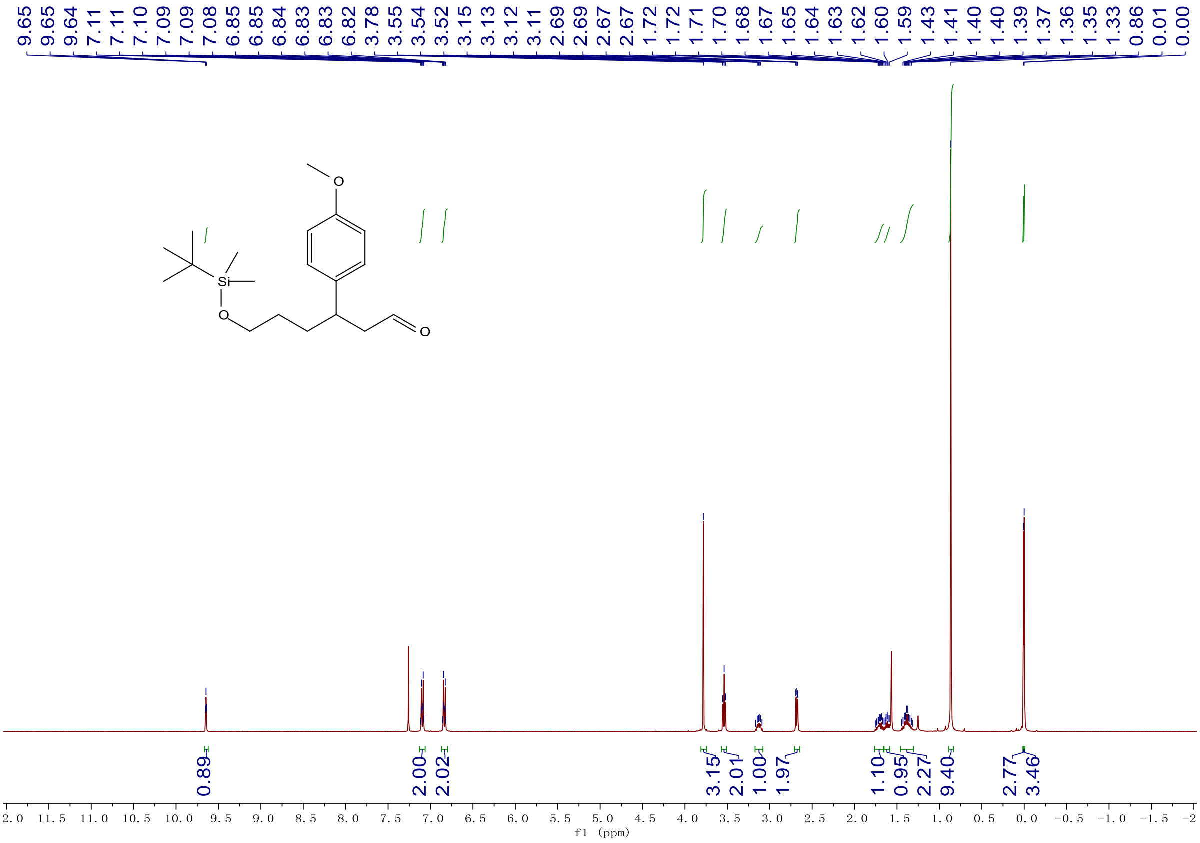

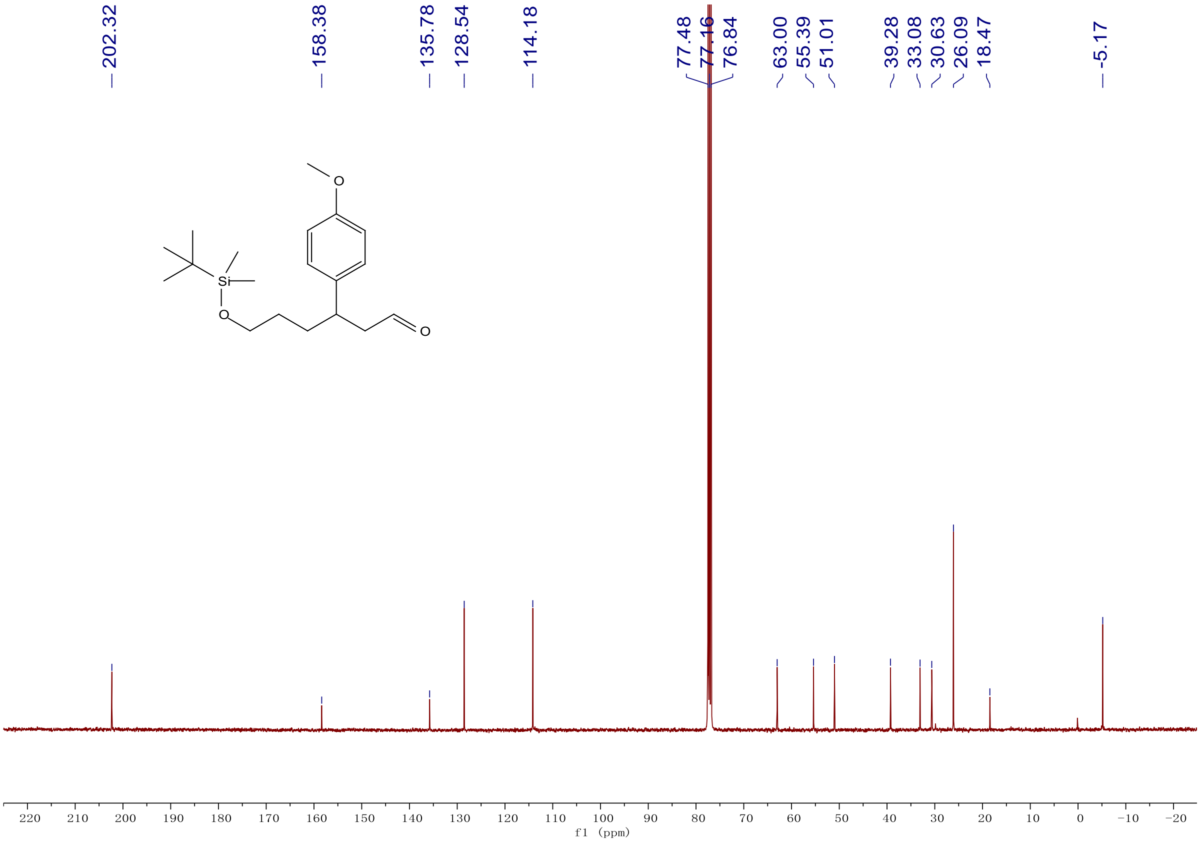

1-cyclohexyl-3-(4-methoxyphenyl)pentan-1-one (3u)

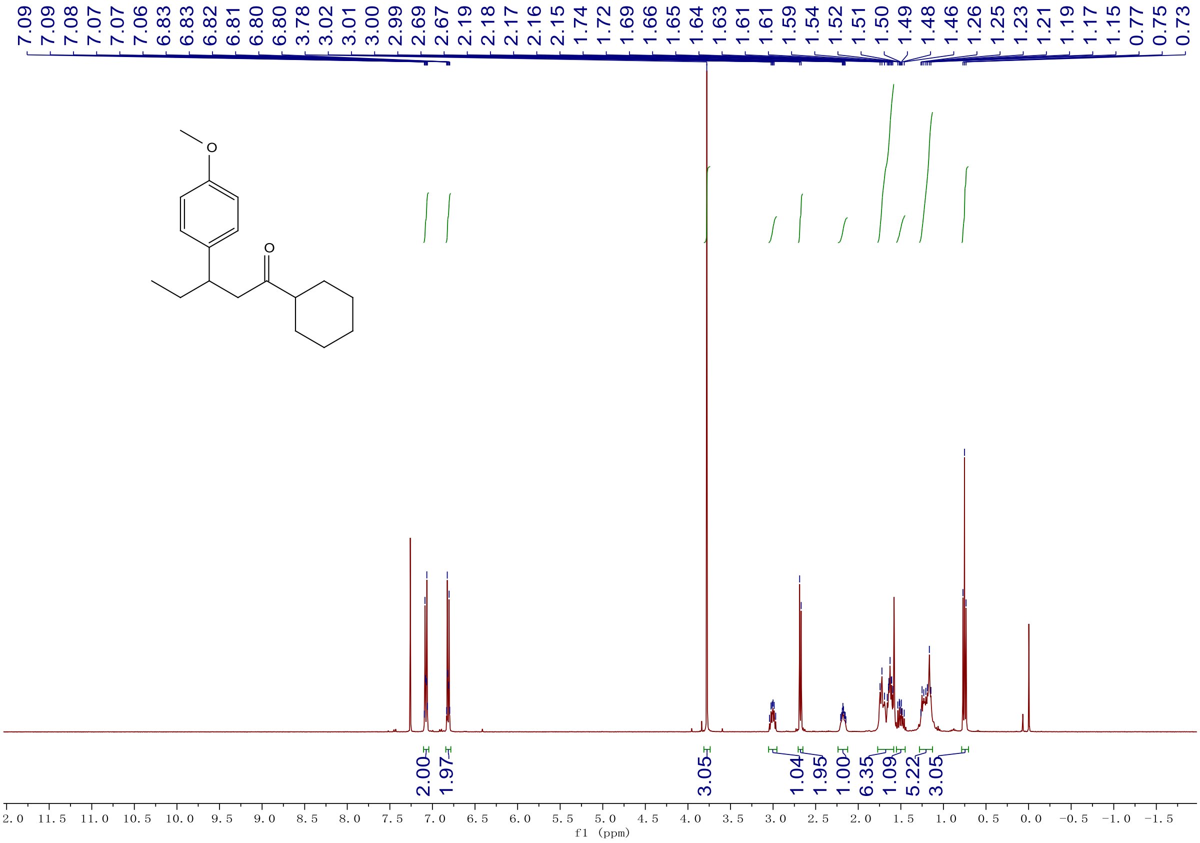

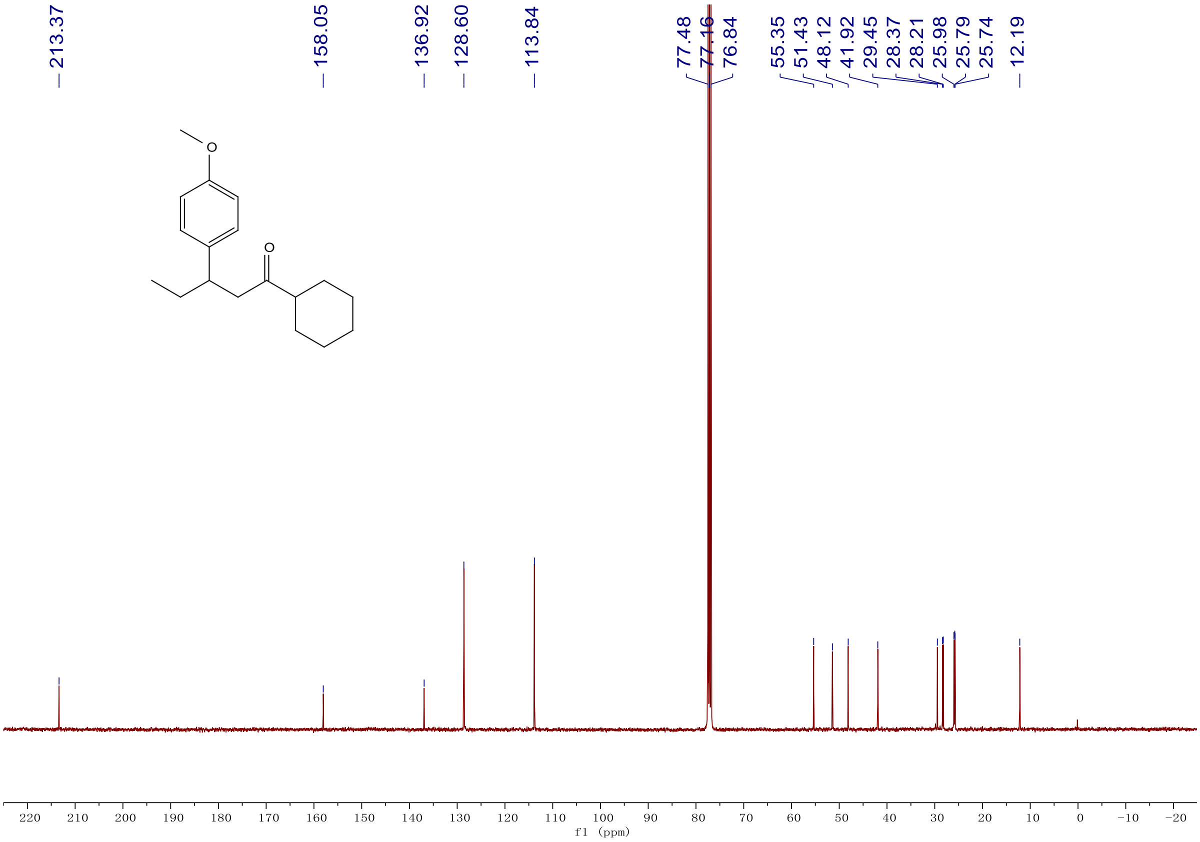

4-(4-methoxyphenyl)hexanal (3v)

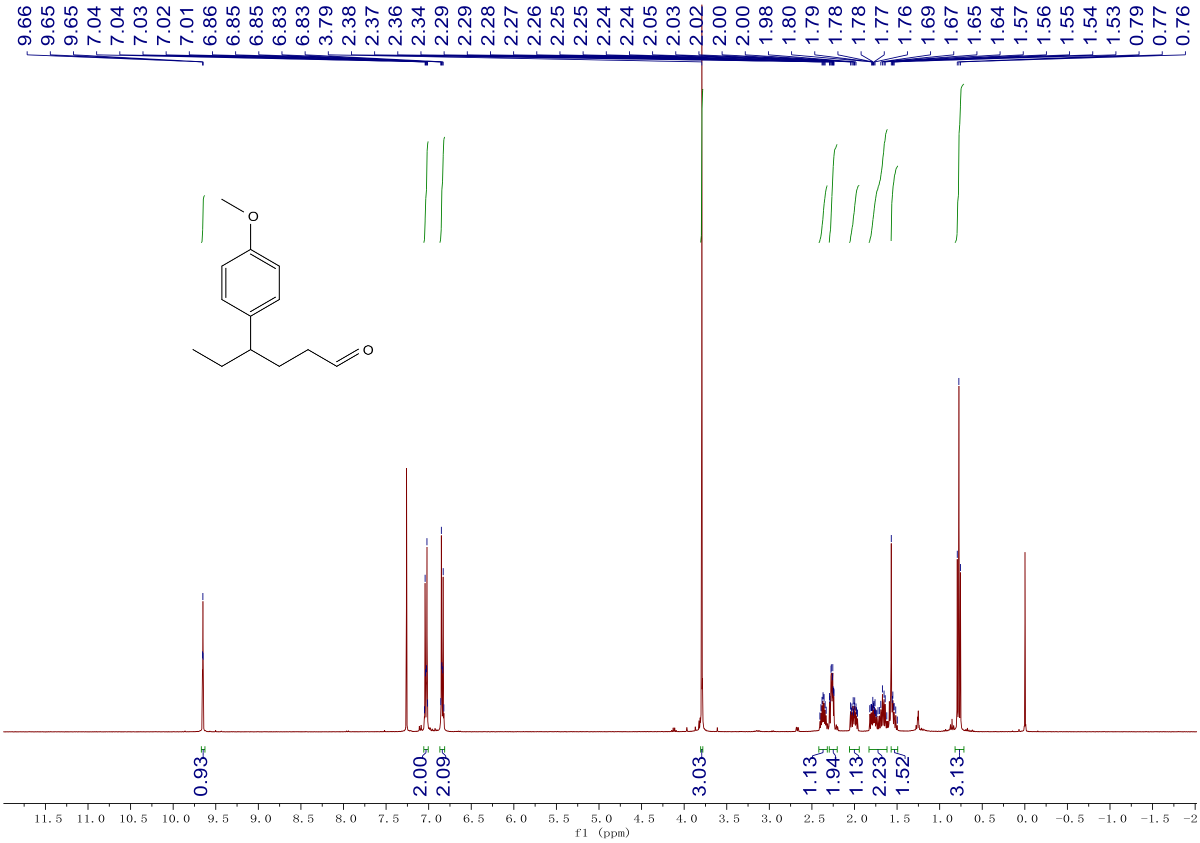

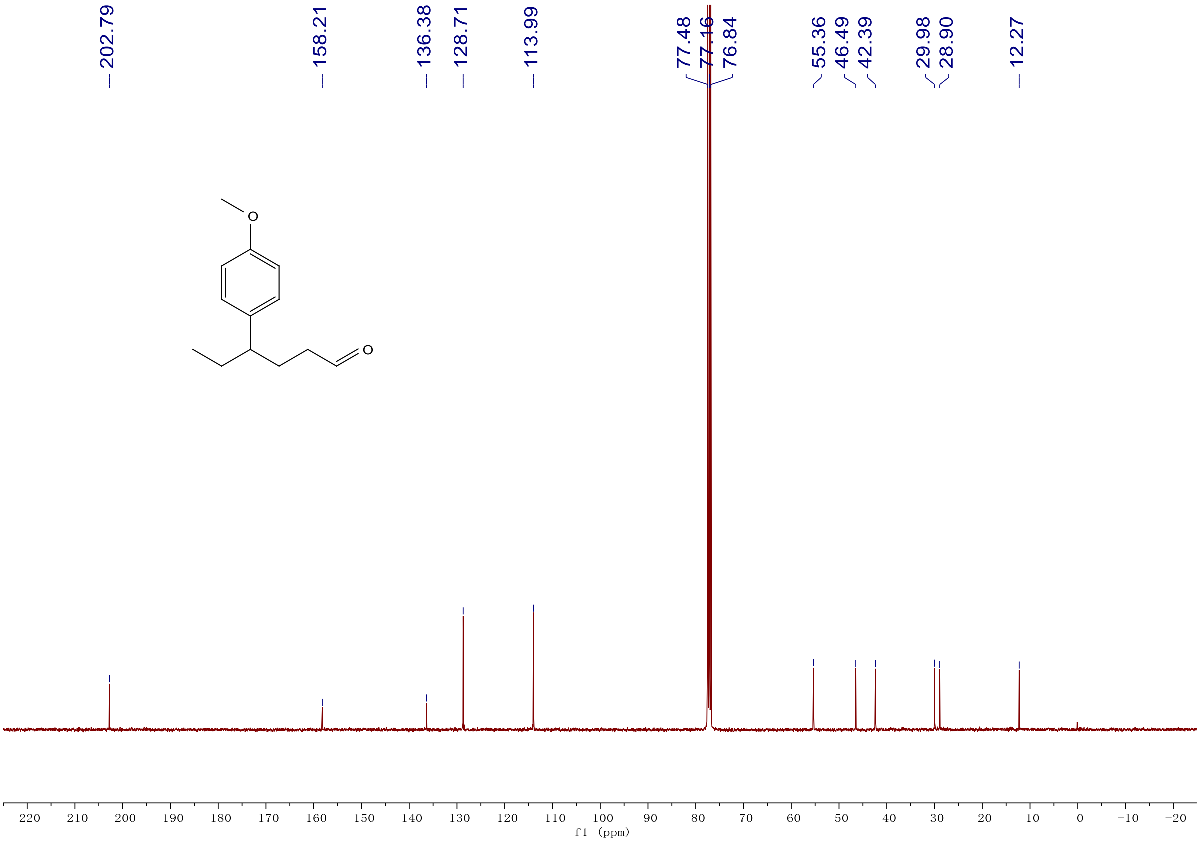

5-(4-methoxyphenyl)heptanal (3w)

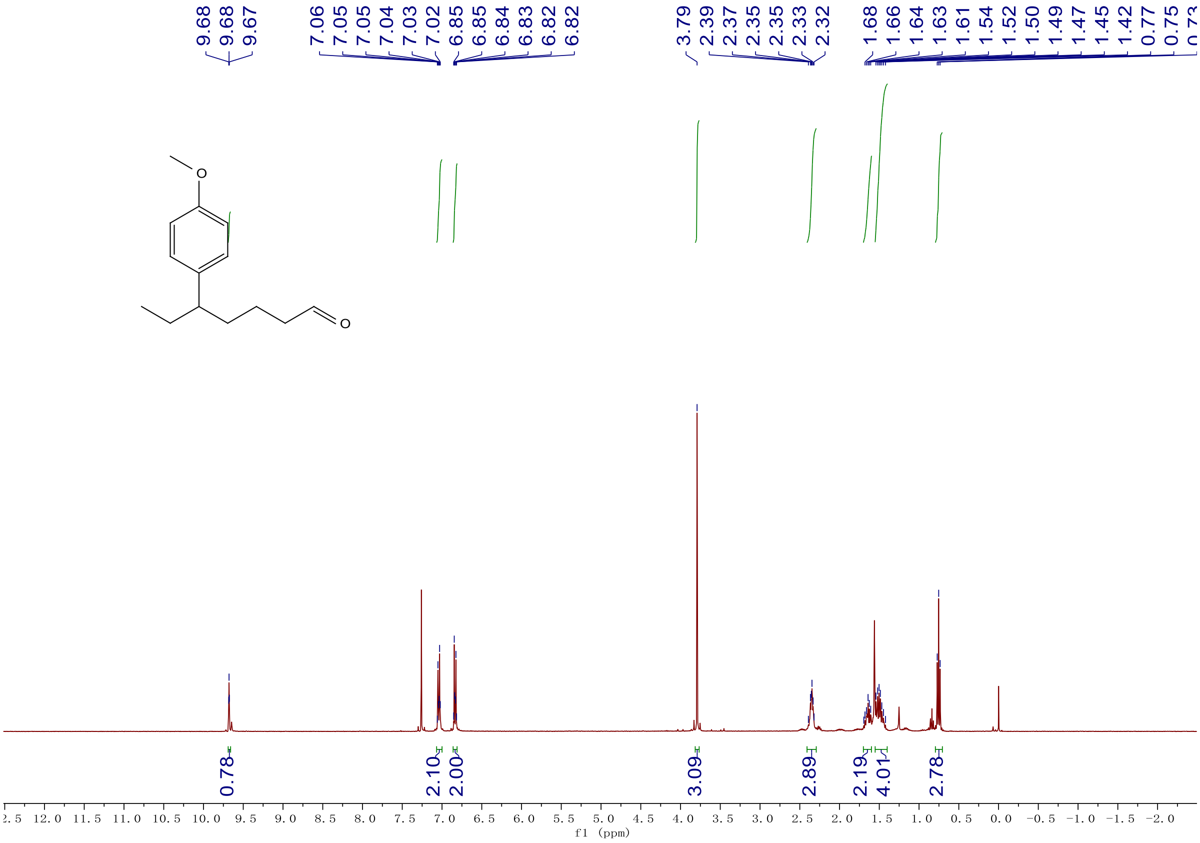

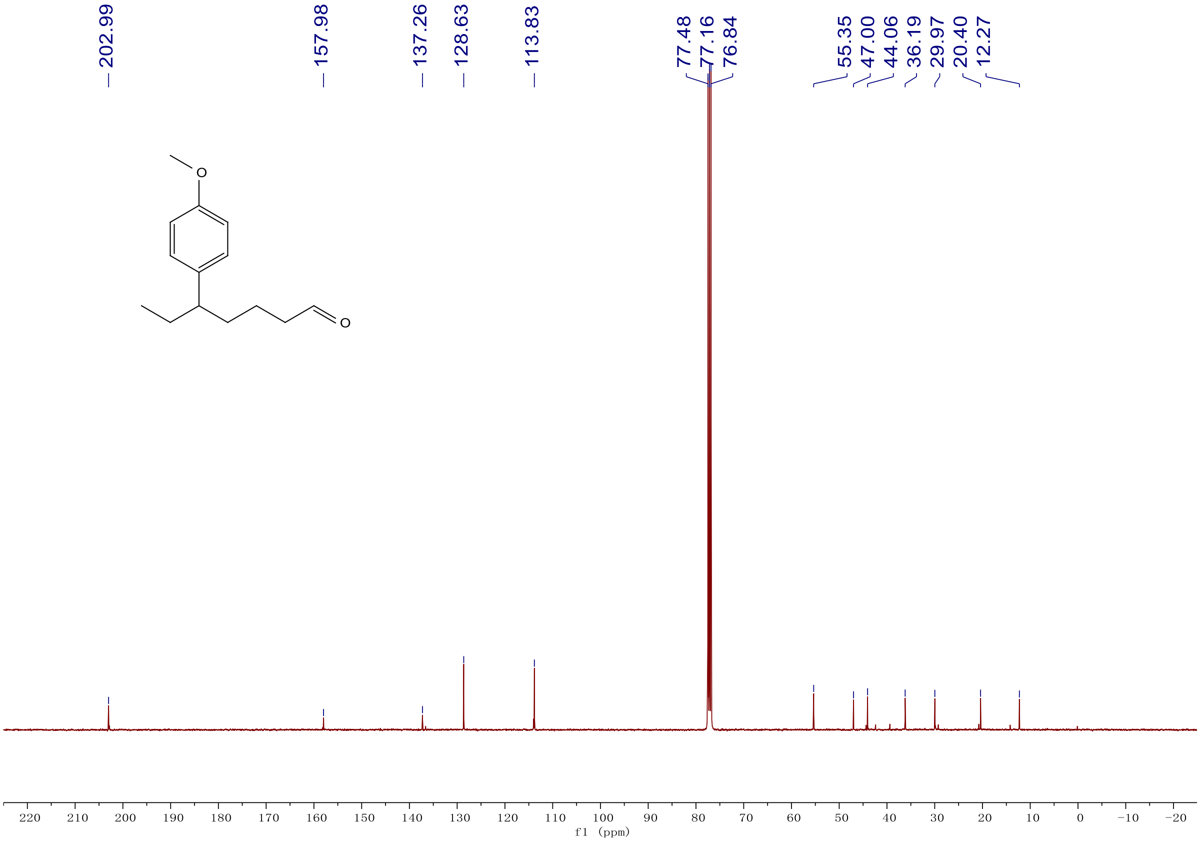

4-(4-(trifluoromethyl)phenyl)hexanal (3x)

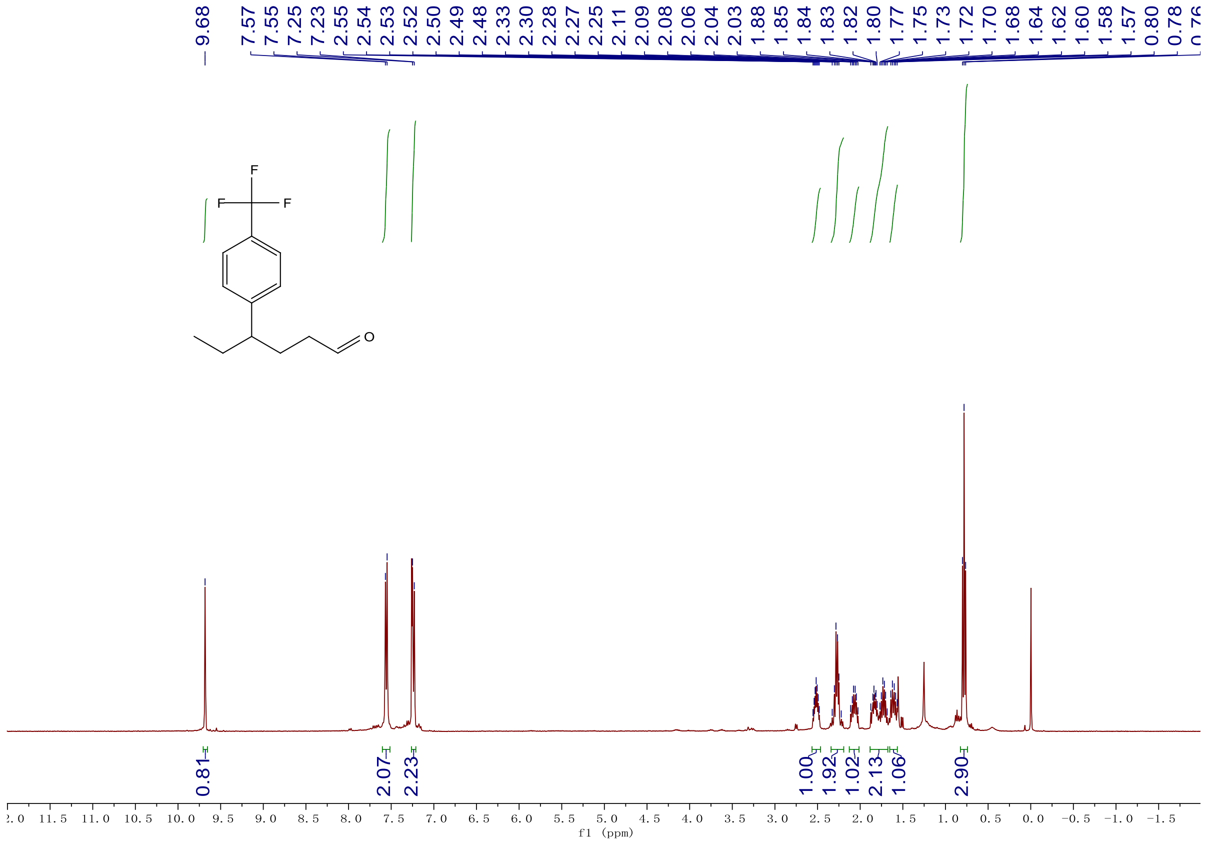

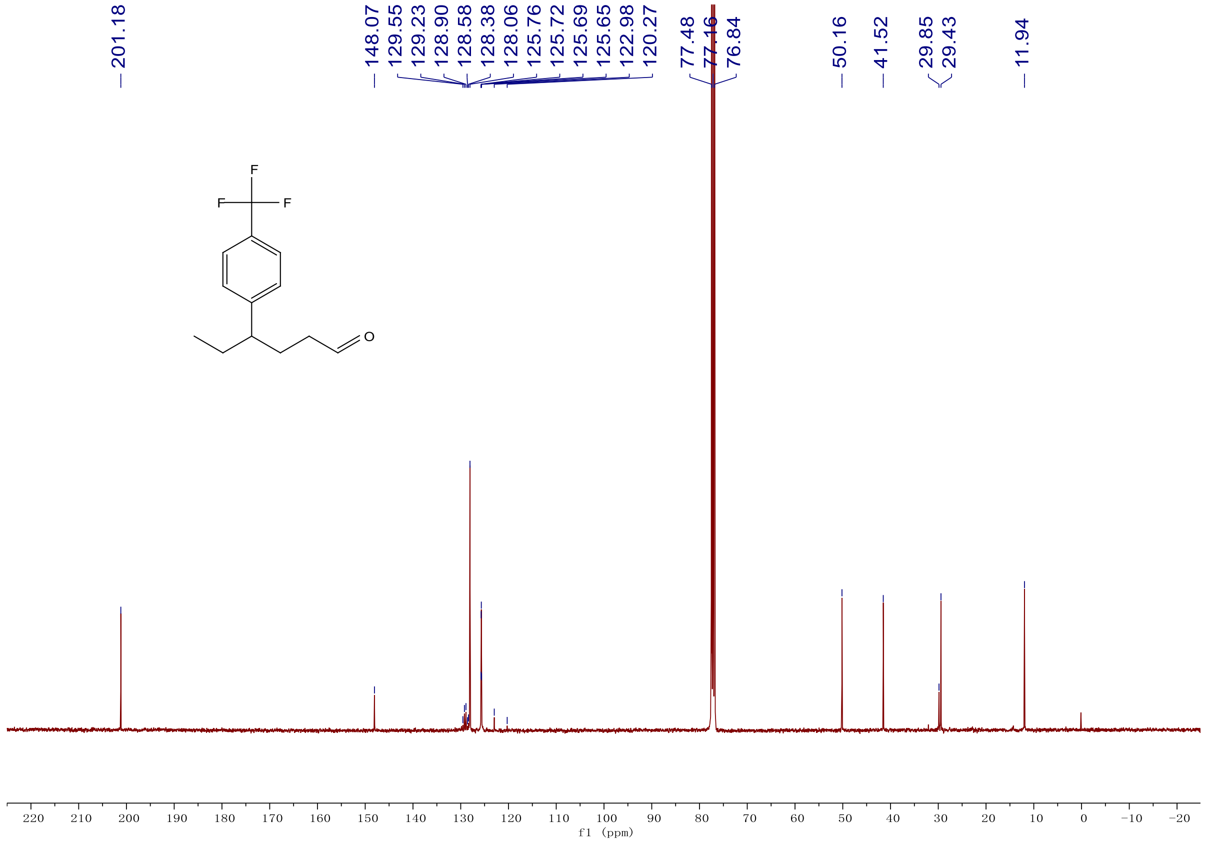

4-(4-(benzyloxy)phenyl)hexanal (3y)

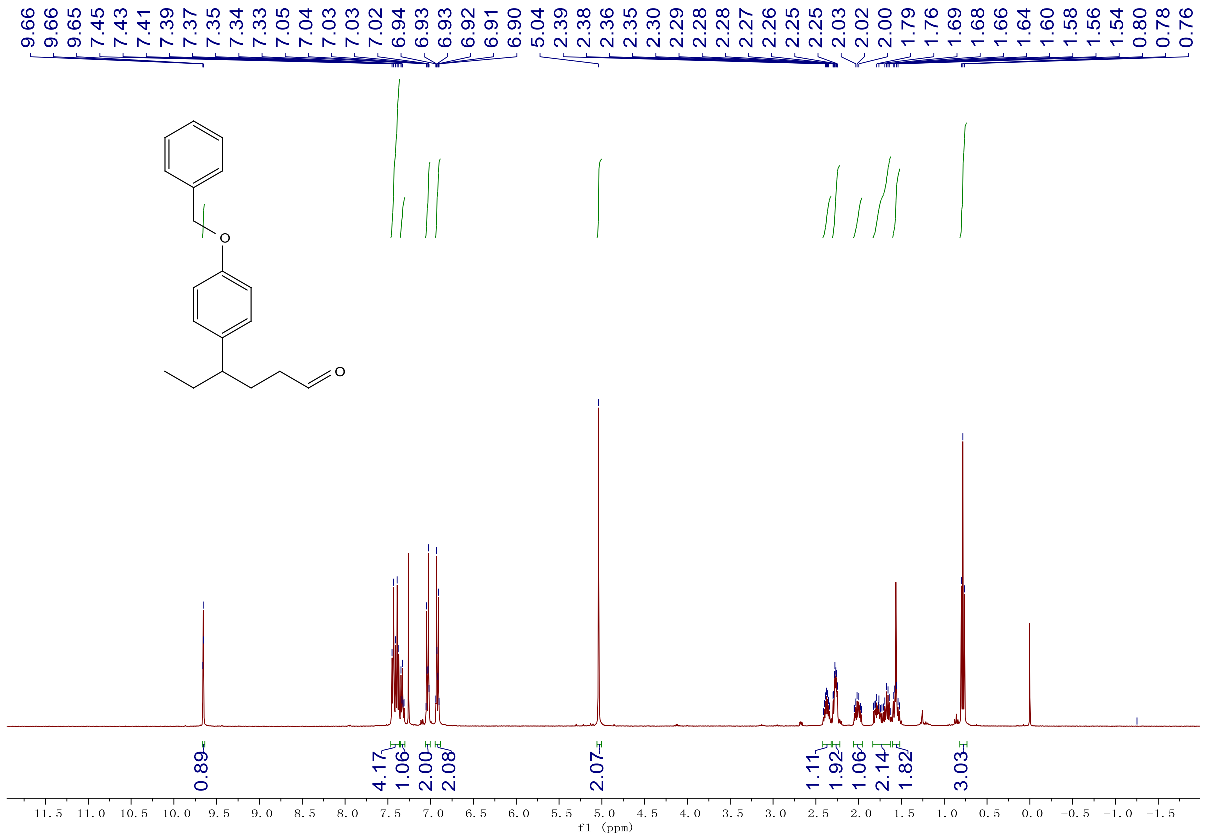

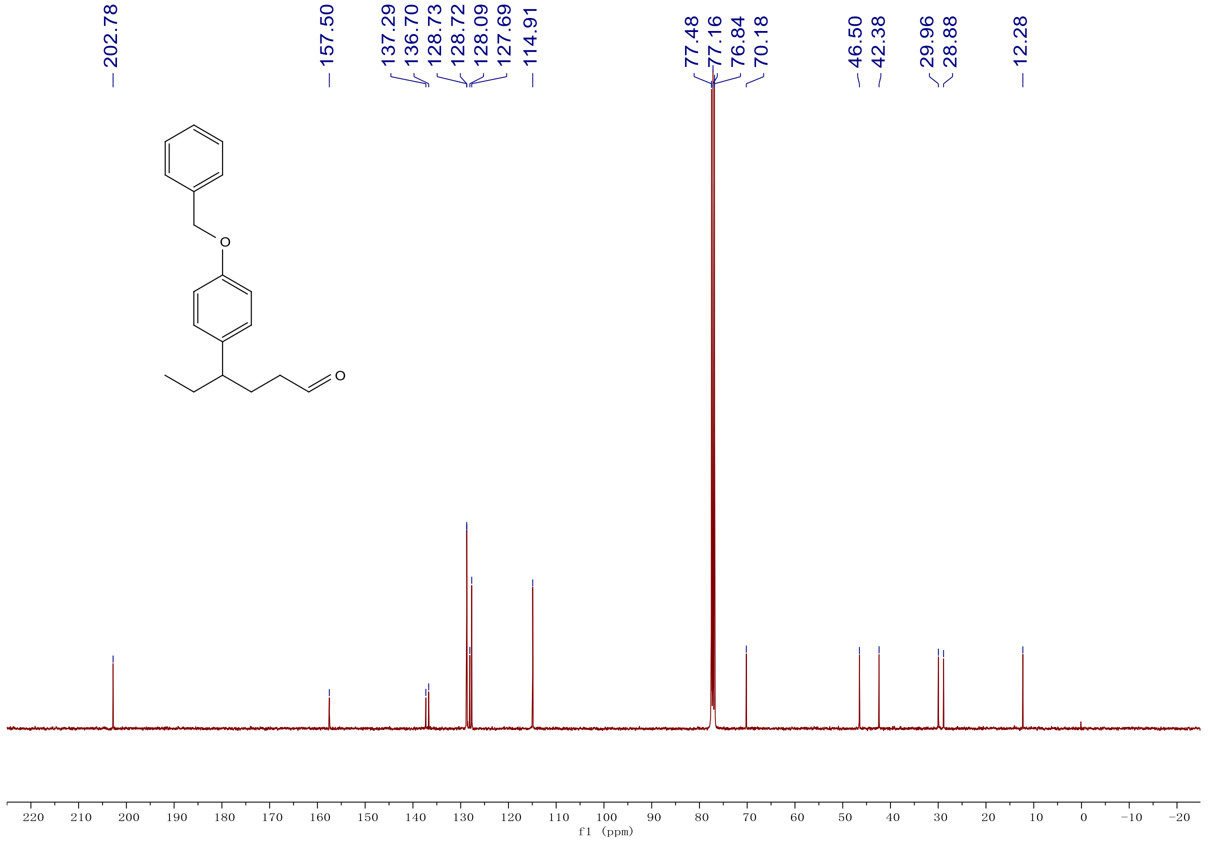

4-([1,1'-biphenyl]-4-yl)hexanal (3z)

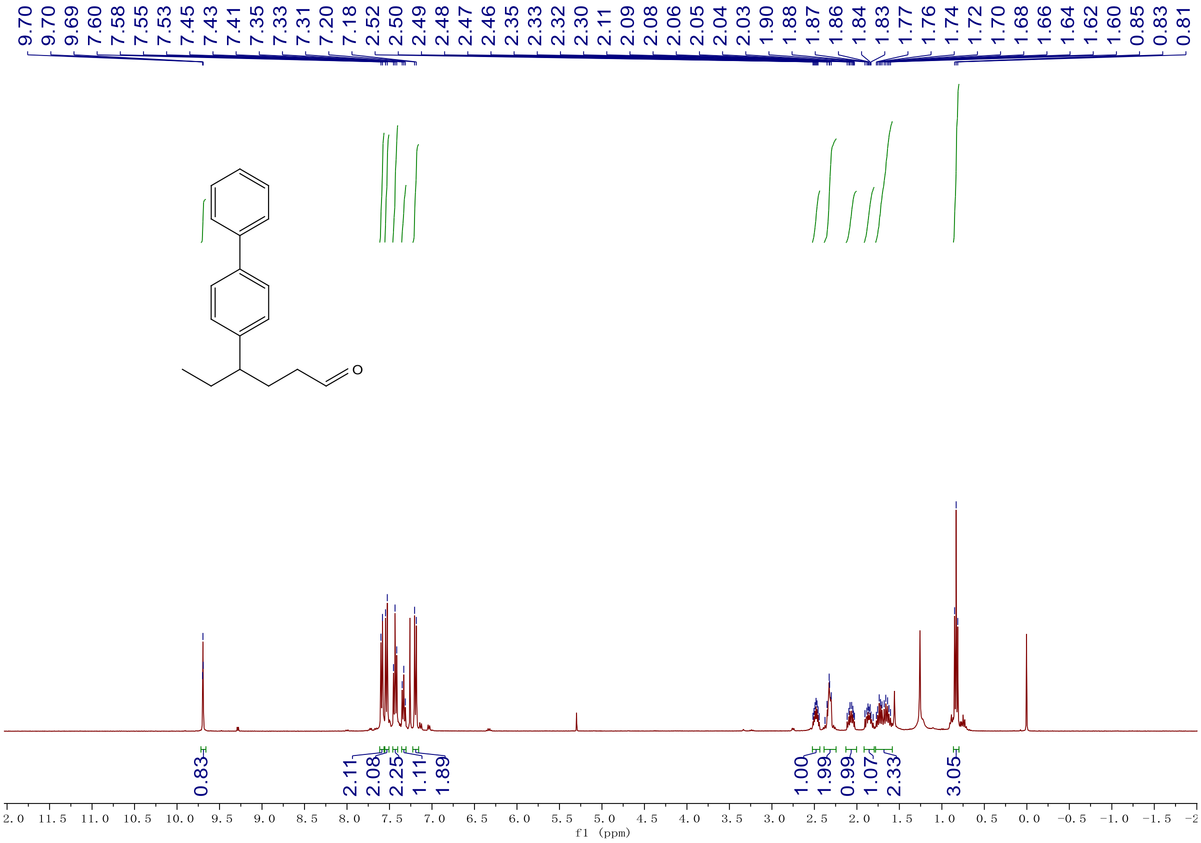

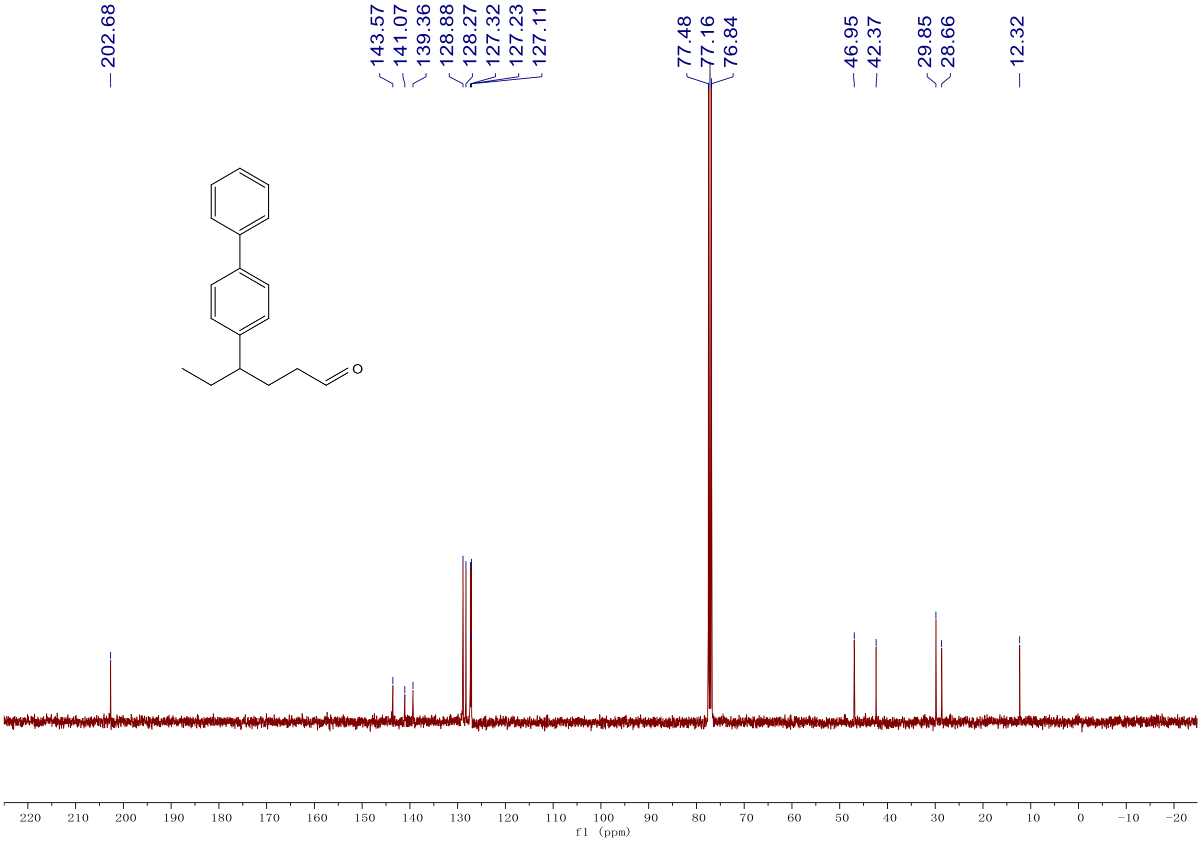

4-(3-fluorophenyl)hexanal (3aa)

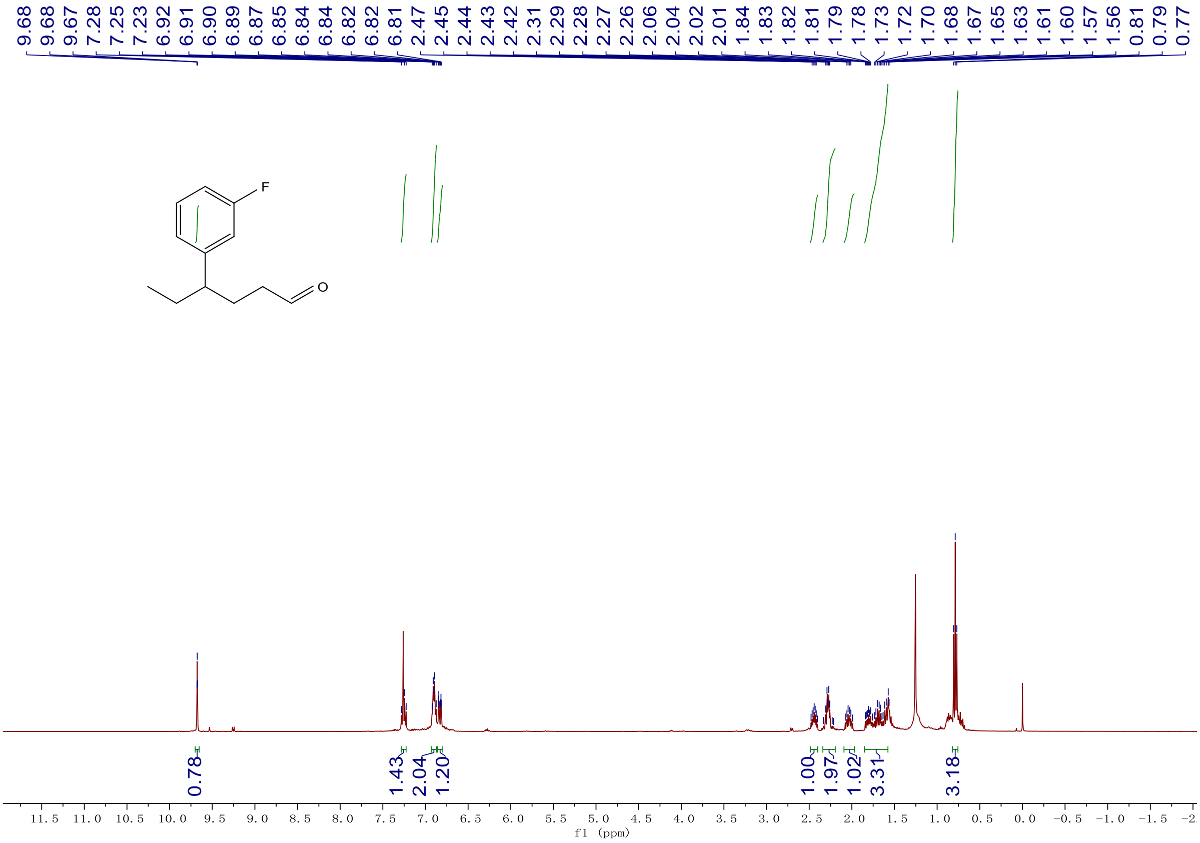

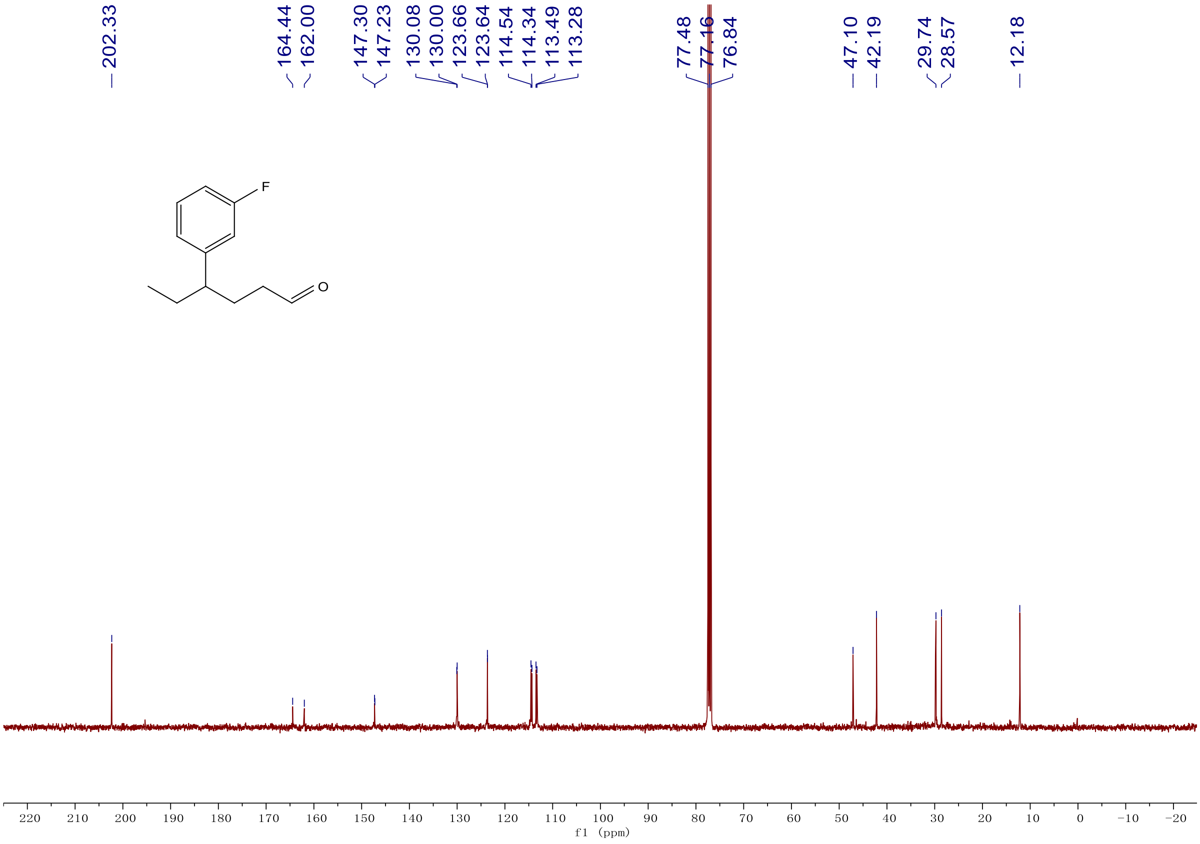

4-(2-fluorophenyl)hexanal (3ab)

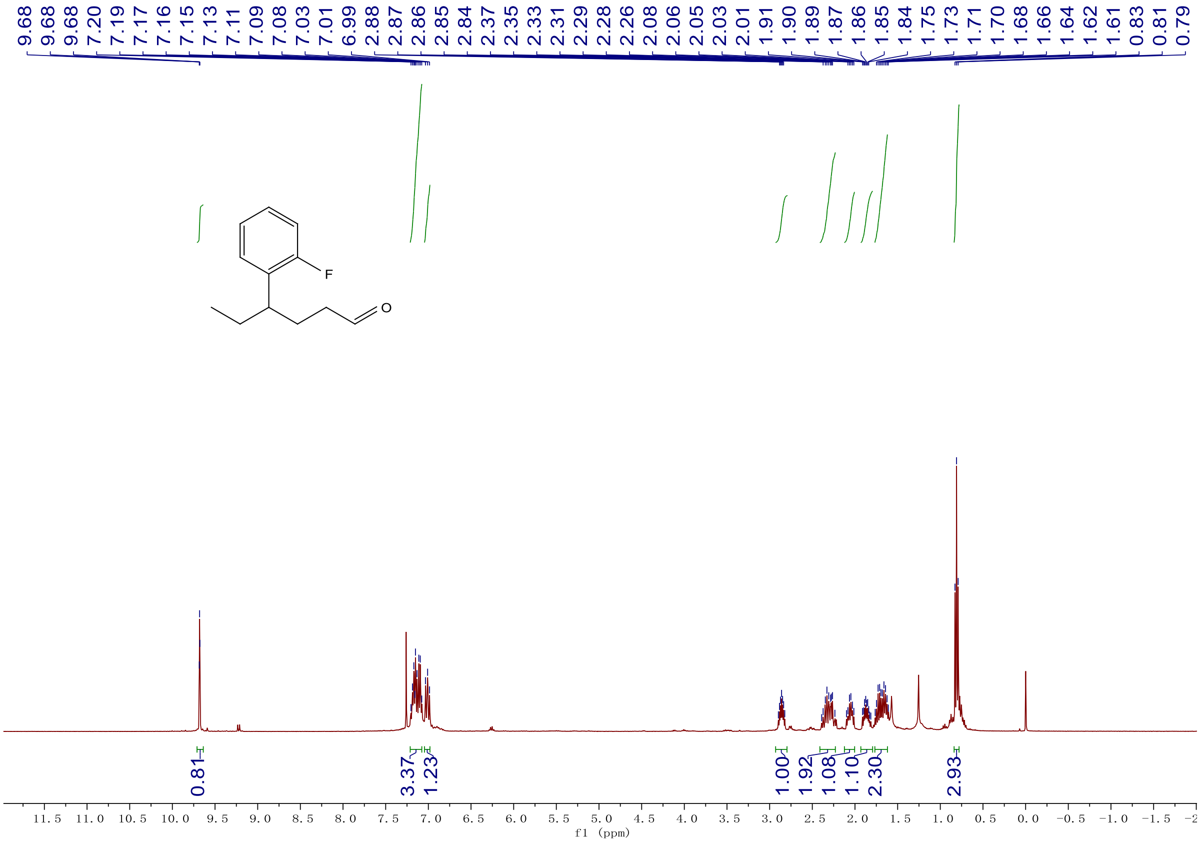

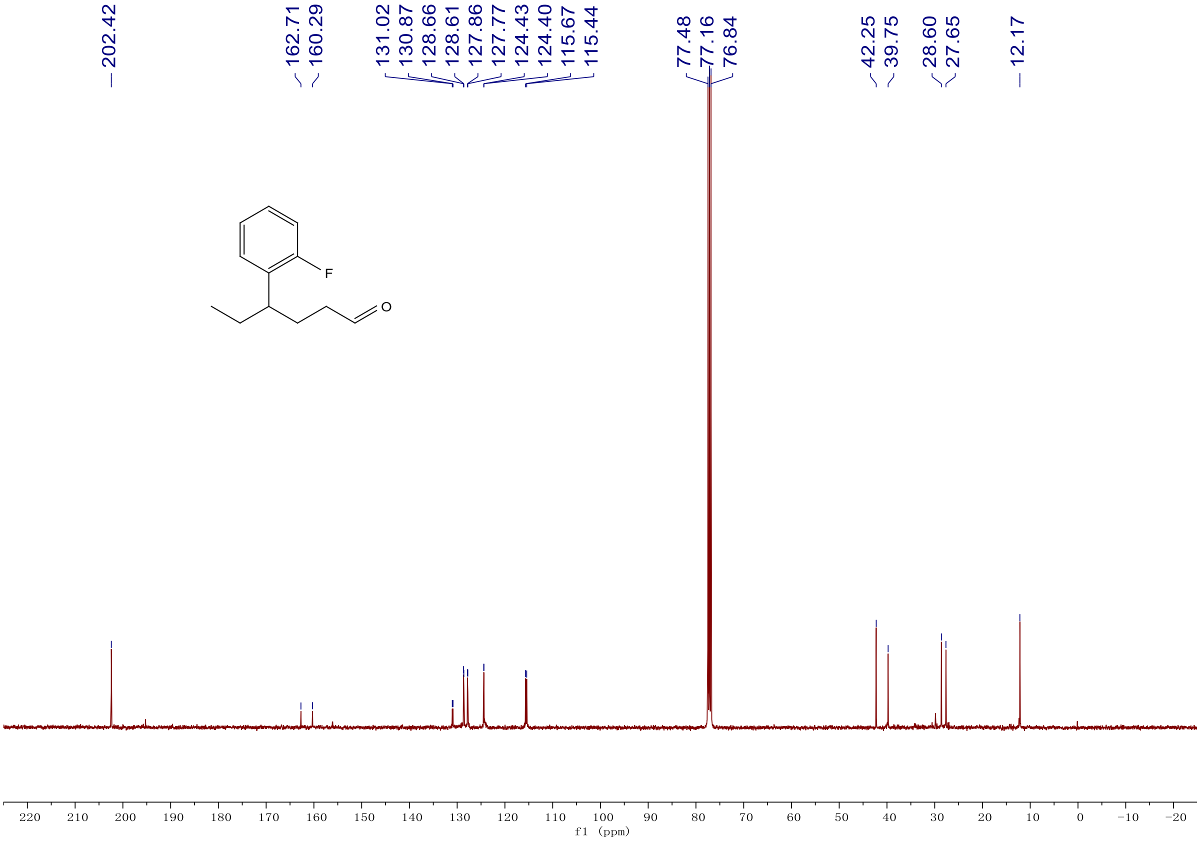

4-(*o*-tolyl)hexanal (3ac)

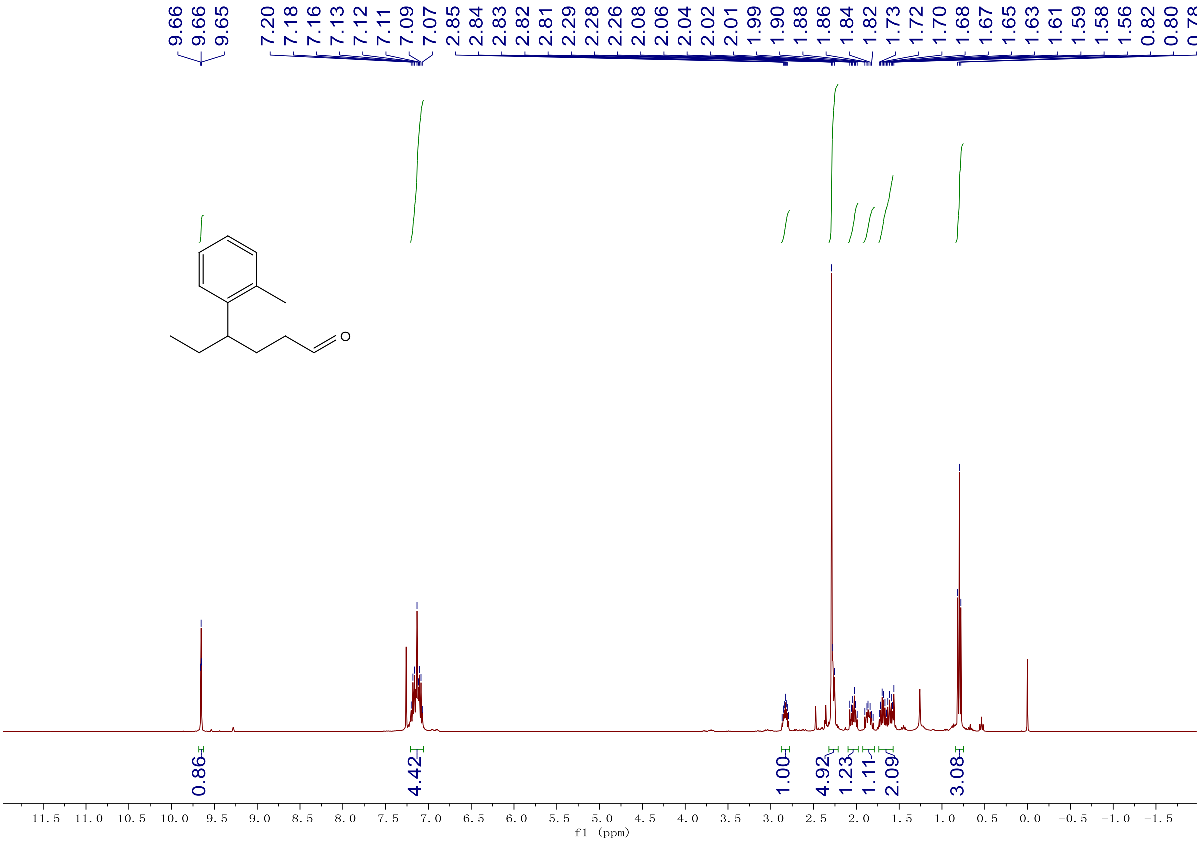

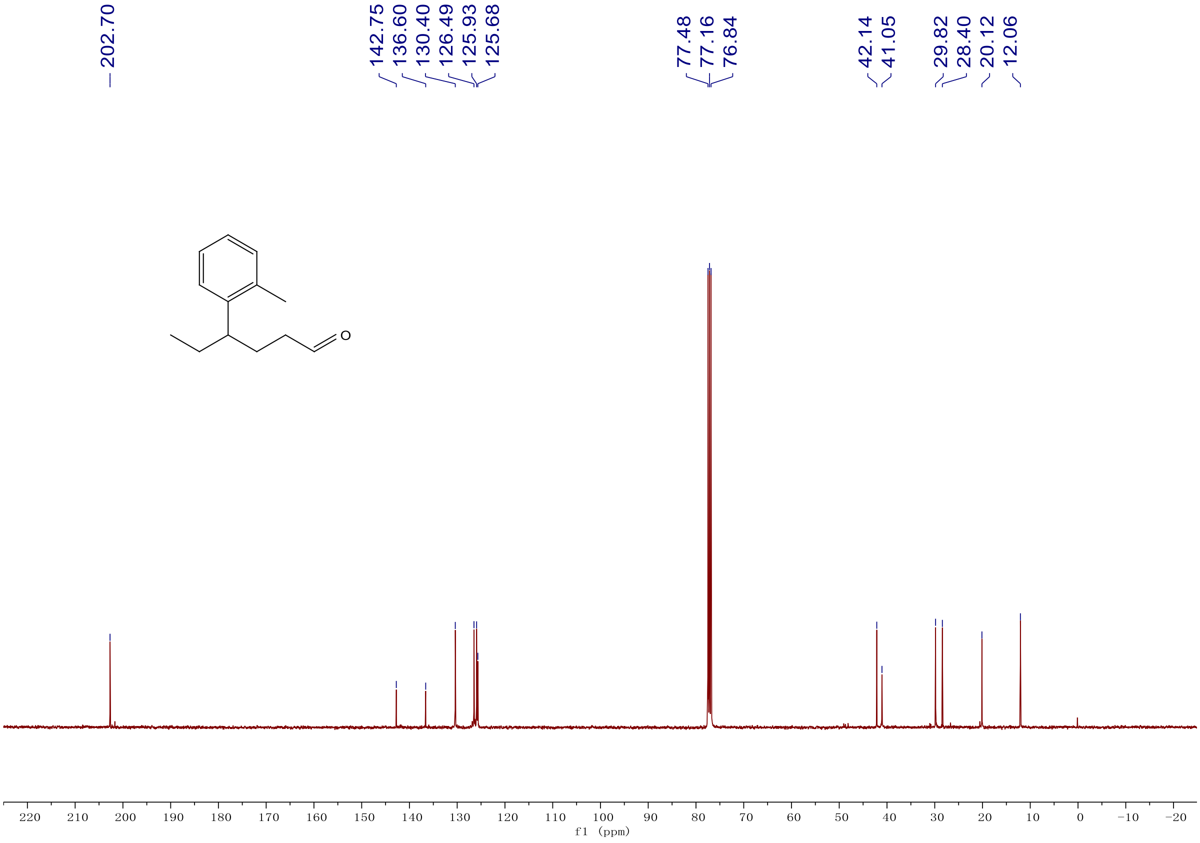

4-(naphthalen-1-yl)hexanal (3ad)

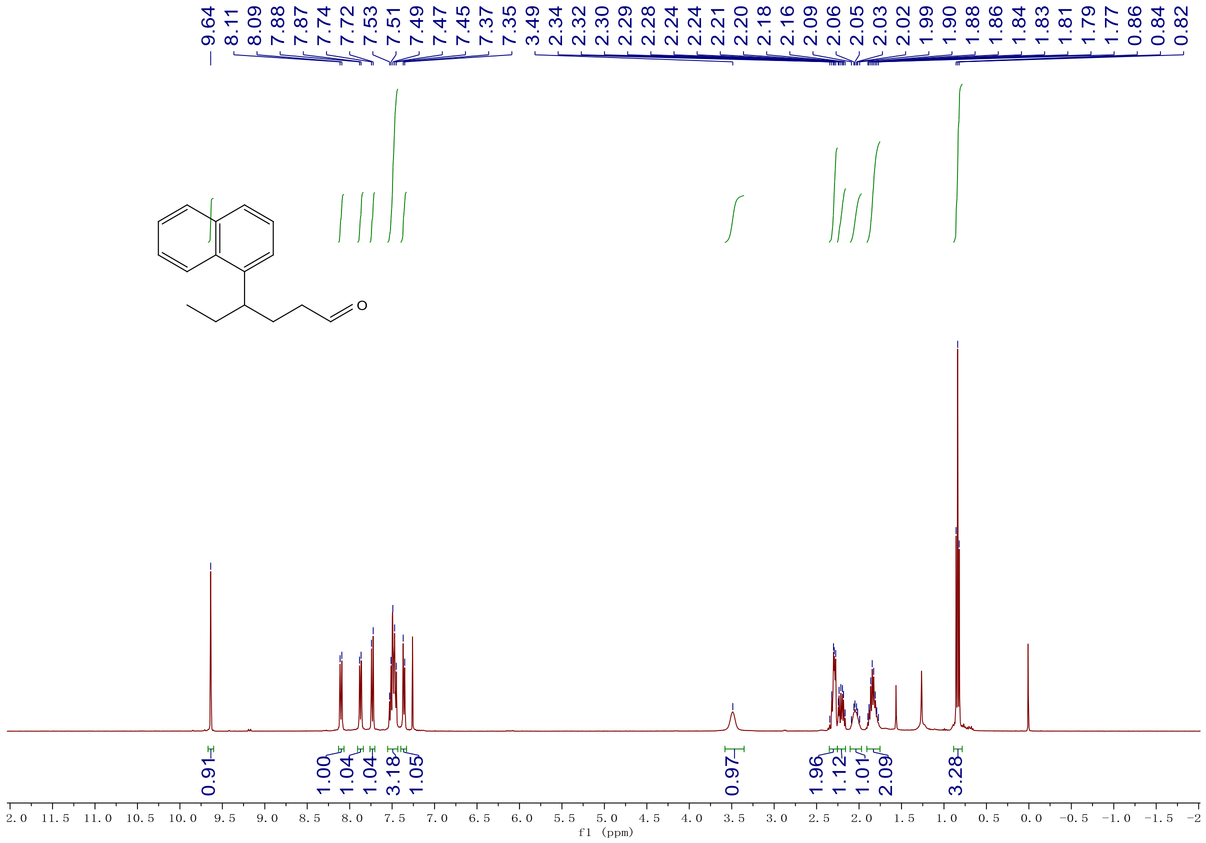

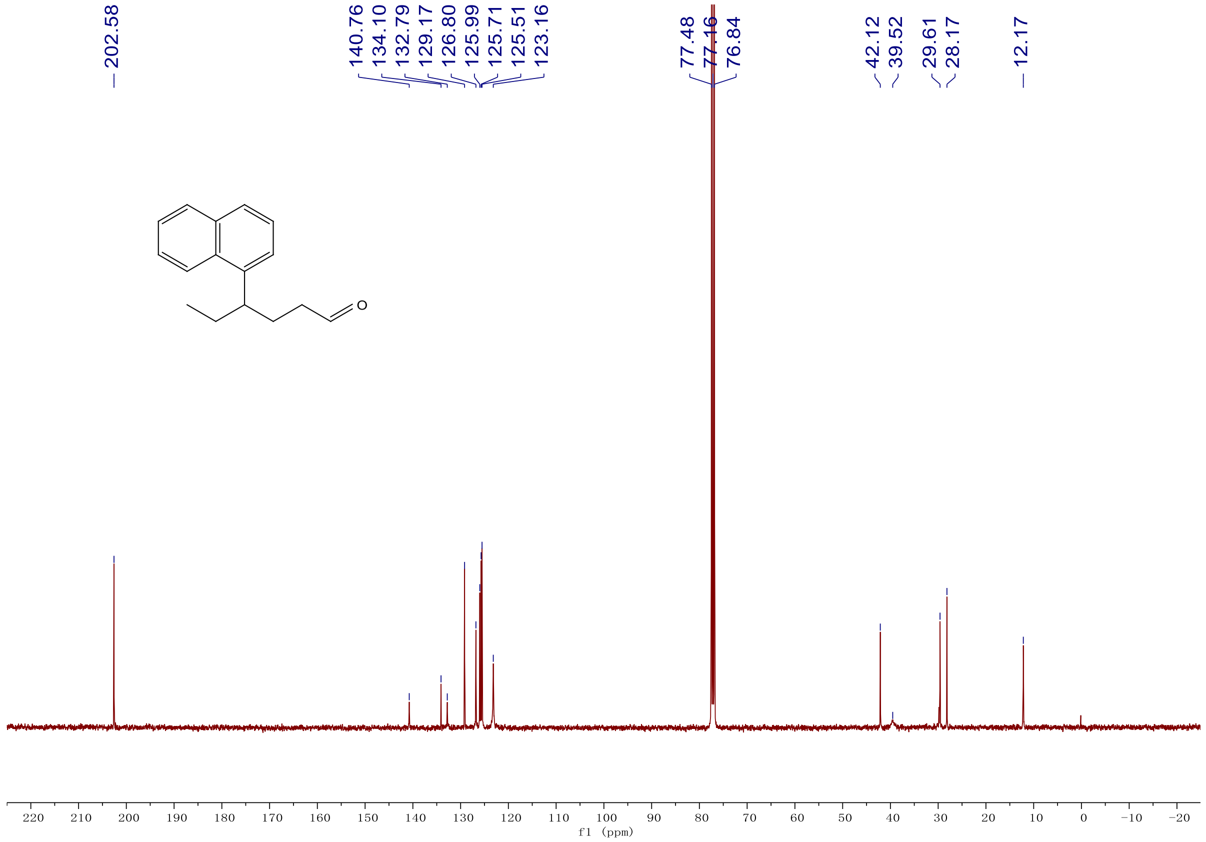

3-(4-methoxyphenyl)-3-phenylpropanal (4a)

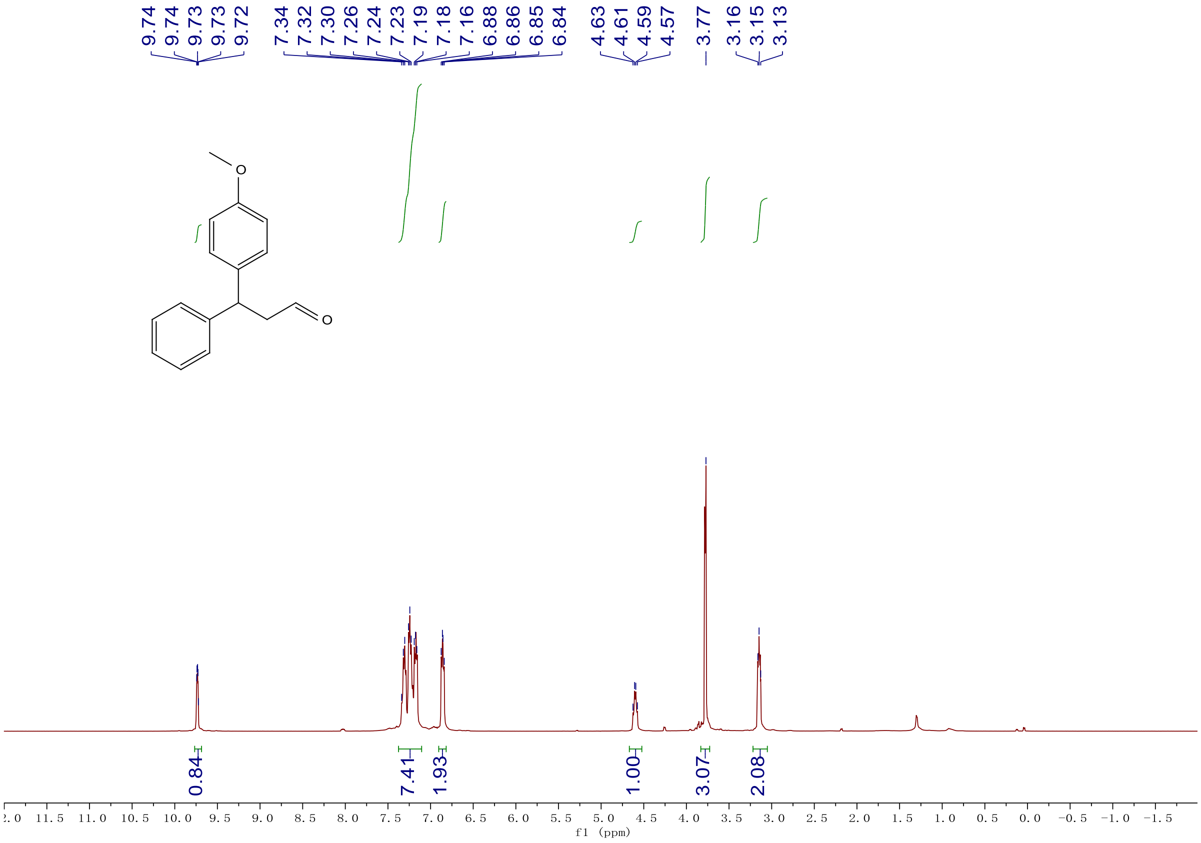

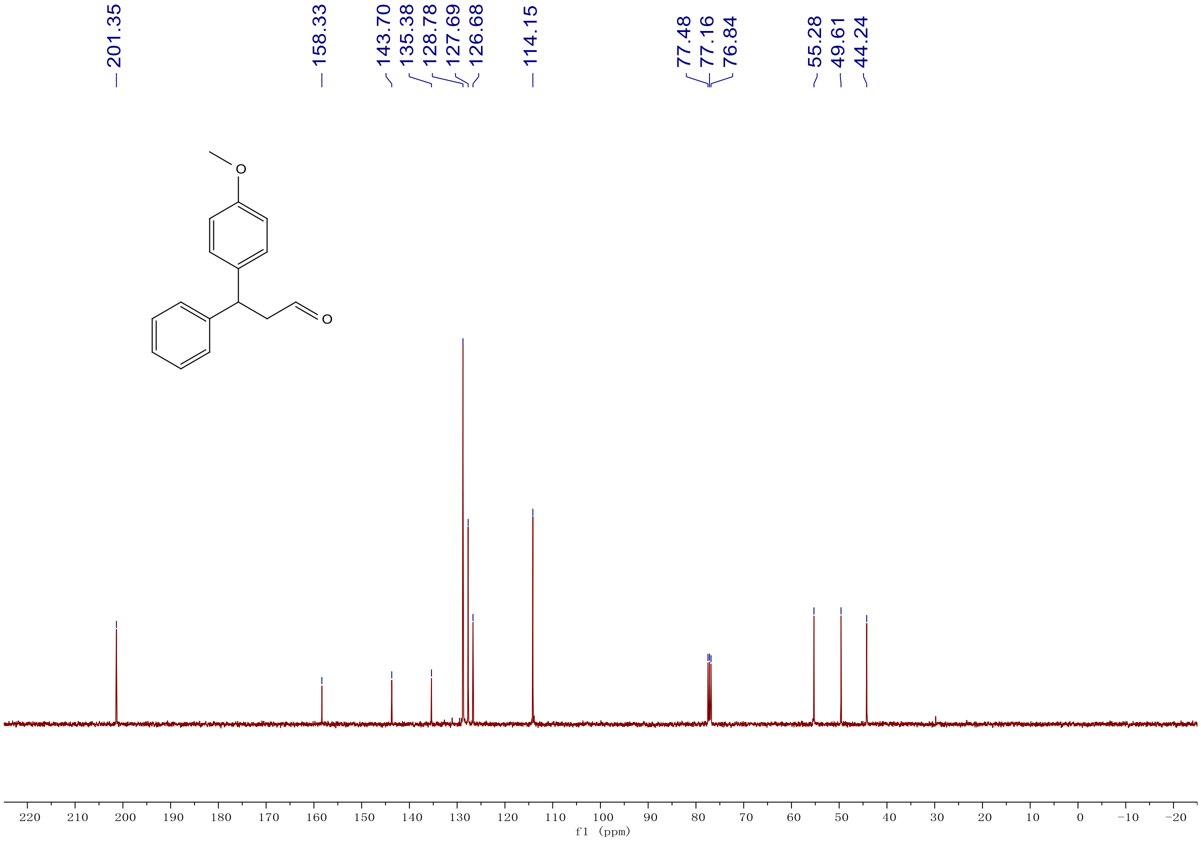

3-([1,1'-biphenyl]-4-yl)-3-phenylpropanal (4b)

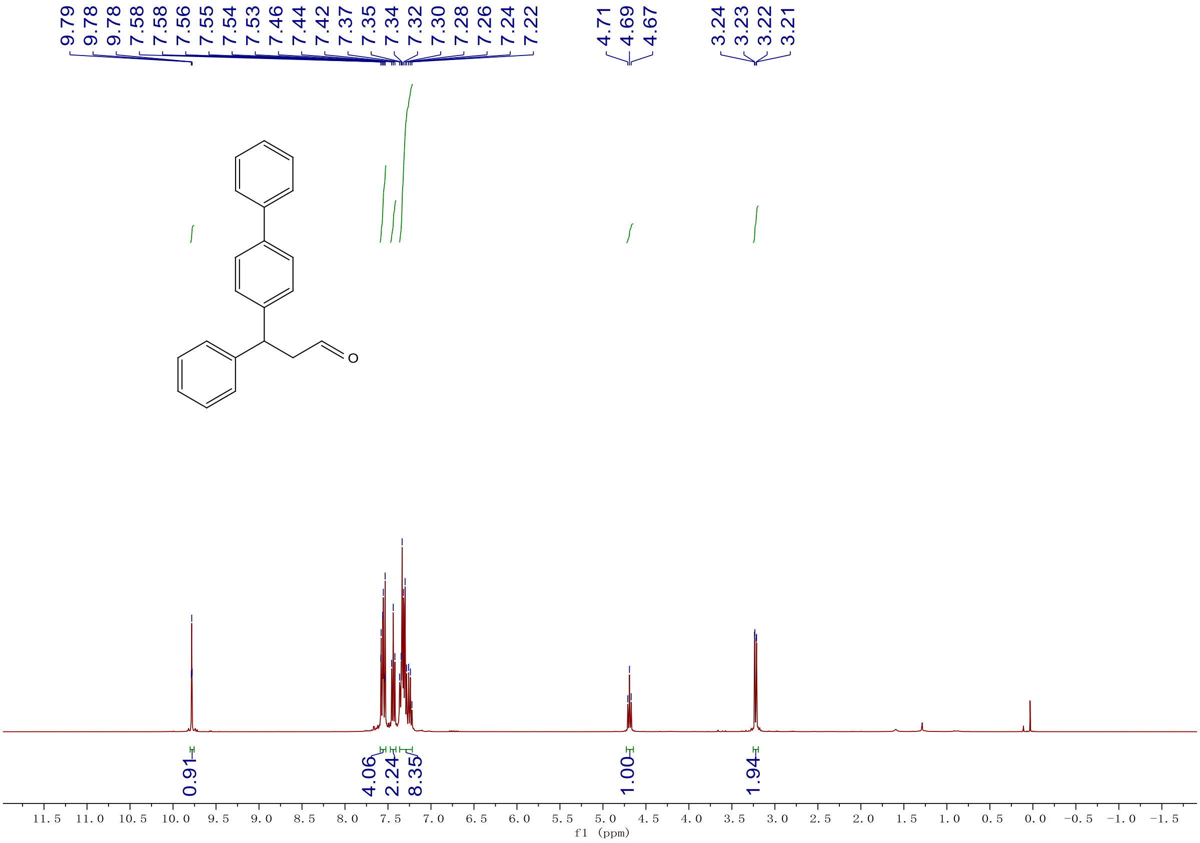

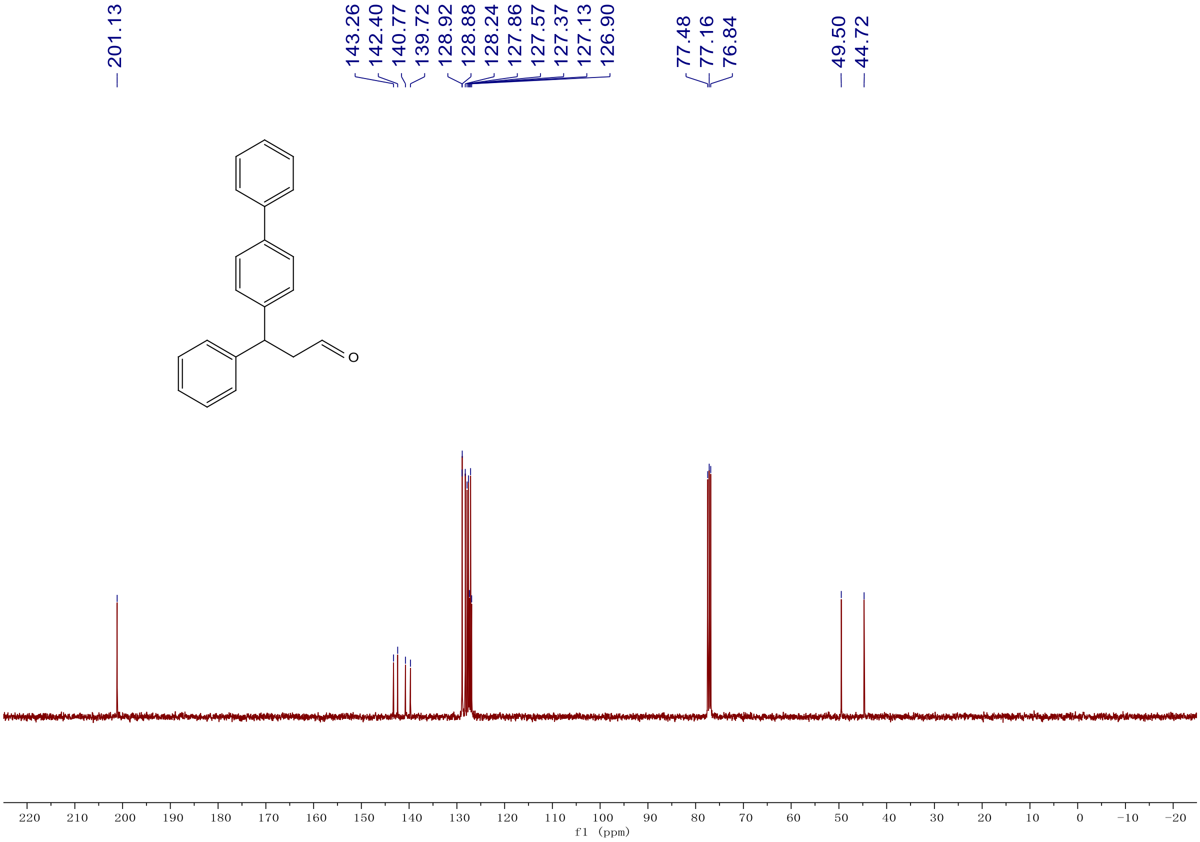

3-(3-chlorophenyl)-3-phenylpropanal (4c)

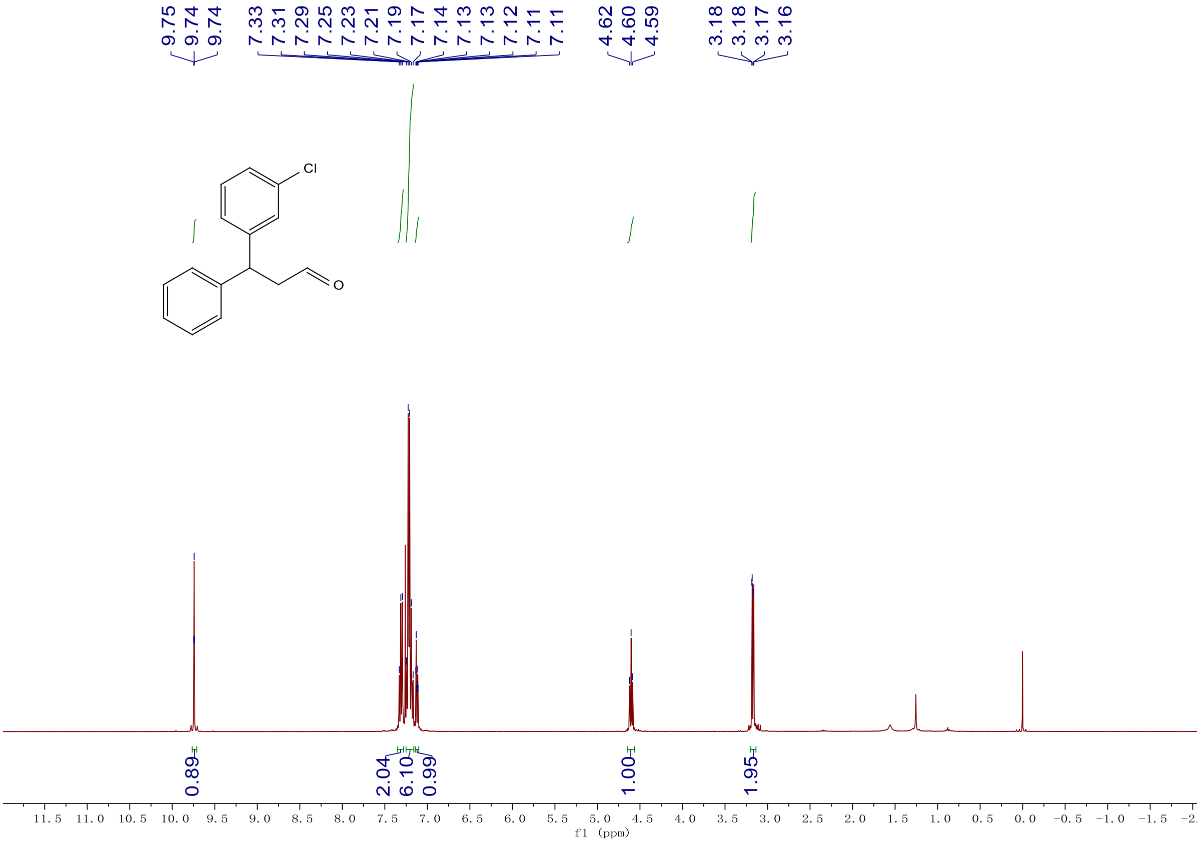

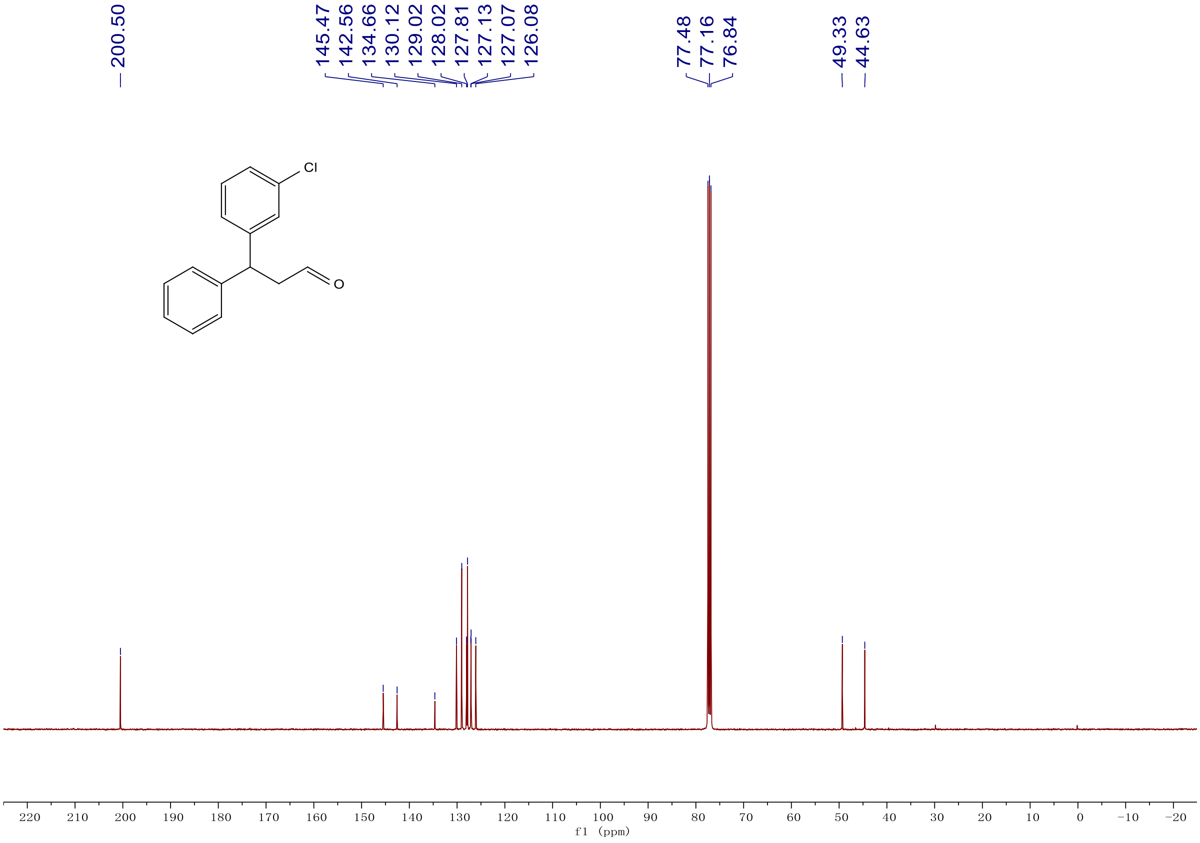

3-(2-fluorophenyl)-3-phenylpropanal (4d)

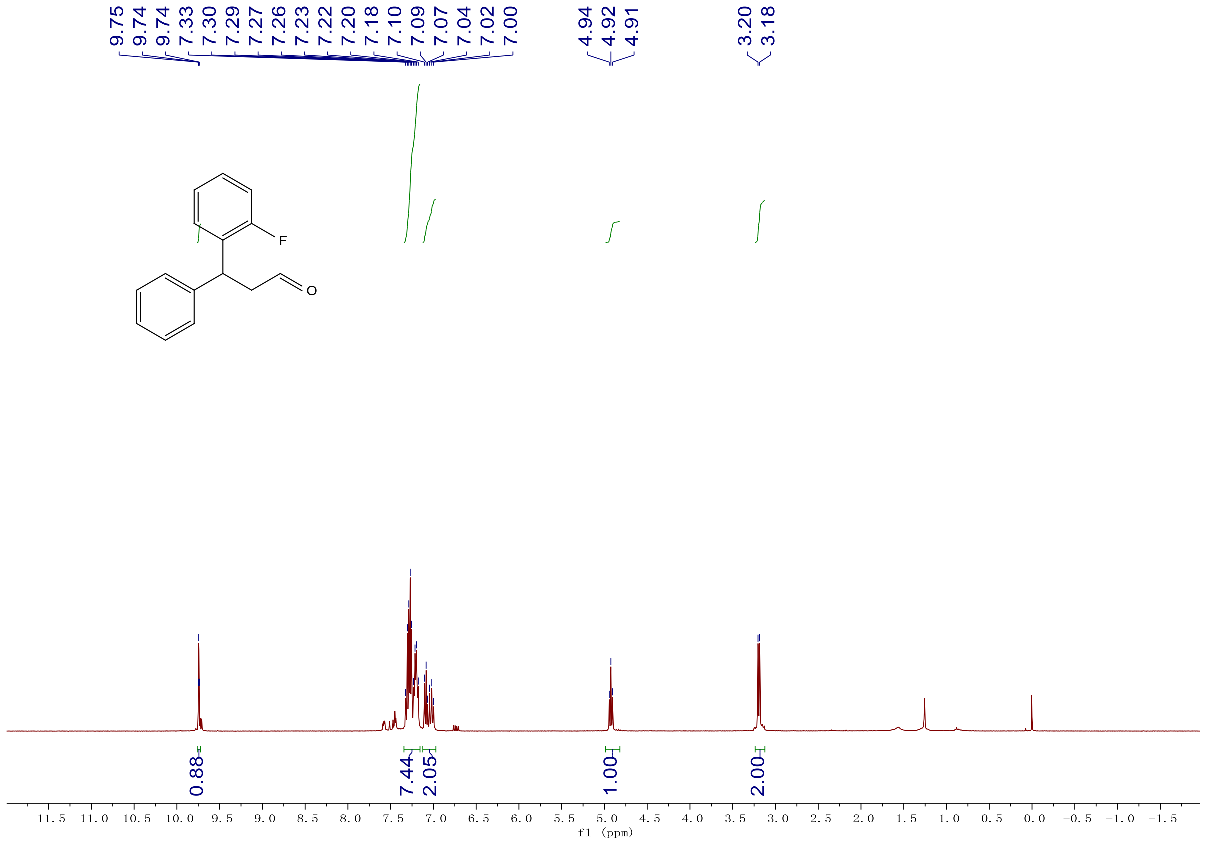

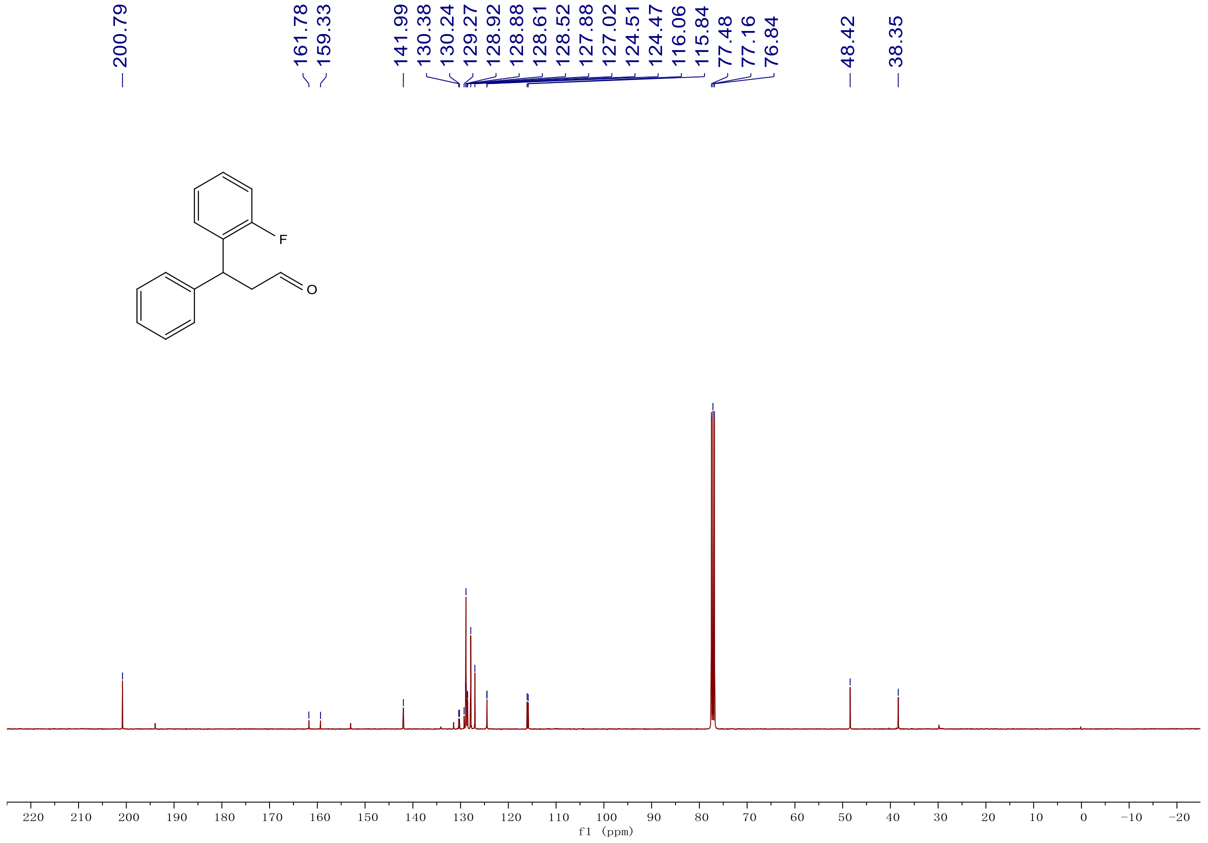

3,3-bis(4-bromophenyl)propanal (4e)

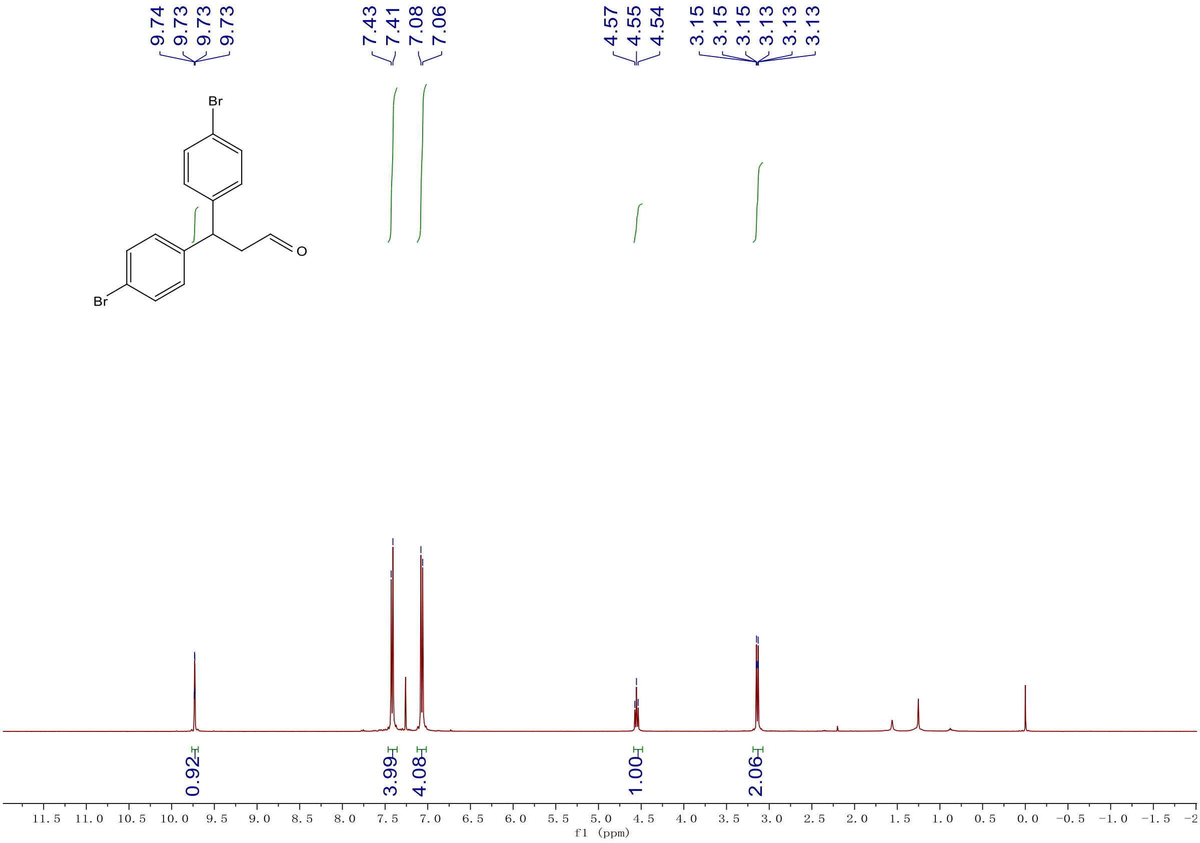

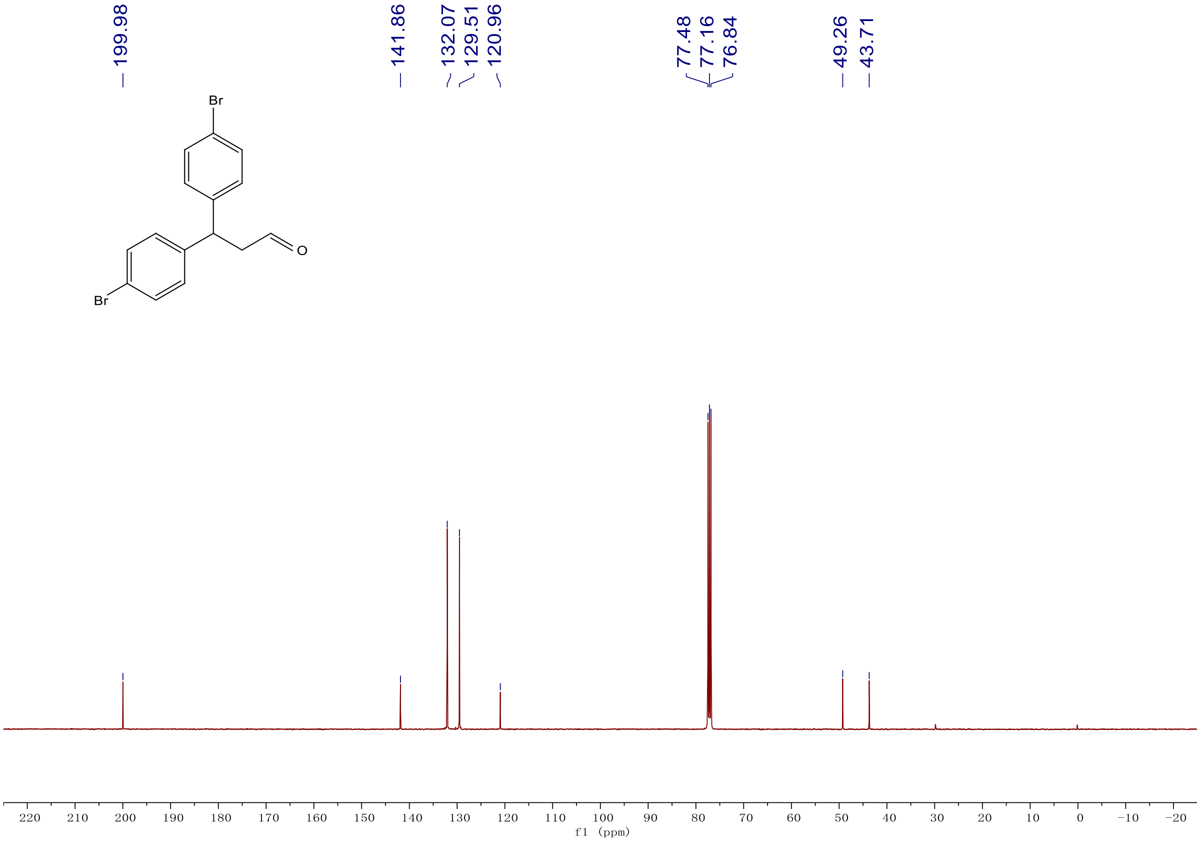

3-(4-bromophenyl)-3-(3-methoxyphenyl)propanal (4f)

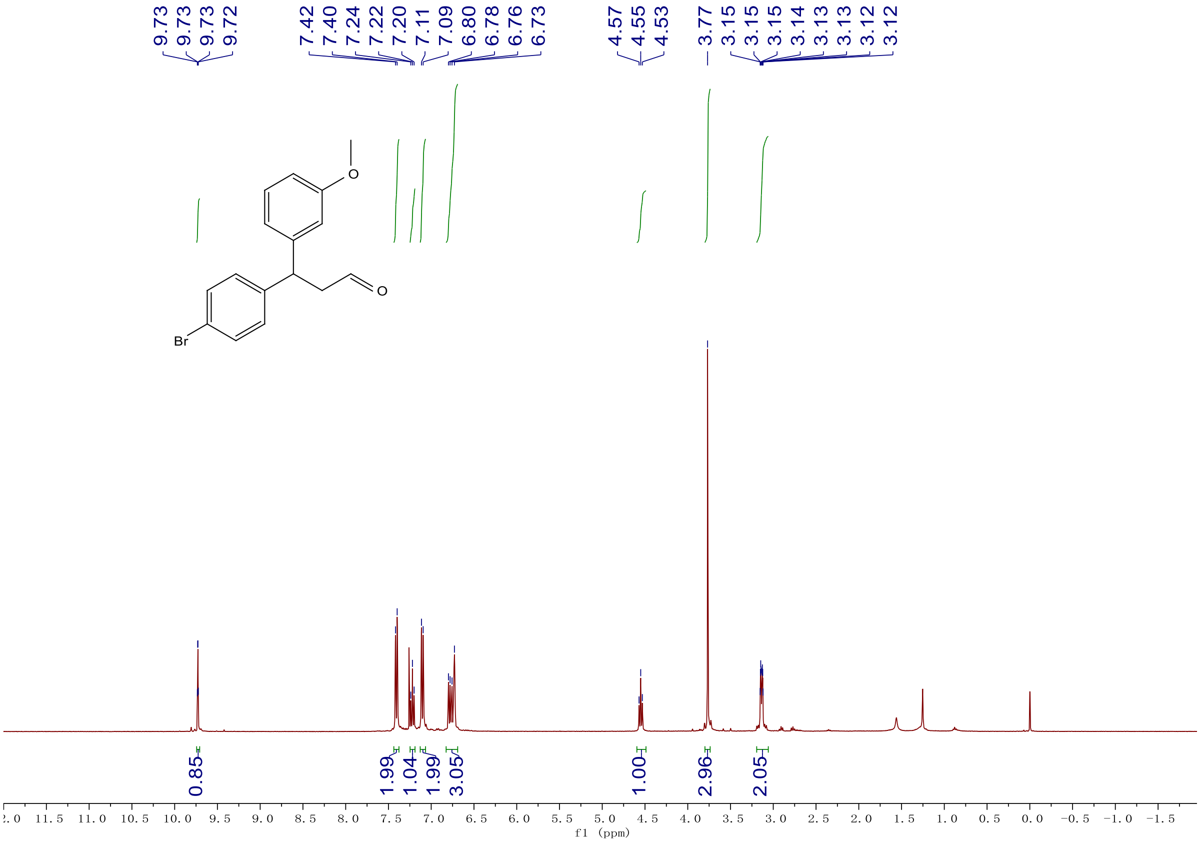

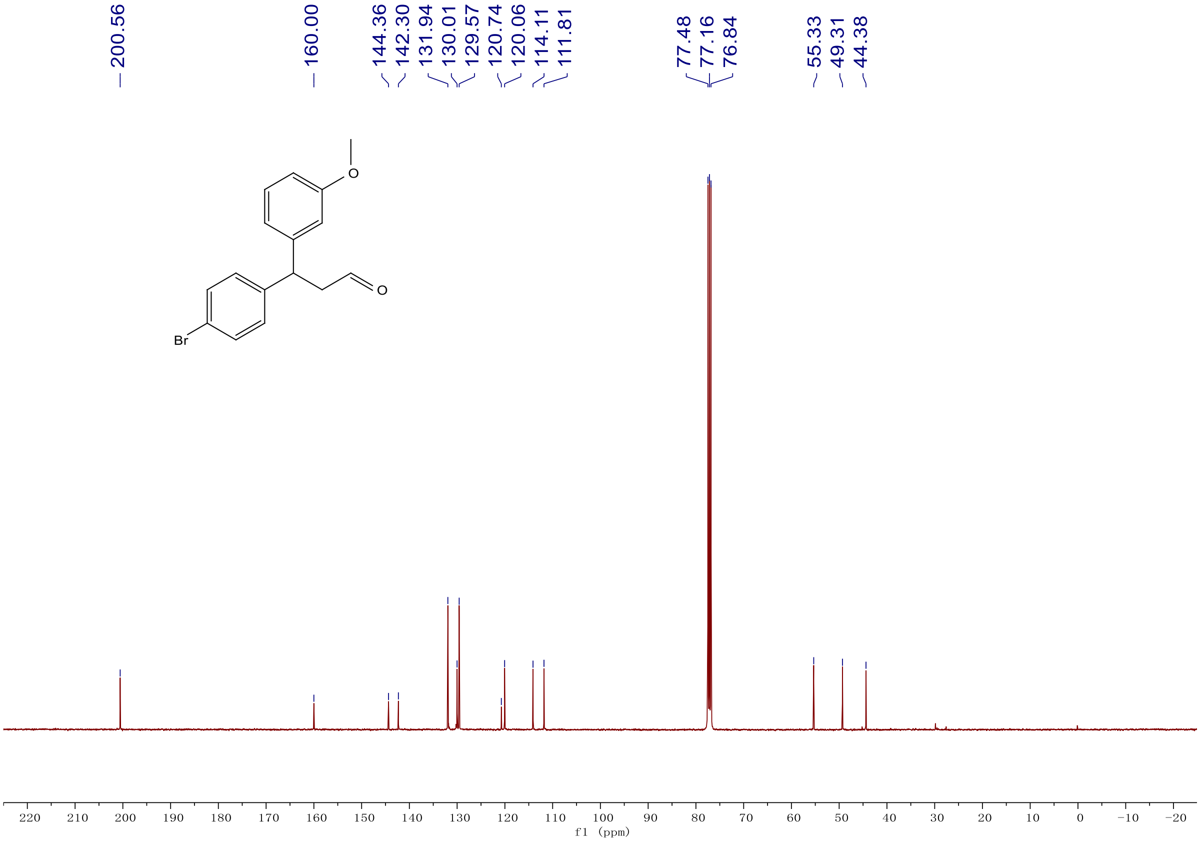

3-(4-(benzyloxy)phenyl)-3-(4-bromophenyl)propanal (4g)

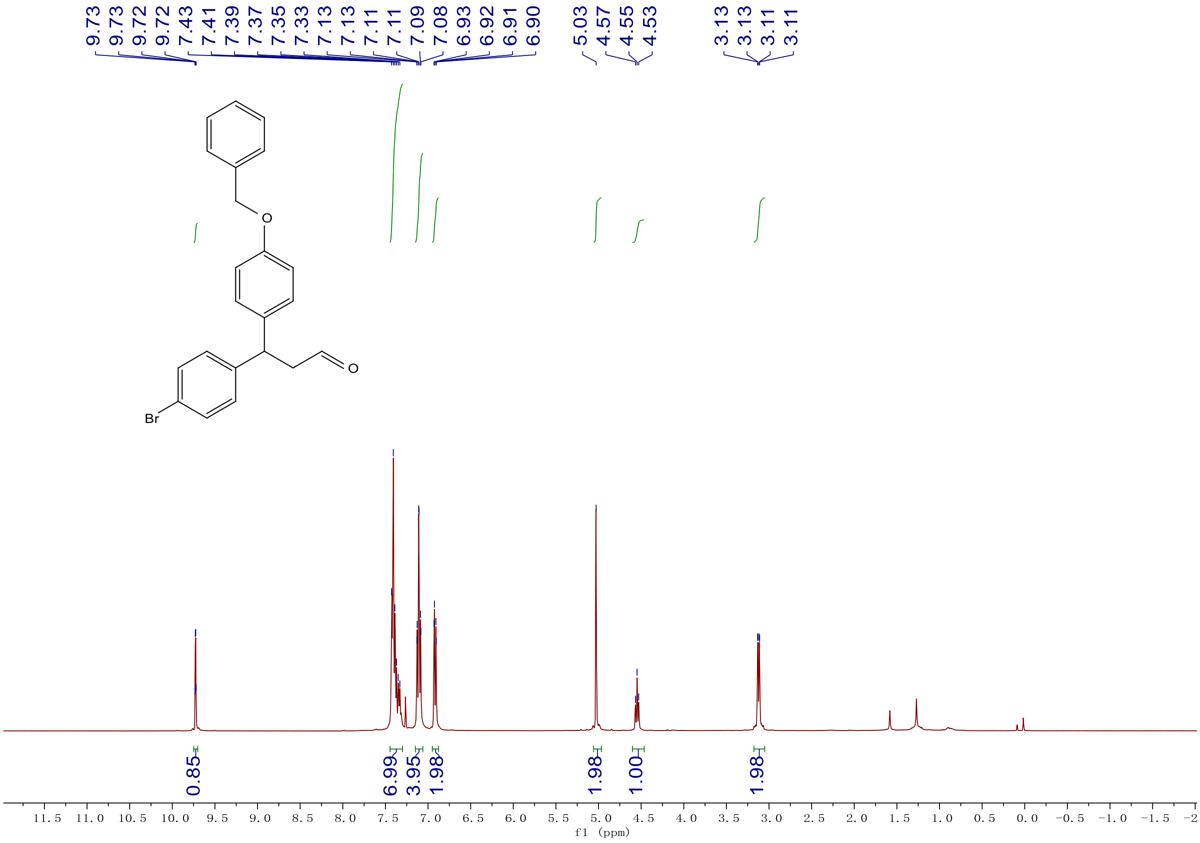

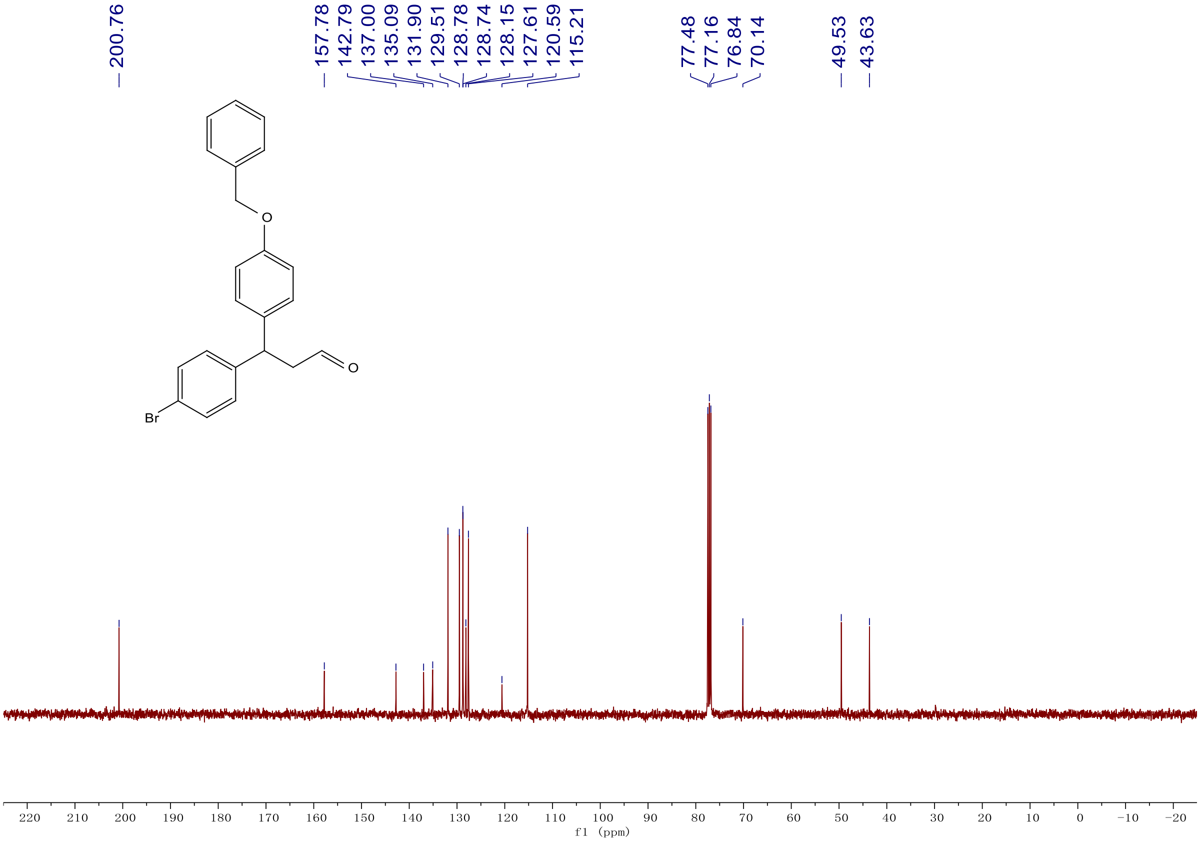

3-(4-methoxyphenyl)pentanal (5)

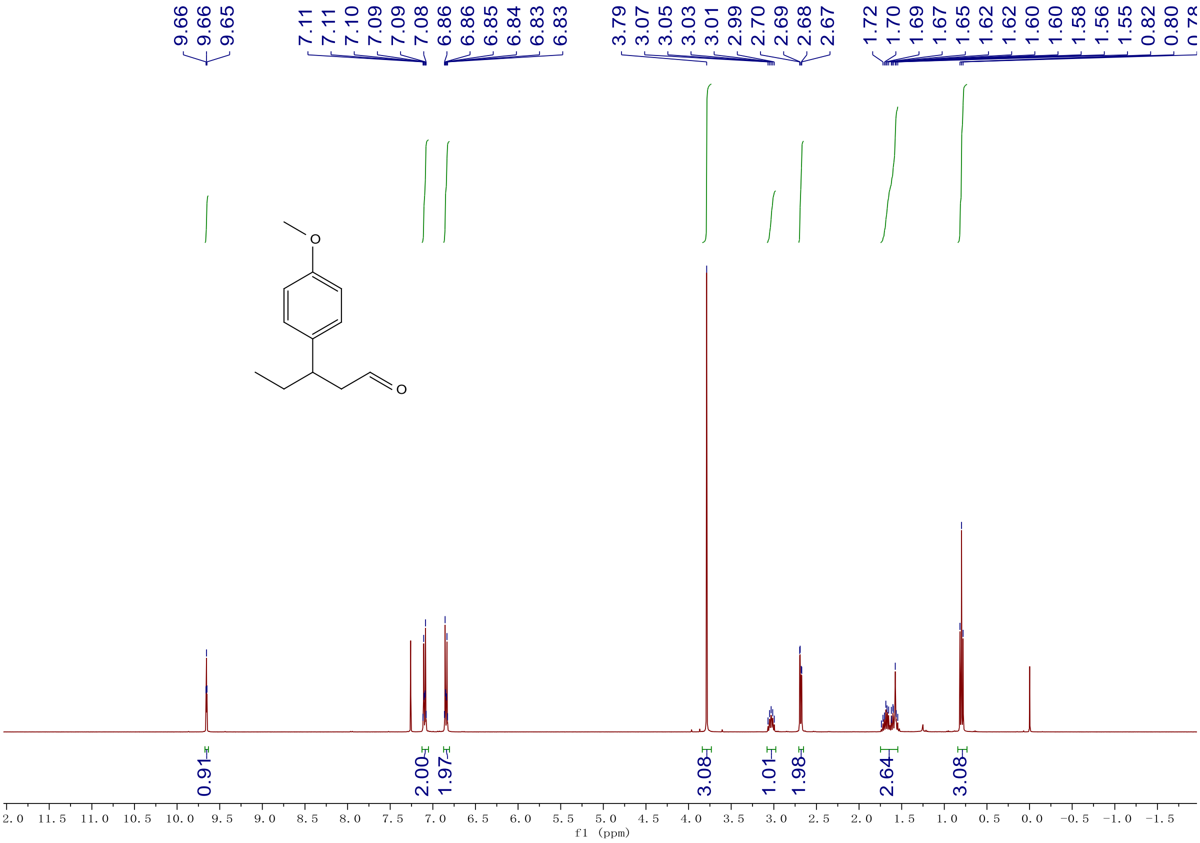

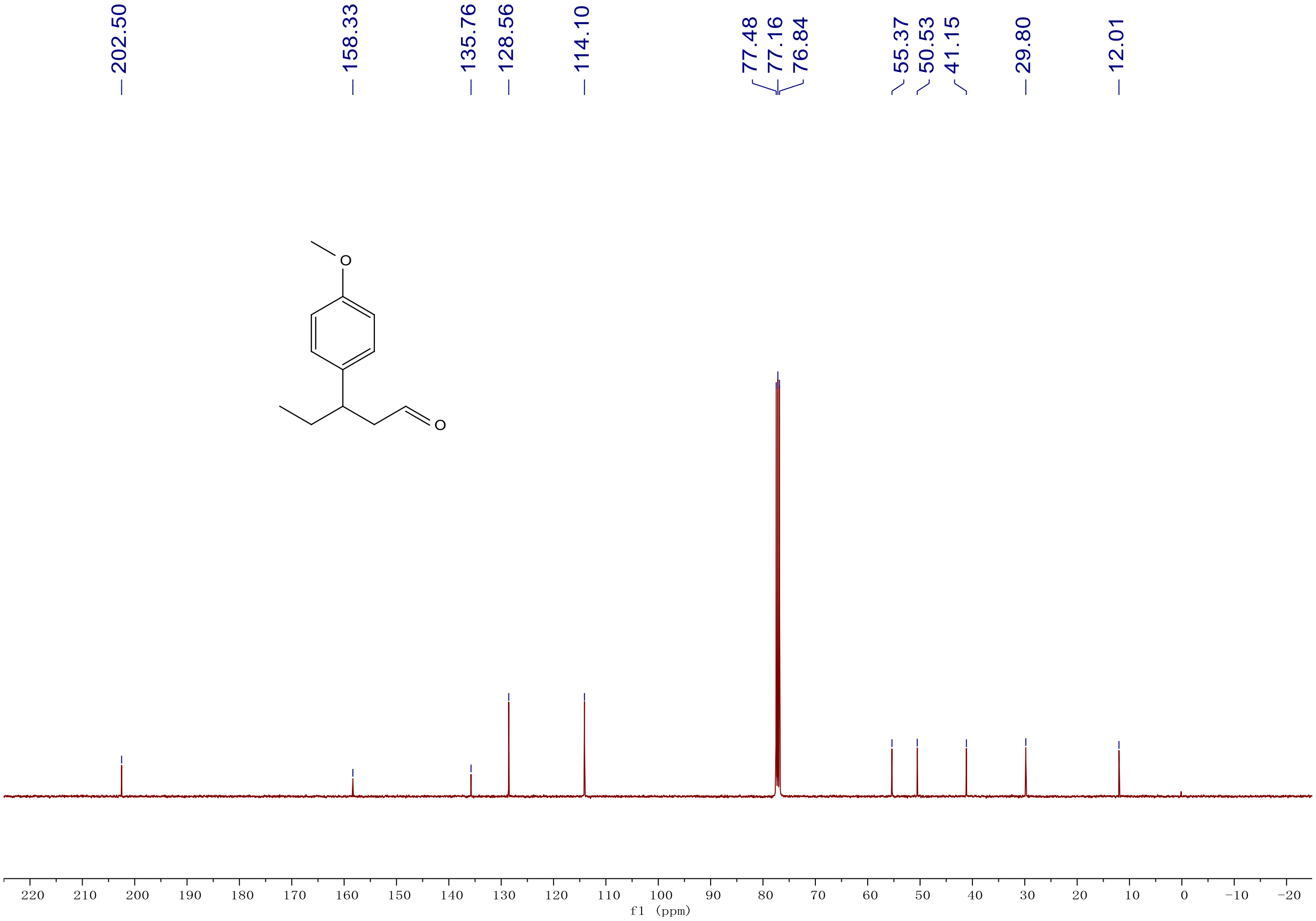

Supplement: SC-016-D5SC02745D-s001 [file SC-016-D5SC02745D-s001.pdf]
